# Supplementary material for: Community determinants of COPD exacerbations in elderly patients in Lodz province, Poland: a retrospective observational Big Data cohort study
Source: BMJ Open. 2022 Oct 21;12(10):e060247. doi: 10.1136/bmjopen-2021-060247 (PMC9594524; doi:10.1136/bmjopen-2021-060247)
Supplement: Supplementary data [file bmjopen-2021-060247supp002.pdf]

APPENDIX

Table 1A. Descriptive characteristics of cohort of COPD elderly patients within Lodz province (Raw data before PSM, n=16 099) and prevalence of COPD, including prevalence of COPD exacerbations (n=17 240, including deaths).

Characteristic	COPD without exacerbations (n=14 607)	COPD with exacerbations (n=1 492)	Total (n=16 099)	Odds Ratio Estimates Point Estimate 95% Wald Confidence Limits OR [95% CI] - Univariate analysis# HP Logistic Regression
**Total J44 Patients Sum (%)**	14 607 (90.73)	1 492 (9.27)	16 099	
**Exacerbation number**	0	1	Total	Data only for the group with exacerbations.
**1**	0 (0)	1 294 (86.73)	1 294 (8.04)	
**2**	0 (0)	148 (9.92)	148 (0.92)	
**3**	0 (0)	32 (2.14)	32 (0.2)	
**4**	0 (0)	11 (0.74)	11 (0.07)	
**5**	0 (0)	5 (0.34)	5 (0.03)	
**6**	0 (0)	2 (0.13)	2 (0.01)	
**Exacerbation number**				
**(min. - max.)**	0	(1 - 6)	(1 - 6)	
**Missing**	14 607	0	14 607	Data only for the group with exacerbations.
**Total (including Missing)**	14 607 (90.73)	1 492 (9.27)	16 099 (100)	
**Age of the patient 65+Median(q1 - q3)**	73 (68 - 80)	75 (69 - 81)	74 (69 - 80)	1.016 [1.009, 1.023]

Kowalczyk A, *et al. BMJ Open* 2022; 12:e060247. doi: 10.1136/bmjopen-2021-060247

				**Continuous var.
**Number of COPD consultations (PHC)**				
**(min. - max.)**	(0 - 24)	(0 - 20)	(0 - 24)	
**Number of consultations in PHC (min. - max.) **Continuous var.**	(0 - 78)	(0 - 59)	(0 - 78)	
**Drugs## Number of packages Avg±SD**				
**R03AC***	5±5.7	6±6.95	5±5.86	1.036 [1.028,1.044] **Continuous var.
**R03AK***	2±3.83	2±3.99	2±3.84	1.022 [1.008,1.035] **Continuous var.
**R03AL***	1±(5.39	3±8.99	1±5.89	1.041 [1.034, 1.048] **Continuous var.
**R03BA***	2±4.51	4±5.72	2±4.66	1.043 [1.034, 1.053] **Continuous var.
**R03BB***	5±6.11	6±7.3	5±6.27	1.037 [1.029, 1.044] **Continuous var.
**R03DA***	3±4.99	4±5.12	3±5.02	1.038 [1.028, 1.048] **Continuous var.
**R03DC***	0±1.68	0±1.52	0±1.67	0.985 [0.952, 1.020] **Continuous var.
**COST OF MEDICINES (Drugs) [%]**	Percent of median cost per COPD with exacerbations/median cost per COPD without exacerbations [%]			

**Total Fund Cost (Drugs) in [%]NMiss=1906 (11.84%)**	144.69%			HP LR estimate= 0.000316 **Continuous var.
**Income per post code NMiss=99 Difference between median COPD with exacerbations and median without exacerbations [PLN]**	584.02 PLN			**Continuous
**Did the patient die in 2016?**				
**No**	14 607 (94.51)	1 492 (83.63)	16 099 (93.38)	
**Yes**	849 (5.49)	292 (16.37)	1 141 (6.62)	
**Total**	15 456 (89.65)	1 784 (10.35)	17 240 (100)	
**Physicians'characteristics in PHC in Lodz Voivodeship**				
**Physicians'Age**				
**Median(q1-q3)**	52 (43.61)			
**Number**	2 465			
**Physician's sex**				
**Women=0**	1 674 (67.91)			
**Men=1**	791 (32.09)			
**Total**	2 465 (100)			
**Physician's spacialization**				
**Other=0**	1 838 (74.56)			
**Family doctor=1**	627 (25.44)			
**Total**	2 465 (100)			
**Total Physicians in Lodz voivodeship****	2 465 (100)			
**Prevalence**				
**Prevalence of total of COPD n=17240 among 65+ n=472 314 in Lodz Voivodeship, in PHC in 2016 (including deaths) = 3.65%, 95%CI (3.60-3.70) [%].**				
**Prevalence of total of exacerbations n=1784 among COPD 65+ n=17240 in Lodz Voivodeship, in PHC in 2016 (including death)= 10.35%, 95%CI (9.89-10.80) [%].**				

Kowalczyk A, *et al. BMJ Open* 2022; 12:e060247. doi: 10.1136/bmjopen-2021-060247

Patient Characteristic	COPD without exacerbations (n=15 456)	COPD with exacerbations (n=1 784)	Total (n=17 240)	OR (95% CI) - Univariate analysis Logistic Regression
**Total Patients COPD 65+ including deaths**	15 456 (89.65)	1 784 (10.35)	17 240 (100)	
**Sex**				
**Female**	7 256 (46.95)	708 (39.69)	7 964 (46.19)	F Ref. Cat.
**Male**	8 200 (53.05)	1 076 (60.31)	9 276 (53.81)	1.345 [1.217,1.486]
**Total**	15 456 (89.65)	1 784 (10.35)	17 240 (100)	

*Data are presented as n (%), mean ±SD or median (q1 - q3)..
#Crude, Univariate Model, OR (95% CI), Odds Ratio with 95% Wald, Confidence Limits using proc logistic, proc hplogistic.
**Lodz Voivodeship known as Lodz province (Wikipedia): https://en.wikipedia.org/wiki/%C5%81%C3%B3d%C5%BA_Voivodeship
ATC group - Anatomical Therapeutic Chemical (ATC) Classification
R03 - drugs for obstructive airway diseases
R03A - adrenergics, inhalants:
*R03AC - Selective beta-2-adrenoreceptor agonists
*R03AK - Adrenergics in combination with corticosteroids or other drugs, excl. anticholinergics
*R03AL - Adrenergics in combination with anticholinergics incl. triple combinations with corticosteroids
R03B - other drugs for obstructive airway diseases, inhalants:
*R03BA – Glucocorticoids
*R03BB - Anticholinergics
R03D - other systemic drugs for obstructive airway diseases:
*R03DA - Xanthines
*R03DC - Leukotriene receptor antagonists
https://indeks.mp.pl/leki/find.php?kind=atc&subatc=1&id=R03
- NMiss=1906 (11.84%) of missing data per each of the 7 variables with drugs:*R03AC, *R03AK, *R03AL, *R03BA, *R03BB, *R03DA, *R03DC.

Kowalczyk A, *et al. BMJ Open* 2022; 12:e060247. doi: 10.1136/bmjopen-2021-060247

Table 2A Characteristic of COPD patients with and without exacerbations (After PSM).

Characteristic	COPD patients without exacerbations (n=1 492 pairs) Control (J44) n(%)	COPD patients with exacerbations (n=1 492 pairs) Case (Hospitalized due to J44) n(%)	Total patients (n=2 984) n(%)	OR (95% Wald Confidence Limits). (Point Estimate.) OR (95% CI) - Univariate analysis# Paired LR
**Total**	1 492 (50)	1 492 (50)	2 984 (100)	
**Exacerbation number**				Data only for the group with exacerbations.
**1**	0 (0)	1 294 (86.73)	1 294 (8.04)	
**2**	0 (0)	148 (9.92)	148 (0.92)	
**3**	0 (0)	32 (2.14)	32 (0.2)	
**4**	0 (0)	11 (0.74)	11 (0.07)	
**5**	0 (0)	5 (0.34)	5 (0.03)	
**6**	0 (0)	2 (0.13)	2 (0.01)	
**Total**	1 492 (50)	1 492 (50)	2 984 (100)	
**Age of the patient 65+ (years) Median(q1-q3)**	75 (69.00- 81.00)	75 (69.00-81.00)	75 (69.00-81.00)	PSM NS LR No Effect after PSM
**Number of refunded drugs packages (ATC groups) (Missing 7.37%) Avg±SD)**				
**R03AC***	4.65±±5.63	6.08±6.95	5.4±6.39	1.037 [1.023,1.051]
**R03AK***	2.01±3.9	2.46±3.99	2.25±3.95	1.028 [1.007,1.049]
**R03AL***	0.95±4.18	3.41±8.99	2.24±7.21	1.087 [1.064,1.11]
**R03BA***	2.38±4.25	3.55±5.72	3±5.1	1.051 [1.033,1.069]
**R03BB***	4.47±5.83	6.38±7.3	5.46±6.71	1.049 [1.035,1.064]
**R03DA***	3.06±5.12	4.01±5.12	3.56±5.14	1.038 [1.022,1.055]
**R03DC***	0.25±1.45	0.28±1.52	0.26±1.49	0.997 [0.945,1.051]
**Total NFZ cost for, all current and settled services in 2016, regardless of the main diagnosis and reimbursement of drugs from prescriptions completed in 2016**				
**high (>21 056.52)**	186 (12.47)	560 (37.53)	746 (25)	42.804 [28.145,65.097]
**medium <2 497.62-8 264.65>**	618 (41.42)	875 (58.65)	1 493 (50.03)	18.503 [12.627,27.115]
**low (<2 497.62)**	688 (46.11)	57 (3.82)	745 (24.97)	1 Ref. Cat.
**Total**	1 492 (50)	1 492 (50)	2 984 (100)	

*Data are presented as n (%), mean ±SD or median (q1q3).
IQR – interquartile range

Kowalczyk A, *et al. BMJ Open* 2022; 12:e060247. doi: 10.1136/bmjopen-2021-060247

Table 3A Costs for the raw data (Raw data before PSM, n=16 099) of COPD patients with and without exacerbations.

Cost indicators (%) were calculated as contribution of patient costs with exacerbations (or without) among the cost for all COPD patients.

**Characteristic Costs n(%) N=16099**	**COPD without exacerbations (n=14 607)**	**COPD with exacerbations (n=1 492)**	**Total (n=16 099)**	**p-value - Univariate analysis# HP Logistic Regression**
**Total J44 Patients Sum (%)**	14 607 (90.73)	1 492 (9.27)	16 099	
**Total stationary costs (%)***				
**high**	3189 (21,83)	838 (56,17)	4027 (25,01)	<.0001
**medium**	1756 (12,02)	642 (43,03)	2398 (14,9)	<.0001
**low**	9662 (66,15)	12 (0,8)	9674 (60,09)	Ref. Cat.
**Total**	14607 (90,73)	1492 (9,27)	16099 (100)	
**Total cost of reimbursed drugs (%)***				
**high**	3434 (23,51)	590 (39,54)	4024 (25)	<.0001
**medium**	7363 (50,41)	687 (46,05)	8050 (50)	<.0001
**low**	3810 (26,08)	215 (14,41)	4025 (25)	Ref. Cat.
**Total**	14607 (90,73)	1492 (9,27)	16099 (100)	
**TOTAL NFZ cost (%)***				
**high**	3191 (21,85)	833 (55,83)	4024 (25)	<.0001
**medium**	7398 (50,65)	653 (43,77)	8051 (50,01)	<.0001
**low**	4018 (27,51)	6 (0,4)	4024 (25)	Ref. Cat.
**Total**	14607 (90,73)	1492 (9,27)	16099 (100)	

*Costs were calculated as the proportion of costs per patient in the mean of the COPD patient population [%], and categorized by lower (q1) and upper (q3) quartiles.
q1-Lower Quartile; q3-Upper Quartile, CL-Confidence Limit, HP LR-High Performance Logistic Regression.
N=16099
Total stationary costs (%) q1=0.00 q3=101.72
Total cost of reimbursed drugs (%) q1=34.75 q3=141.10
TOTAL NFZ cost (%) q1=28.71 q3=111.69

Kowalczyk A, *et al. BMJ Open* 2022; 12:e060247. doi: 10.1136/bmjopen-2021-060247

Table 3B Costs for the data after PSM transformation (N=1492 pairs) of COPD patients with and without exacerbations.

Characteristic Costs n(%) N=1492 pairs	COPD patients without exacerbations (n=1492 pairs) n(%)	COPD patients with exacerbations exacerbations (n=1492 pairs) n(%)	TotalAll patients (n=2984) n(%)	p-value - Univariate analysis Conditional Logistic Regression for Matched Pairs
**Total J44 Patients Sum (%)**	1492 (50)	1492 (50)	2984 (100)	
**Total stationary costs (%)***				
**high**	168 (11,26)	564 (37,8)	732 (24,53)	<.0001
**medium**	332 (22,25)	916 (61,39)	1248 (41,82)	<.0001
**low**	992 (66,49)	12 (0,8)	1004 (33,65)	Ref. Cat.
**Total**	1492 (50)	1492 (50)	2984 (100)	
**Total cost of reimbursed drugs (%)***				
**high**	270 (18,1)	476 (31,9)	746 (25)	<.0001
**medium**	748 (50,13)	744 (49,87)	1492 (50)	<.0001
**low**	474 (31,77)	272 (18,23)	746 (25)	Ref. Cat.
**Total**	1492 (50)	1492 (50)	2984 (100)	
**TOTAL NFZ cost (%)***				
**high**	186 (12,47)	560 (37,53)	746 (25)	<.0001
**medium**	618 (41,42)	875 (58,65)	1493 (50,03)	<.0001
**low**	688 (46,11)	57 (3,82)	745 (24,97)	Ref. Cat.
**Total**	1492 (50)	1492 (50)	2984 (100)	

*Costs were calculated as the proportion of costs per patient in the mean of the COPD patient population [%], and categorized by lower (q1) and upper (q3) quartiles.
q1-Lower Quartile; q3-Upper Quartile, CL-Confidence Limit.
N=1492 pairs
Total stationary costs (%) q1=0.00 q3=111.56
Total cost of reimbursed drugs (%) q1=37.19 q3=142.19
TOTAL NFZ cost (%) q1=34.23 q3=113.27

Kowalczyk A, *et al. BMJ Open* 2022; 12:e060247. doi: 10.1136/bmjopen-2021-060247

Big Data Quality (Big Data Cleansing) Results: for Medical (NFZ) and Business (Tax Office) Data.

Files were obtained from Big Data databases (NFZ, Tax Office and GUS). In total, data for n = 472,314 cohorts of 65+ patients registered in primary health care (PHC) clinics were obtained from electronic medical records (EHR) of NFZ in the Lodz province. In total, n = 439,577 inhabitants 65+ living in the Lodz province were obtained from the databases of 28 Tax Offices located in the Lodz province. Variables for n = 177 communes of the Lodz province, which, according to the literature review, were related to exacerbations were collected from the GUS databases. Patient data from NFZ showed that the identifier has no duplicates. "Codes of comorbidities with COPD" had 98.8% of missing data (NFZ) out of 472 314 in total 65+ patients in PHC in Lodz province in 2016, and the variable "ICD9 procedures for other stays" had 98.1% of missing data (NFZ). Only 0.1% was missing in the variable "Postal code for 65+ residents" in Tax Office databases. Data on air quality for the group "Air pollutant emission from particular nuisance plants (G216)" consisting of 6 subgroups were not available at the level of data sharing for gmina level for 2016 in the GUS database (accessed on November 3, 2021) .

Propensity score matching (PSM) - before and after - overlay histograms age, gender

Fig. 1 Histogram for the age before PSM.

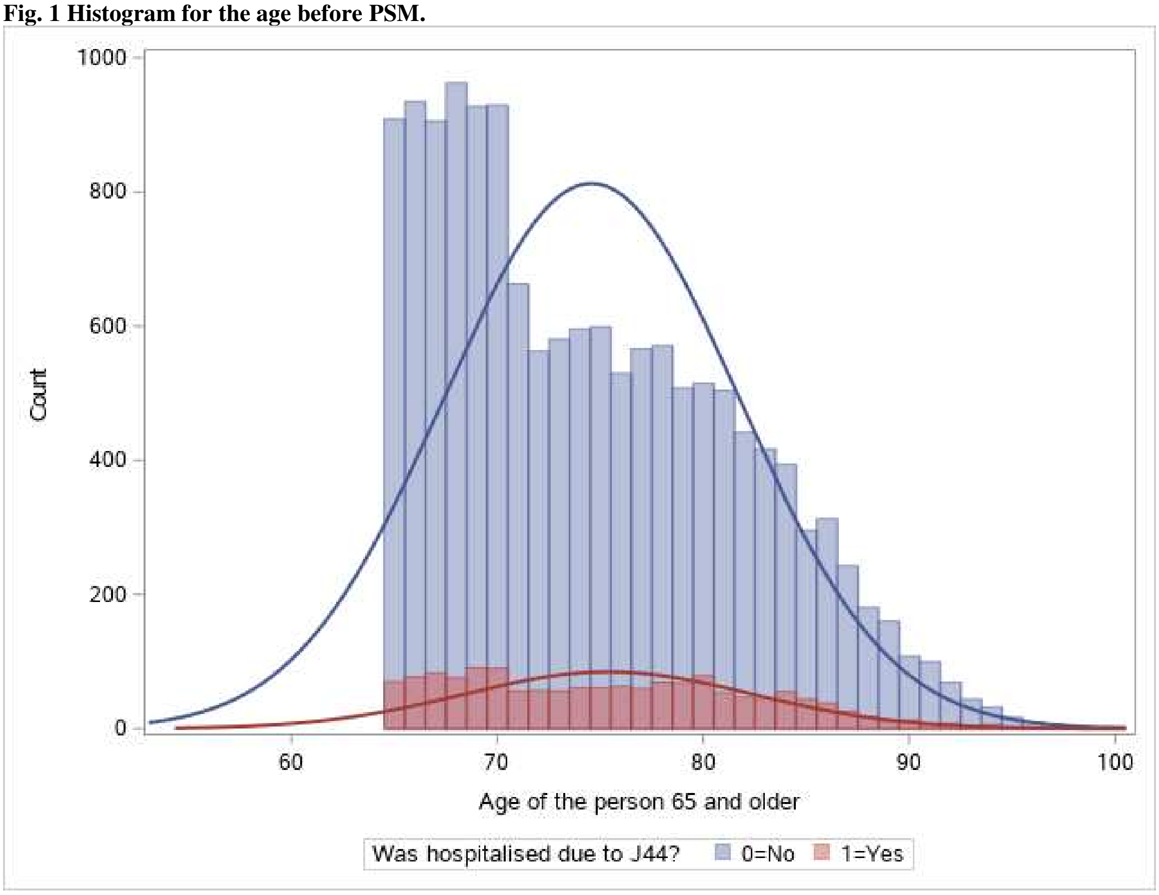

Kowalczyk A, *et al. BMJ Open* 2022; 12:e060247. doi: 10.1136/bmjopen-2021-060247

Fig. 2 Histogram for the age after PSM.

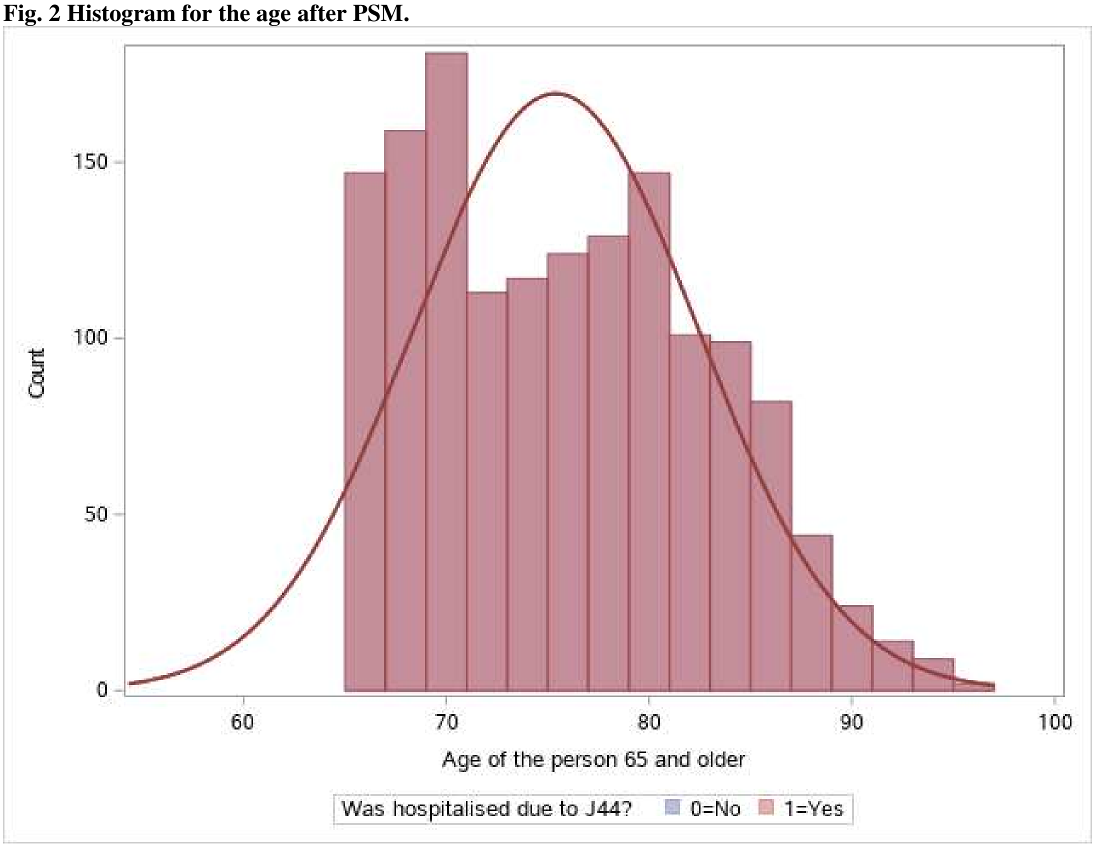

Kowalczyk A, *et al. BMJ Open* 2022; 12:e060247. doi: 10.1136/bmjopen-2021-060247

Fig. 3 Histogram for the gender before PSM.

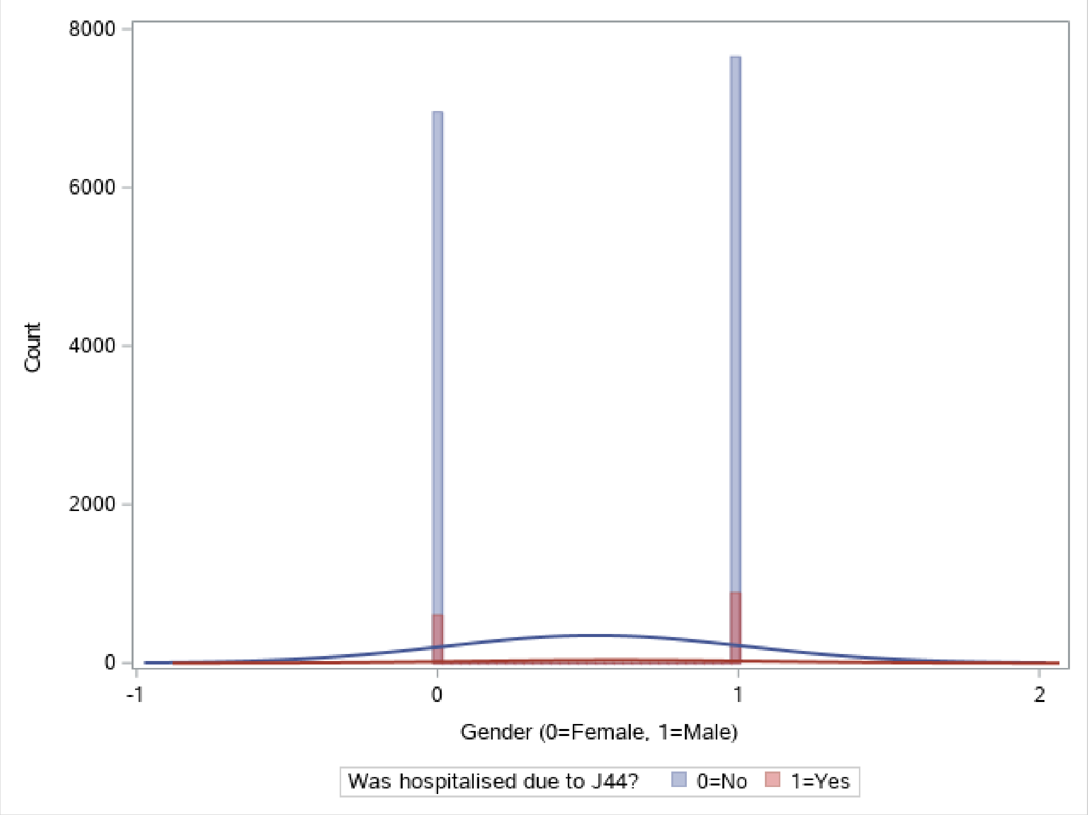

Fig. 4 Histogram for the gender after PSM.

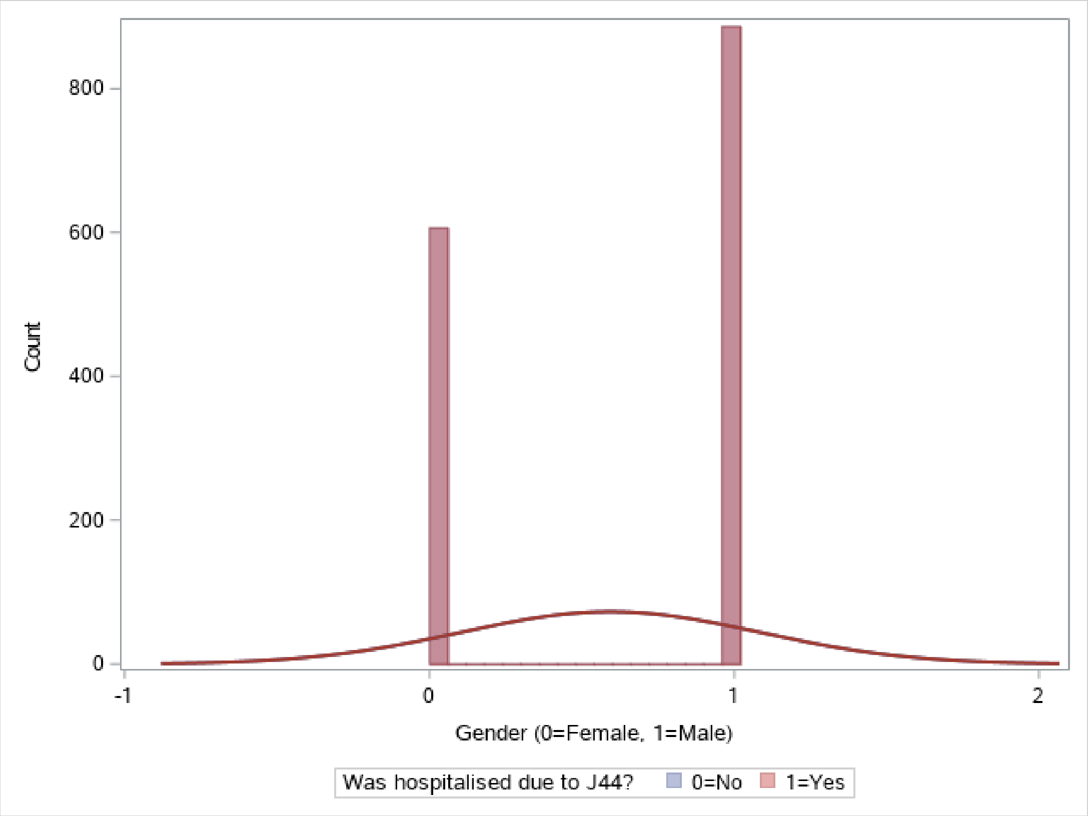

Kowalczyk A, *et al. BMJ Open* 2022; 12:e060247. doi: 10.1136/bmjopen-2021-060247
