# Supplementary material for: Community determinants of COPD exacerbations in elderly patients in Lodz province, Poland: a retrospective observational Big Data cohort study
Source: BMJ Open. 2022 Oct 21;12(10):e060247. doi: 10.1136/bmjopen-2021-060247 (PMC9594524; doi:10.1136/bmjopen-2021-060247)
Supplement: Supplementary data [file bmjopen-2021-060247supp001.pdf]

Supplementary: Table 1S Descriptive characteristics and univariate analysis of COPD exacerbations in cohort of COPD elderly patients and prevalence of COPD in PHC (Lodz province in 2016). (Raw data before PSM).

**Patient Characteristic (Before PSM)**	**COPD patients without exacerbations (n=14607)**	**COPD patients with exacerbations (n=1492)**	**Total (n=16099)**	**OR (95% CI), p-value #Univariate Logistic Regression.**
Patient level				
Demographic				
COPD Patients (Sum)(%) Total	14607 (90.73)	1492 (9.27)	16099 (100)	
Sex				
Male	7652(52.39)	886(59.38)	8538(53.03)	1.329(1.192-1.481)
Female	6955(47.61)	606(40.62)	7561(46.97)	1 Ref. Cat.
Age (years)				
<75-85)	5046(34.55)	600(40.21)	5646(35.07)	1.322(1.18-1.482)
<85+>	1587(10.86)	175(11.73)	1762(10.94)	1.226(1.031-1.459)
<65-75)	7974(54.59)	717(48.06)	8691(53.98)	1 Ref. Cat.
Patient’s place of residence in gmina (localization)				
1-urban	8971(61.42)	864(57.91)	9835(61.09)	0.840(0.728-0.971)
2-rural	3280(22.45)	358(23.99)	3638(22.6)	0.952(0.806-1.125)
3-urban-rural	2356(16.13)	270(18.1)	2626(16.31)	1 Ref. Cat.
Healthcare uses				
Specialization of physician working in PHC				
Family doctors	5483(37.54)	587(39.34)	6070(37.7)	1 Ref. Cat.
Other	9124(62.46)	905(60.66)	10029(62.3)	0.926(0.831-1.033)
Number of consultations in PHC^				
high (>12)	5309(36.35)	689(46.18)	5998(37.26)	1.277(1.063-1.535)
medium <5-12>	7783(53.28)	649(43.5)	8432(52.38)	0.820(0.682-0.986)

low (<5)	1515(10.37)	154(10.32)	1669(10.37)	1 Ref. Cat.
Number of COPD consultations in PHC^				
0	166 (1.14)	548 (36.73)	714 (4.44)	21.718 (17.658-26.712)
1	7701 (52.72)	286 (19.17)	7987 (49.61)	0.244 (0.208-0.288)
2	2930 (20.06)	191 (12.8)	3121 (19.39)	0.429 (0.357-0.516)
3	1514 (10.36)	118 (7.91)	1632 (10.14)	0.513 (0.412-0.638)
4+	2296 (15.72)	349 (23.39)	2645 (16.45)	1 Ref. Cat.
Gmina/Commune, post-codes level				
Socioeconomic Status				
Total personal income of residents per PHC post-codes (Tax Office)				
high (>21992,62)	3717 (25.45)	240 (16.09)	3957 (24.58)	0.573 (0.486-0.677)
medium <17612,68-21992,62>	7231 (49.5)	840 (56.3)	8071 (50.13)	1.032 (0.911-1.168)
low (<17612,68)	3659 (25.05)	412 (27.61)	4071 (25.29)	1 Ref. Cat.
Environmental factors				
Forest cover of the patient's place of residence in gmina (GUS)				
high (>17,10%)	3043 (20.83)	308 (20.64)	3351 (20.81)	0.791 (0.686-0.913)
medium <8,40%-17,10%>	6583 (45.07)	547 (36.66)	7130 (44.29)	0.65 (0.576-0.733)
low (<8,40%)	4981 (34.1)	637 (42.69)	5618 (34.9)	1 Ref. Cat.
Total	14607 (90.73)	1492 (9.27)	(100)	

^Pearson Correlation Coefficient=0,2468, p<0.0001 between "Number of consultations in PHC" and "Number of COPD consultations in PHC". CI=Confidence Interval, OR=Odds Ratio. #=Odds Ratio Estimates (Point Estimate). 95% Wald Confidence Limits. OR (95% CI). #Univariate analysis Univariate Logistic Regression. ##Univariate High-Performance Logistic Regression

Table 2S. Descriptive characteristics and local determinants of COPD exacerbations in cohort of COPD elderly patients (Lodz province, 2016; case-control). After PSM.

**Patient Characteristic (After PSM)**	**COPD patients without exacerbations Control (n=1492 pairs)**	**COPD patients with exacerbations Cases (n=1492 pairs)**	**Total (n=2984)**	**OR (95% CI) Univariate analysis****	**OR (95% CI)**
**Patient level**					
**Demographic**					
**COPD Patients (Sum)(%) Total**	1492 (50)	1492 (50)	2984 (100)		
**Sex (strata variable)**					
**Male**	886 (59.38)	886 (59.38)	1772 (59.38)	1 Ref. Cat^	Strata variable
**Female**	606 (40.62)	606 (40.62)	1212 (40.62)	1.000 (0.864-1.157)^	
**Age (years) (strata variable)**					
**<75-85)**	600(40.21)	600(40.21)	1200(40.21)	1.000 (0.788-1.269)^	Strata variable
**<85+>**	175(11.73)	175(11.73)	350(11.73)	1 Ref. cat.^	
**<65-75)**	717(48.06)	717(48.06)	1434(48.06)	1.000 (0.792-1.263)^	
**Patient's place of residence in gmina (localization)**					
**1-urban vs. 3**	862 (57.77)	864 (57.91)	1726 (57.84)	1.028 (0.848-1.246)	1.044 (0.673-1.620)
**2-rural vs. 3**	353 (23.66)	358 (23.99)	711 (23.83)	1.04 (0.833-1.3)	0.897 (0.630-1.277)
**3-urban-rural (Ref. Cat.)**	277 (18.57)	270 (18.1)	547 (18.33)	1 Ref. Cat.	1 Ref. Cat.
**Healthcare uses**					
**Specialization of physician working in PHC**					
**Family doctors vs. Other**	562(37.67)	587(39.34)	1149(38.51)	1.080(0.926-1.259)	1.076 (0.920-1.257)
**Other (Ref. Cat.)**	930(62.33)	905(60.66)	1835(61.49)	1 Ref. Cat.	1 Ref. Cat.
**Number of consultations in PHC^**					
**high (>12) vs. low (<5)**	558 (37.4)	689 (46.18)	1247 (41.79)	1.269 (0.982-1.639)	1.261 (0.974-1.633)

**medium <5-12> vs. low (<5)**	783 (52.48)	649 (43.5)	1432 (47.99)	0.831 (0.651-1.061)	0.823 (0.643-1.053)
**low (<5) (Ref. Cat.)**	151 (10.12)	154 (10.32)	305 (10.22)	1 Ref. Cat.	1 Ref. Cat.
**Number of COPD consultations in PHC^**					
**0 vs. 4+**	13 (0.87)	548 (36.73)	561 (18.8)	41.078 (18.064-93.413)	
**1 vs. 4+**	751 (50.34)	286 (19.17)	1037 (34.75)	0.288 (0.224-0.37)	
**2 vs. 4+**	299 (20.04)	191 (12.8)	490 (16.42)	0.488 (0.369-0.645)	
**3 vs. 4+**	185 (12.4)	118 (7.91)	303 (10.15)	0.528 (0.377-0.738)	
**4+ (Ref. Cat.)**	244 (16.35)	349 (23.39)	593 (19.87)	Ref. Cat.	
**Gmina/Commune, post-codes level**					
**Socioeconomic Status**					
**Total personal income of residents per PHC post-codes (Tax Office)**					
**high (>21056,52) vs. low (<17079,50)**	371 (24.87)	345 (23.12)	716 (23.99)	0.696 (0.489-0.990)	0.601 (0.385-0.939)
**medium <17079,50-21056,52> vs. low (<17079,50)**	759 (50.87)	768 (51.47)	1527 (51.17)	0.910 (0.722-1.147)	0.824 (0.581-1.169)
**low (<17079,50) (Ref. Cat.)**	362 (24.26)	379 (25.4)	741 (24.83)	1 Ref. Cat.	1 Ref. Cat.
**Environmental factors**					
**Forest cover of the patient's place of residence in gmina (GUS)**					
**high (>20,60%) vs. low (<7,00%)**	348 (23.32)	340 (22.79)	688 (23.06)	0.896 (0.648-1.24)	0.897 (0.605-1.331)
**medium <7,00%-20,60%> vs. low (<7,00%)**	776 (52.01)	776 (52.01)	1552 (52.01)	0.94 (0.688-1.285)	0.925 (0.648-1.322)
**low (<7,00%) (ref. Cat.)**	368 (24.66)	376 (25.2)	744 (24.93)	1 Ref. Cat.	1 Ref. Cat.
**Total**	1492 (50)	1492 (50)	2984 (100)		

CI=Confidence Interval, OR=Odds Ratio. #=Odds Ratio Estimates (Point Estimate). 95% Wald Confidence Limits. OR (95% CI).
Ref. cat.=reference category.
^- Sex and age were strata variables (the LOGISTIC Procedure, Logistic Regression).
**The LOGISTIC Procedure, Conditional Logistic Regression for Matched Pairs.

Descriptive characteristics of determinants

“Patient with ICD-10 code J44." - Determination whether J44 ICD-10 code is present in patients’ medical records in PHC clinics

“Exacerbations” - "Hospitalization because of J44." - Determination of whether the patient was “hospitalized with the J44 code as a main reason for admission. (Main diagnosis checked at the hospitalization level and not at individual wards).

“Patient’s place of residence in gmina” (localization) - based on patients’ PHC clinic address with assigned post- code

Division into the type of communes according to the Central Statistical Office: 1-urban, 2-rural, 3-urban-rural (Ref. Cat.). GUS Kind of gminas name: https://bdl.stat.gov.pl/BDL/metadane/teryt/rodzaj

“Specialization of physician working in PHC”: Family doctors, Other (Ref. Cat.) - Specialization of the physician working in PHC clinic within the Lodz province - family doctors, and other physicians (internal medicine specialist, pediatrician, physician without specialization). (NFZ database)

“Number of consultations in PHC”: high (>12), medium <5-12>, low (<5) (Ref. Cat.) - The number of patient’s consultations for any reason in PHC clinic in 2016

“Number of COPD consultations in PHC”: 0, 1, 2, 3, 4+ (Ref. Cat.) – the number of patient’s health care consultations with the main diagnosis J44in 2016

"Codes of diseases associated with COPD (PHC)" – codes of comorbidities in the medical record of a patient with COPD. in 2016

“Total personal income of residents per PHC post-codes” – (Tax Office): high (>21056,52 PLN), medium <17079,50 PLN -21056,52 PLN > , low (<17079,50 PLN) (Ref. Cat.)

“Forest cover of the patient's place of residence in gmina” (GUS): high (>20,60%), medium <7,00%-20,60%>, low (<7,00%) (ref. Cat.) in 2016

"The total cost of STAC" - The NFZ costs for all hospital services in 2016 for a given patient, regardless of the main diagnosis

"Total cost of drug reimbursement" - The NFZ costs for all prescribed medications in 2016 for a given patient

"Total NFZ cost" - The NFZ cost – (as sum of costs of PHC, ASC, hospitalization, drug reimbursement and other) in 2016 per patient, regardless of the main diagnosis
