# Supplementary material for: Community determinants of COPD exacerbations in elderly patients in Lodz province, Poland: a retrospective observational Big Data cohort study
Source: BMJ Open. 2022 Oct 21;12(10):e060247. doi: 10.1136/bmjopen-2021-060247 (PMC9594524; doi:10.1136/bmjopen-2021-060247)
Supplement: Author's manuscript [file bmjopen-2021-060247.draft_revisions.pdf]

BMJ Open is committed to open peer review. As part of this commitment we make the peer review history of every article we publish publicly available.

When an article is published we post the peer reviewers' comments and the authors' responses online. We also post the versions of the paper that were used during peer review. These are the versions that the peer review comments apply to.

The versions of the paper that follow are the versions that were submitted during the peer review process. They are not the versions of record or the final published versions. They should not be cited or distributed as the published version of this manuscript.

BMJ Open is an open access journal and the full, final, typeset and author-corrected version of record of the manuscript is available on our site with no access controls, subscription charges or pay-per-view fees (http://bmjopen.bmj.com).

If you have any questions on BMJ Open's open peer review process please email info.bmjopen@bmj.com

BMJ Open

Community determinants of COPD exacerbations in elderly patients in Lodz Province, Poland: a retrospective observational Big Data cohort study

Journal:	*BMJ Open*
Manuscript ID	bmjopen-2021-060247
Article Type:	Original research
Date Submitted by the Author:	20-Dec-2021
Complete List of Authors:	Kowalczyk, Anna; Medical University of Lodz, Centre for Family and Community Medicine<br>Kosiek, Katarzyna; Family Doctors' Clinic, Lodz, Poland<br>Godycki-Cwirko , Maciej; Medical University of Lodz, Centre for Family and Community Medicine<br>Zakowska, Izabela; Medical University of Lodz, Centre for Family and Community Medicine
Keywords:	Respiratory infections < THORACIC MEDICINE, PUBLIC HEALTH, PRIMARY CARE

SCHOLARONE™
Manuscripts

I, the Submitting Author has the right to grant and does grant on behalf of all authors of the Work (as defined in the below author licence), an exclusive licence and/or a non-exclusive licence for contributions from authors who are: i) UK Crown employees; ii) where BMJ has agreed a CC-BY licence shall apply, and/or iii) in accordance with the terms applicable for US Federal Government officers or employees acting as part of their official duties; on a worldwide, perpetual, irrevocable, royalty-free basis to BMJ Publishing Group Ltd ("BMJ") its licensees and where the relevant Journal is co-owned by BMJ to the co-owners of the Journal, to publish the Work in this journal and any other BMJ products and to exploit all rights, as set out in our licence.

The Submitting Author accepts and understands that any supply made under these terms is made by BMJ to the Submitting Author unless you are acting as an employee on behalf of your employer or a postgraduate student of an affiliated institution which is paying any applicable article publishing charge ("APC") for Open Access articles. Where the Submitting Author wishes to make the Work available on an Open Access basis (and intends to pay the relevant APC), the terms of reuse of such Open Access shall be governed by a Creative Commons licence – details of these licences and which Creative Commons licence will apply to this Work are set out in our licence referred to above.

Other than as permitted in any relevant BMJ Author's Self Archiving Policies, I confirm this Work has not been accepted for publication elsewhere, is not being considered for publication elsewhere and does not duplicate material already published. I confirm all authors consent to publication of this Work and authorise the granting of this licence.

For peer review only

Title page

Community determinants of COPD exacerbations in elderly patients in Lodz Province, Poland: a retrospective observational Big Data cohort study

Authors:

Anna Kowalczyk[1], Katarzyna Kosiek[2], Maciek Godycki-Cwirko[1], Izabela Zakowska[1]

1. Centre for Family and Community Medicine, the Faculty of Health Sciences, the Medical University of Lodz, Poland
2. Family Doctors' Clinic, Lodz, Poland.

Corresponding author:

Izabela Zakowska

Centre for Family and Community Medicine, Faculty of Medical Sciences,

Medical University of Lodz,

Kopcinskiego 20, 90-153 Lodz, Poland

izabela.zakowska@umed.lodz.pl

tel.: +48 42 679 55 46

Key words: COPD, Big Data, Exacerbations, Primary Health Care, Electronic Health Records.

Word count: 3653

For peer review only

Abbreviations:
AECOPD – Acute Exacerbations of chronic obstructive pulmonary disease
ASC – Ambulatory Specialists Care
ATC - Anatomical-therapeutic-chemical classification
COPD - Chronic Obstructive Pulmonary Disease
ICD-10 - International Classification of Diseases
NFZ - National Health Fund
US - Tax Office (Revenue Administration Regional Office in Lodz)
GUS - National Statistics Center (Statistics Poland)
EHRs - electronic health records
PHC – Primary Health Care
PSM – Propensity Score Matching
SES - socioeconomic status
SOR - Hospital Emergency Department
TERYT - Registry identifiers (GUS)

ABSTRACT

Introduction

The global prevalence of COPD, a progressive disease with exacerbations, is growing worldwide.

Big Data analysis offers an insight into the functioning of the health care system and enables a change in the approach to chronic health problems at individual and population level.

The purpose of this study was to measure the prevalence and identify demographic, economic and environmental local community determinants of COPD exacerbations in elderly using Big Data approach.

Materials and Methods

This was a retrospective observational case-control study in elderly patients with COPD in the Lodz province in Poland based on data from the National Health Found, Tax Office and National Statistics Center databases in 2016. Conditional logistic regression for matched pairs was used to evaluate the local community determinants of COPD exacerbations among COPD patients.

Results

The overall prevalence of COPD in the population of elderly patients registered in PHC clinics in Lodz province in 2016 was 3.65% and the prevalence of exacerbations was 10.35%. The high number of consultations in primary care clinics was associated with higher risk of COPD exacerbations (p=0.0687).

High-income patients were less likely to have exacerbations than low-income patients (high vs low OR = 0.601, 95% CI (0.385-0.939), p = 0.0253). The specialization of the primary care physician did not have an effect on exacerbations (OR = 1.076, 95% CI (0.920-1.257), p=0.3594). Neither the forest cover per gmina (high vs low OR = 0.897, 95% CI (0.605-1.331), p=0.5899; medium vs low OR = 0.925, 95% CI (0.648-1.322), p=0.6699), nor location of gmina (urban vs. urban-rural OR = 1.044; 95% CI (0.673-1.620, p=0.8477), (rural vs. urban-rural OR = 0.897, 95% CI (0.630-1.277), p=0.5470) appears to influence COPD exacerbations (p=0.7934).

Conclusions

Modification of identified local community determinants may potentially decrease the number of exacerbations in COPD elderly patients.

For peer review only

Strengths and limitations

- The study was a pioneering retrospective evaluation of prevalence of COPD including exacerbations in elderly primary care patients in Poland, using three Big Data databases assessing determinants of COPD exacerbations.
- The study included Big Data for about half a million patients aged 65 and over.
- The results can be used to inform decision to better address COPD.
- Its main limitation was the lack of standardization in the method of recording exacerbations between primary care, accident and emergency departments and hospital wards.
- No data existed for some parameters, such as air quality for gaseous and dust pollutants, at the commune level for examined period.

For peer review only

INTRODUCTION

Chronic obstructive pulmonary disease (COPD) is the third leading cause of death worldwide, causing 3.23 million deaths in 2019. [1] Despite the great effort directed at prevention and treatment guidelines, the prevalence continues to increase and is now estimated to range from 4% to 10% worldwide; this value varies considerably across the European union, i.e. from 1.26% to 13.87%, partly because of the differences in the definitions used. [2-4] In Poland, a medium-sized country with population of about 38 million inhabitants, studies suggest a COPD prevalence of 11% in the cites of Warsaw and Lublin; however, data is scarce in other regions. [5]

COPD is a progressive disease characterized by exacerbations, i.e. an acute worsening of the respiratory symptoms which may lead to hospitalization [6-8]. In addition, patients with stable COPD often have acute exacerbations (AECOPD) due to risk factors, comorbidities, poor

treatment adherence and insufficient treatment. [9] Frequent exacerbations, defined as at least two episodes per year, are considered a marker of increased burden and reduced survival in clinical epidemiology studies. [10]

Before such exacerbations can be effectively prevented, there is a need to identify the factors contributing to them. To date, most studies have focused on factors specific to COPD patients themselves, rather than on external factors. [11, 12] However, it is possible that Socioeconomic status (SES) may play a role. SES is a complex assessment of a person's economic and social position in society based on several factors including income, occupation, home and neighborhood environment, and educational attainment. [13]

COPD appears to have a stronger socioeconomic element than other common causes of morbidity and death, including cardiac disease. [13] Also, low income appears to have a greater influence on the prevalence of COPD than asthma. [14] In Canada, where the overall prevalence of COPD was 5.69%, this prevalence was found to be significantly higher among older patients, and those who are currently smoking, underweight or obese, and those with total personal income <$ 20,000 and some post-secondary education. [15]

A number of environmental factors can have effects on health, both positive and negative..

Green spaces, parks and community gardens support community health by reducing stress, promoting physical activity, and improving perceived general health. [16] These measures can be identified and modified to promote positive health behaviors and avoid negative ones, [17] and to shape specific promotional and educational initiatives aimed at reducing the prevalence of COPD and its exacerbations.

Recently, *Big Data* analyses have made it possible to measure, analyze, and document the health of individuals and populations, with the aim of improving public health and enhancing decision making at the individual and community levels. [18] Such applications have been used to monitor the quality of medical and healthcare facilities, improve patient service, detect

For peer review only

disease spread earlier, provide new insights into disease mechanisms through risk factors, and improve treatments. [19, 20]

Earlier studies on COPD exacerbations have been mostly prospective and have involved patients from secondary and/or tertiary care. In contrast, the present study examines associations between well-known patient-level risk factors and triggers of exacerbations of COPD, including local community factors.

OBJECTIVES

To estimate the prevalence of COPD in elderly patients in the province of Lodz Poland, and to determine the prevalence of AECOPD in this population.

To identify local community determinants of disease exacerbations among elderly COPD patients.

METHODS

A retrospective observational case-control cross-sectional study was performed among elderly patients with COPD in the Lodz province in Poland. The study was conducted between January 1st and December 31st, 2016.

DATA SOURCES

Data were retrieved from three Big Data databases of the NFZ (National Health Service), US (Tax Office) and GUS (Central Statistical Office).

The NFZ electronic health records (EHRs) database was used to gain information on sociodemographic (age, sex), inpatient and outpatient healthcare utilisation, including costs of hospitalization, and the cost of dispensed medications in the form of ATC (anatomical

For peer review only

therapeutic chemical) codes, as well as other corresponding costs and information on physicians working in primary health care clinics (PHC) in the study area.

The US database provided information on the income of residents aged 65 and over in the province of Lodz.

The GUS database contained variables potentially associated with exacerbations for 177 gminas (local communities) within the Lodz province.

Local community factors potentially associated with COPD exacerbations in the elderly were selected and classified by experts into (1) health factors, (2) socioeconomic factors and (3) community environmental factors at gmina level, according to the Remington and Catlin methodology [21].

PARTICIPANTS

All participants were patients registered in PHC clinics, aged 65 years and older, residents of Lodz province. All were assigned to a particular gmina; it was assumed that the majority of patients aged 65 and over choose a healthcare clinic close to their place of residence. COPD was indicated by the presence of the International Classification of Diseases (ICD-10) code J44 in patients' medical records; exacerbations were defined as cases "hospitalized with the J44 code as a main reason for admission".

Patient and Public Involvement

This was a Big Data study using patients' anonymized electronic health records without patient involvement.

Big Data Quality process

For peer review only

The Big Data quality process was carried out according to the SAS Data Management Methodology. After cleaning the data, the rules and alerts for monitoring were defined. (details given in APPENDIX)

STATISTICAL ANALYSIS

The data was analysed as described previously in protocol. [22] Additionally, the propensity score matching (PSM) technique and multivariate conditional logistic regression for matched pairs were applied to estimate the local community determinants of AECOPD. [23]

Descriptive statistics were calculated for the total cohort of COPD patients aged 65 and older before and after propensity score matching (PSM). Missing data was excluded from the analysis. The relationships between tested variables and exacerbations (1-with, 0-without) were determined using univariate high-performance logistic regression models, and univariate logistic regression models applied and stratified logistic regression (a single model is for each line). Continuous variables were categorized by quartile. Pearson's correlation coefficient was estimated. Patients with exacerbations were matched retrospectively for gender and age to patients without exacerbations to ensure 1:1 group ratio without repetition using propensity score matching (PSM). Patients who died were excluded from the analysis.

After adjusting for potential covariates, the odds ratio (OR) and 95% confidence intervals 95% (C.I.) of exacerbations between cases and control patients were calculated by multivariate conditional logistic regression for matched pairs models.

Statistically significant findings were assumed as p-value <0.05.

The analysis was performed with the SAS V.9.4 statistical package, MLwiN V.2.24 and STATISTICA V.13.1.

Study size/Power Calculation

For peer review only

The power was calculated for paired t-test for mean difference. Taking number of pairs 1492, calculated mean difference between number of consultations in PHC clinic for patients with exacerbation and without exacerbations was 1.214 (SD=9.658). The standard deviation for the number of consultations in PHC clinic in group with exacerbation was 7.62 and in group without exacerbation was 6.76 , the correlation between them was 0.103 ($p<0.0001$), which gave the power of 99.8%. Total of 499 pairs would allow to 80% power.

RESULTS

Prevalence

The Lodz province comprised 2 485 323 inhabitants as of December 31, 2016 [24]. According to the NFZ electronic health records (EHRs) database within the Lodz province, 472 314 elderly patients were registered in PHC clinics with contracts with the National Health Fund (19% of total number of residents).

Prevalence of COPD in the population of patients registered in PHC clinics in Lodz province in 2016 was 3.65% (17 240 patients coded as J44) of all elderly patients (n= 472 314). Among all COPD patients, 1 784 (10.35%) were recorded as having an exacerbation; this group represents 0.38% of the entire elderly patient population in the Lodz province. During the study, 1 141 of 17 240 (6.62%) patients died and their data was excluded from further analysis. In total, 16 099 COPD patients were included in the analysis.

Characteristics of the COPD patient groups (raw data before PSM)

The group characteristic is presented in Supplementary table 1 and APPENDIX.

For peer review only

Supplementary 1: Table 1 Descriptive characteristics and univariate analysis of COPD exacerbations in cohort of COPD elderly patients and prevalence of COPD in PHC (Lodz province in 2016). (Raw data before PSM).

In total, 1 492 (9.27%) of 16 099 COPD patients reported exacerbations. The number of exacerbations per individual COPD patient ranged from one to six. Most of these patients reported one exacerbation (1 294 patients; 86.73%), with fewer reporting two (148 patients; 9.92%), three (32 patients; 2.14%), four (11 patients; 0.74%); five (five patients; 0.34%) and six exacerbations (two patients; 0.13%).

A statistically significant relationship was observed between sex and exacerbations ($p < 0.0001$). 59.38% of patients in group with exacerbation was male. In addition, a significant relationship was noted between age and exacerbations ($p<0.0001$): the COPD group with exacerbations had a higher median age 75 years old (Lower Quartile= 69, Upper Quartile =81) than the COPD patients without exacerbations 73 years old (q1=68, q3=80).

The number of COPD consultations in PHC clinics, classed as the number of health care consultations with the main diagnosis J44, ranged from 0 to 24 per patient during the study year, with the most common value being one consultation in PHC clinic per COPD 65 patient. Among the people with exacerbation 36.73% did not have any COPD-related consultations in the PHC clinic and 1.14% in the group without exacerbation. 19.17% patients in group with exacerbations had received one COPD consultation (52.72% in group without exacerbation), 12.8% two consultations (20.06% in group without exacerbation), 7.91% three consultations (10.36% in group without exacerbation), 23.39% four and more COPD consultations (15.72% in group without exacerbation).

The number of COPD consultations in 2016 was associated with the number of exacerbations ($p<0.0001$).

For peer review only

The number of consultations for any reason in PHC clinic per 65 +, COPD patient during the study year ranged from 0 to 78, with the most common value being 10 consultations.

The number of consultations in PHC in 2016 were significantly associated with disease exacerbation (medium vs. low p = 0.0350 and high vs. low p = 0.0092). Among patients with exacerbation, about 46% had 12 or more consultations, 44% had a medium number of consultations (n=5 to 12), and only 10% had low number (n=0 to 4).

In total, 2 465 physicians were working in PHC clinics within the Lodz province, including 627 (25.44%) family doctors, and 1 838 (74.56%) other physicians (general internal medicine, pediatrics, physicians without specialization). Of the total physicians, 1 674 (67.91%) were female. The median age of physicians was 52 (min. 25, max. 88) years old.

Similar percentages of patients whose physician was a family doctor vs. other were observed in the group of COPD patient with exacerbations (39.34%) and in the group without exacerbations (37.54%) (p=0.1703).

There was an association between the numbers of packages of reimbursed drugs and exacerbations (R03 AC, AL, BA, BB, DA: p = 0.0001, AK p = 0.0015, R03DC p= 0.4080), although this variables had n=1906 (11.84%) of missing data and need to be carefully interpreted.

The COPD group with exacerbations demonstrated higher numbers of packages of reimbursed drugs with ATC codes R03 AC, AL, BA, BB, DA AK except R03DC compared to those without exacerbations, based on prescriptions provided to the patient.

The NFZ drug reimbursement costs per patient were significantly associated with exacerbations (p<0.0001, n=1906 (11.84%) of missing data).

The total NFZ costs per patient (as sum of costs of PHC, ASC, hospitalization, drug reimbursement and other) was significantly associated with exacerbations (p <0.0001).

Income was also significantly associated with exacerbations (p <0.0001).

For peer review only

The group with exacerbations demonstrated lower income, by PLN 584.02 per year, compared to the group without exacerbations.

Forest cover of the gmina where the patient lived had a significant impact on the exacerbations (high vs. low p=0.0014, medium vs. low p=0<0.0001). 43% of patients in group with exacerbations were residents of gminas with a lower level of forest cover.

COPD exacerbations were found to be significantly associated with patient's place of residence in gmina (urban, rural, urban-rural).

Characteristics of the COPD patient groups (after PSM)

Patients with COPD exacerbations throughout the one-year study period were matched for gender and age at baseline with patients without COPD exacerbations in a 1:1 ratio: n=1 492 cases (50%) and n=1 492 controls (50%).

The characteristics of COPD patients with and without exacerbations after PSM are given in Table 2 and APPENDIX.

Table 2. Descriptive characteristics and local determinants univariate analysis of COPD exacerbations in cohort of COPD elderly patients (Lodz province, 2016; case-control). After PSM.

PSM analysis identified the similar relationships between variables and exacerbations as in the raw data. The median age was 75 (q1=69, q3=81) years for J44 with exacerbations and 75 (q1=69, q3=81) for J44 without exacerbations.

Patients' consultations in PHC clinics, and COPD consultations in PHC clinic were statistically associated with exacerbations (high vs. low p = 0.0687 border of significance, p < 0.0001, respectively).

For peer review only

High number of consultations in PHC clinics (any reason) had 46.18% of patients in group with exacerbations and 37.40% in group without exacerbations.

In addition, 36.73% patients with exacerbations and 0.87% of patients without exacerbations had not received any COPD consultations in PHC clinic. When the number of consultations in PHC with the main diagnosis J44 increased from one to three, a decrease in the number of exacerbations was observed.

There was an association between the numbers of packages of reimbursed drugs and exacerbations (R03 AC, AL, BA, BB, DA: p = 0.0001, AK p = 0. 0.0078, R03DC p= 0. 0.9022. Patients with exacerbations used a greater number of prescriptions for reimbursed drug packaging The patients with exacerbations realized higher numbers of prescriptions of reimbursed drugs from ATC groups with codes R03AC, AL, BA, BB, DA, AK compared to those without exacerbations. Again the numbers of R03DC packages did not differ significantly between the groups.

Significant association was found between total NFZ cost per patient (as the sum of costs of PHC, ASC, hospitalization, drugs reinvestment and other) and COPD exacerbation (p = <0.0001).

Of all patients in the exacerbations group, 37.53% had high total NFZ cost per patient, and 58.65% had medium. Among those without exacerbations, the costs were high in only 12%, medium in 41%, and 46% had low costs.

A significant association was found between income and disease exacerbations (high vs. low p= 0.0439). There were fewer exacerbations in the group of patients with high income.

There were no association between the forest cover of the patient's place of residence in gmina and exacerbations (high vs. low p=0.5082, medium vs. low p=0.6986).

Model of local determinants of COPD exacerbations for elderly COPD patients

For peer review only

Table 2 shows local determinants of COPD exacerbations for 65+ patients.

The final model includes five independent variables: number of consultations in PHC, income, physicians' specialization in PHC, the area covered with forest in gmina of patient's residence and location of gmina (urban, rural, urban-rural).

Among them, two variables turned out to be significant: number of consultations in PHC (border significance) and income.

Patients with a medium number of consultations in PHC demonstrated insignificantly lower chance of exacerbations than those with a low number of consultations (medium <5-12> vs low (<5) OR=0.823, 95% C.I. (0.643-1.053), p=0.1207) (table 2).

Those COPD patients with high number of consultations (above 12) in PHC had 1.26 the odds of having exacerbations relative to those with low consultation number (less than 5), but this was of borderline significance (high (>12) vs low (<5): OR=1.261, 95% C.I. (0.974-1.633), p=0.0784).

COPD patients living in areas with a high income had a lower chance of exacerbations than those in areas with a low income (high vs low OR = 0.601 (0.385-0.939), p = 0.0253).

Type of physician's specialization in PHC had no influence on the exacerbations (family doctor vs other OR = 1.076, 95% C.I. (0.920-1.257, p=0.3594) neither the forest cover area (high vs low OR=0.897, 95% C.I. (0.605-1.331), p = 0.5899, medium vs low OR=0.925, 95% C.I. (0.648-1.322), p=0.6699) or patient's place of residence in gmina (urban vs. urban-rural OR=0.897, 95% C.I. (0.630-1.277), p=0.8477, rural vs. urban-rural, p=0.5470).

DISCUSSION

For peer review only

Despite significant advances in the diagnosis and treatment of COPD, its incidence remains high. In addition, in some cases, the chance of exacerbation can be increased as a result of local risk factors.

Fortunately, nowadays information on community health and health care system can be easily gathered from a number of large databases, as Big Data, and analyzed statistically. [25]

Big Data technology can be used to improve the course of chronic disease and its exacerbations. For example, Korean researchers were able to precisely match COPD exacerbations with external factors. [26, 27]

Similarly, the present study applied Big Data analysis to a large primary care cohort of 472 314 patients aged 65 years and older in the Lodz province, Poland. The results yielded new information on the disease itself and its relationship with local community factors (demographic factors, healthcare use, economic and environmental factors).

A meta-analysis of COPD prevalence reported a pooled prevalence of 9.8% among men and 5.6% among women. [28]

In present study, this value was 3.65% among all elderly patients. COPD exacerbations, hospitalizations and deaths in women have been increasing in several countries, with more women being hospitalized for COPD in recent years [29]; indeed, the percentage of hospitalizations among women in Puglia, Italy was found to increase from 27.8% in 2001 to 35.6% in 2011. [30]

However that tendency was not found in our study where 59.38 % of total COPD patients with exacerbation were male (raw data $p<0.0001$).

More research is needed to verify these apparent gender differences, not only among hospitalized patients but also in their daily environment.

In a Danish study, the risk of hospitalization for COPD increased with age until ~80 years for females and 85 years for males. [31]

For peer review only

Our findings also indicate a significant relationship between age and exacerbation (raw data $p < 0.0001$). Of those with exacerbations, 47% are aged 65-75 years, 40% are aged 75-85 years, and approximately 14% are 85 years of age or older. In our study, the age of patients in the exacerbation group was higher than in group without exacerbation: 75 years old vs. 74 (raw data $p<0.0001$)

The high number of primary care consultations per patient and high income in the local area were also significant determinants of exacerbations among COPD patients aged 65 and over ($p=0.0784$ border of significance and $p=0.0253$, respectively), controlled for the specialization of the physician in the PHC, forest cover of the local area and the location of the gmina.

In the present study, patients with a higher number of consultations have more than 1.26 times the chances of exacerbations compared to patients with a low number of consultations, but this was on the border of statistical significance (OR= 1.261; 95% C.I.=0.974, 1.633; $p=0.0784$). It is likely that the former group have a greater chance of referral to a hospital by a primary care physician, and thus faster diagnosis and treatment.

Patients with high income are less likely to have COPD exacerbations than those with low income (OR= 0.601; 95% C.I. 0.385, 0.939; $p=0.0253$). Other studies have reported similar trends. [32] This may be related to decreased access to health care services among lower income groups. Such problems with access may delay the diagnosis of COPD, leading to a poorer prognosis and increased morbidity and mortality. [33] Addressing this issue may reduce the workload of the healthcare system and allow more targeted use of available resources.

The association between neighbourhood greenness and respiratory outcomes in adults appears ambiguous. [34] One cohort study including 1.3 million adults in Canada found that greater neighbourhood greenness could reduce the risk of respiratory mortality [35]; however, a Chinese cohort study concluded that neighborhood greenness might in fact be a risk factor. [36]

In our present study, forest cover per gmina was not found to have any statistically significant effect on the COPD exacerbation.

Rural residence has been found to be associated with an increased incidence of COPD exacerbations, although not severe ones. [37] In countries with large rural populations, COPD is more prevalent in rural than urban areas. This disparity may be related to increased agricultural exposure (wheat, grain, cotton dust, pesticides), among others. [38] In addition, studies suggest this may be associated with increased indoor air exposure associated with burning biomass fuels. [39]

In our study, patient's place of residence in gmina (urban, rural, urban-rural) did not appear to have any influence on the COPD exacerbations.

In addition, the specialization of the physician in primary care did not have any effect on the COPD exacerbations.

CONCLUSIONS

This study investigates the determinants for AECOPD with the aim of improving prevention and treatment. It was made possible by the use of a panel of experts from various fields: primary care physicians, public health specialists, a Big Data analyst and data managers in public institutions (NFZ, GUS and US). Our findings regarding COPD in the Lodz province, Poland may be used for formulating health policy recommendations by the National Health Fund (NFZ) and local government. The Big Data methodological approach used herein can be applied to other chronic diseases. However, further study is needed to determine the role of local medical and environmental factors in improving the health of COPD patients with exacerbations.

ACKNOWLEDGMENTS

For peer review only

The authors specially thank Edward Lowczowski for English language assistance.

Funding Statement

This work was supported by Narodowe Centrum Nauki (National Science Centre, Poland) grant number 2016/21/B/NZ7/02052.

Competing interests statement.

No other relationships or activities that could appear to have influenced the submitted work.

Data sharing

No additional data available.

Research Ethics Approval

The study protocol has been approved by the Bioethical Committee of Medical University of Lodz (RNN/248/18/KE, 10 July 2018).

This Big Data study using patients' anonymized electronic health records data.

Author's contribution

The study design was conceptualized by A.K., M.G.-C K.K. and I.Z. Analyses were performed by I.Z. K.K. and I.Z. prepared the first draft of the manuscript. All authors have read and agreed to the published version of the manuscript.

Table 2: Descriptive characteristics and local determinants of COPD exacerbations in cohort of COPD elderly patients (Lodz province, 2016; case-control). After PSM.

	**Unadjusted****					**Adjusted Logistic Models****	
**Patient Characteristic (After PSM)**	**COPD patients without exacerbations Control (n=1,492)**	**COPD patients with exacerbations Cases (n=1492)**	**Total (n=2,984)**	**OR (95% CI)****	**P-value **Univariate Conditional Logistic Regression Matched Pairs analysis.**	**OR (95% CI)**	**P - value**
**Patient level**							
**Demographic**							
**COPD Patients (Sum)(%) Total**	1492 (50)	1492 (50)	2984 (100)				
**Sex**							
**Male**	886 (59.38)	886 (59.38)	1772 (59.38)	After PSM variable dependent on the strata (No Effect)	NS	After PSM variable dependent on the strata (No Effect)	NS
**Female**	606 (40.62)	606 (40.62)	1212 (40.62)				
**Age (years)**							
**<75-85)**	600(40.21)	600(40.21)	1200(40.21)	After PSM variable dependent on the strata (No Effect)	NS	After PSM variable dependent on the strata (No Effect)	NS
**<85+>**	175(11.73)	175(11.73)	350(11.73)				
**<65-75)**	717(48.06)	717(48.06)	1434(48.06)				
**Patient's place of residence in gmina (localization)**							
**1-urban vs. 3**	862 (57.77)	864 (57.91)	1726 (57.84)	1.028 (0.848-1.246)	0.7761	1.044 (0.673-1.620)	0.8477
**2-rural vs. 3**	353 (23.66)	358 (23.99)	711 (23.83)	1.04 (0.833-1.3)	0.7274	0.897 (0.630-1.277)	0.5470
**3-urban-rural (Ref. Cat.)**	277 (18.57)	270 (18.1)	547 (18.33)	1 Ref. Cat.		1 Ref. Cat.	
**Healthcare uses**							
**Specialization of physician working in PHC**							
**Family doctors vs. Other**	562(37.67)	587(39.34)	1149(38.51)	1.080(0.926-1.259)	0.328	1.076 (0.920-1.257)	0.3594

For peer review only

**Other (Ref. Cat.)**	930(62.33)	905(60.66)	1835(61.49)	1 Ref. Cat.		1 Ref. Cat.	
**Number of consultations in PHC^**							
**high (>12) vs. low (<5)**	558 (37.4)	689 (46.18)	1247 (41.79)	1.269 (0.982-1.639)	0.0687	1.261 (0.974-1.633)	0.0784
**medium <5-12> vs. low (<5)**	783 (52.48)	649 (43.5)	1432 (47.99)	0.831 (0.651-1.061)	0.1384	0.823 (0.643-1.053)	0.1207
**low (<5) (Ref. Cat.)**	151 (10.12)	154 (10.32)	305 (10.22)	1 Ref. Cat.		1 Ref. Cat.	
**Number of COPD consultations in PHC^**							
**0 vs. 4+**	13 (0.87)	548 (36.73)	561 (18.8)	41.078 (18.064-93.413)	<0.0001		
**1 vs. 4+**	751 (50.34)	286 (19.17)	1037 (34.75)	0.288 (0.224-0.37)	<0.0001		
**2 vs. 4+**	299 (20.04)	191 (12.8)	490 (16.42)	0.488 (0.369-0.645)	<0.0001		
**3 vs. 4+**	185 (12.4)	118 (7.91)	303 (10.15)	0.528 (0.377-0.738)	0.0002		
**4+ (Ref. Cat.)**	244 (16.35)	349 (23.39)	593 (19.87)	Ref. Cat.			
**Gmina/Commune, post-codes level**							
**Socioeconomic Status**							
**Total personal income of residents per PHC post-codes (Tax Office)**							
**high (>21056,52) vs. low (<17079,50)**	371 (24.87)	345 (23.12)	716 (23.99)	0.696 (0.489-0.990)	0.0439	0.601 (0.385-0.939)	0.0253
**medium <17079,50-21056,52> vs. low (<17079,50)**	759 (50.87)	768 (51.47)	1527 (51.17)	0.910 (0.722-1.147)	0.4257	0.824 (0.581-1.169)	0.2784
**low (<17079,50) (Ref. Cat.)**	362 (24.26)	379 (25.4)	741 (24.83)	1 Ref. Cat.		1 Ref. Cat.	
**Environmental factors**							
**Forest cover of the patient's place of residence in gmina (GUS)**							
**high (>20,60%) vs. low (<7,00%)**	348 (23.32)	340 (22.79)	688 (23.06)	0.896 (0.648-1.24)	0.5082	0.897 (0.605-1.331)	0.5899
**medium <7,00%-20,60%> vs. low (<7,00%)**	776 (52.01)	776 (52.01)	1552 (52.01)	0.94 (0.688-1.285)	0.6986	0.925 (0.648-1.322)	0.6699
**low (<7,00%) (ref. Cat.)**	368 (24.66)	376 (25.2)	744 (24.93)	1 Ref. Cat.		1 Ref. Cat.	
**Total**	1492 (50)	1492 (50)	2984 (100)				

CI=Confidence Interval, OR=Odds Ratio. #=Odds Ratio Estimates (Point Estimate). 95% Wald Confidence Limits. OR (95% CI).
OR with 95% Wald CI= Odds Ratios with 95% Wald Confidence Limits. CI=Confidence interval, OR=Odds ratio, Ref. cat.=reference category. #The LOGISTIC Procedure, Conditional Logistic Regression for Matched Pairs.

For peer review only

References

1. WHO. Newsroom. The top 10 causes of death. WHO's Global Health Estimates2021 05.11.2021]: Available from: https://www.who.int/news-room/fact-sheets/detail/the-top-10-causes-of-death.
2. Adeloye D, Chua S, Lee C, Basquill C, Papana A, Theodoratou E, et al. Global and regional estimates of COPD prevalence: Systematic review and meta-analysis. Journal of global health. [Meta-Analysis
Review
Systematic Review]. 2015 Dec;5(2):020415.
3. Boven Jv, Gaughan J, Soriano J, Correia de Sousa J, Baxter N, Román-Rodríguez M, et al. P139 The burden of copd across the european union: development of the european copd atlas. Thorax. 2016;71(Suppl 3):A158-A.
4. Gibson GJ, Loddenkemper R, Lundback B, Sibille Y. Respiratory health and disease in Europe: the new European Lung White Book. Eur Respir J. [Editorial]. 2013 Sep;42(3):559-63.
5. Blanco I, Diego I, Bueno P, Fernandez E, Casas-Maldonado F, Esquinas C, et al. Geographical distribution of COPD prevalence in Europe, estimated by an inverse distance weighting interpolation technique. International journal of chronic obstructive pulmonary disease. 2018;13:57-67.
6. Mullerova H, Shukla A, Hawkins A, Quint J. Risk factors for acute exacerbations of COPD in a primary care population: a retrospective observational cohort study. BMJ open. [Research Support, Non-U.S. Gov't]. 2014 Dec 18;4(12):e006171.
7. Perera PN, Armstrong EP, Sherrill DL, Skrepnek GH. Acute exacerbations of COPD in the United States: inpatient burden and predictors of costs and mortality. COPD. 2012 Apr;9(2):131-41.
8. Qureshi H, Sharafkhaneh A, Hanania NA. Chronic obstructive pulmonary disease exacerbations: latest evidence and clinical implications. Therapeutic advances in chronic disease. [Review]. 2014 Sep;5(5):212-27.
9. Esteban C, Quintana JM, Moraza J, Aburto M, Egurrola M, Espana PP, et al. Impact of hospitalisations for exacerbations of COPD on health-related quality of life. Respiratory medicine. [Research Support, Non-U.S. Gov't]. 2009 Aug;103(8):1201-8.
10. Hurst JR, Vestbo J, Anzueto A, Locantore N, Mullerova H, Tal-Singer R, et al. Susceptibility to exacerbation in chronic obstructive pulmonary disease. The New England journal of medicine. [Research Support, Non-U.S. Gov't]. 2010 Sep 16;363(12):1128-38.
11. Husebo GR, Bakke PS, Aanerud M, Hardie JA, Ueland T, Gronseth R, et al. Predictors of exacerbations in chronic obstructive pulmonary disease--results from the Bergen COPD cohort study. PLoS One. 2014;9(10):e109721.
12. Kerkhof M, Freeman D, Jones R, Chisholm A, Price DB, Grp RE. Predicting frequent COPD exacerbations using primary care data. International journal of chronic obstructive pulmonary disease. 2015;10:2439-50.
13. Steenland K, Hu S, Walker J. All-cause and cause-specific mortality by socioeconomic status among employed persons in 27 US states, 1984-4997. American Journal of Public Health. 2004 Jun;94(6):1037-42.
14. Pleasants RA, Ohar JA, Croft JB, Liu Y, Kraft M, Mannino DM, et al. Chronic Obstructive Pulmonary Disease and Asthma - Patient Characteristics and Health Impairment. Copd-Journal of Chronic Obstructive Pulmonary Disease. 2014 Jun;11(3):256-66.
15. Osman S, Ziegler C, Gibson R, Mahmood R, Moraros J. The Association between Risk Factors and Chronic Obstructive Pulmonary Disease in Canada: A Cross-sectional Study

For peer review only

Using the 2014 Canadian Community Health Survey. International Journal of Preventive Medicine. 2017 Oct;8.
16. Maas J, Verheij RA, Groenewegen PP, de Vries S, Spreeuwenberg P. Green space, urbanity, and health: how strong is the relation? J Epidemiol Community Health. [Research Support, Non-U.S. Gov't]. 2006 Jul;60(7):587-92.
17. Jakubowski B, Frumkin H. Environmental Metrics for Community Health Improvement. Preventing Chronic Disease. 2010 Jul;7(4).
18. Galea S, Abdalla SM, Sturchio JL. Social determinants of health, data science, and decision-making: Forging a transdisciplinary synthesis. Plos Medicine. [Research Support, Non-U.S. Gov't]. 2020 Jun;17(6):e1003174.
19. Dinov ID, Heavner B, Tang M, Glusman G, Chard K, Darcy M, et al. Predictive Big Data Analytics: A Study of Parkinson's Disease Using Large, Complex, Heterogeneous, Incongruent, Multi-Source and Incomplete Observations. PLoS One. 2016;11(8):e0157077.
20. Tan SS, Gao G, Koch S. Big Data and Analytics in Healthcare. Methods Inf Med. [Editorial
Introductory]. 2015;54(6):546-7.
21. Remington PL, Catlin BB, Gennuso KP. The County Health Rankings: rationale and methods. Popul Health Metr. 2015;13:11.
22. Zakowska I, Kosiek K, Kowalczyk A, Grabowski J, Godycki-Cwirko M. Community determinants of COPD exacerbations in elderly patients in Poland: protocol for a retrospective Big Data observational cohort study. BMJ open. [Research Support, Non-U.S. Gov't]. 2019 Jun 22;9(6):e030524.
23. Morgan AD, Herrett E, De Stavola BL, Smeeth L, Quint JK. COPD disease severity and the risk of venous thromboembolic events: a matched case-control study. Int J Chron Obstruct Pulmon Dis. 2016;11:899-908.
24. GUS. Łódzkie Voivodship 2017 - Subregions, Powiats, Gminas. Statistical Office in Łódź. GUS. 2017(Statistical Yearbook of Łódzkie Voivodship 2017.).
25. Wamba SF, Akter S, Edwards A, Chopin G, Gnanzou D. How 'big data' can make big impact: Findings from a systematic review and a longitudinal case study. International Journal of Production Economics. 2015 Jul;165:234-46.
26. Lee J, Jung HM, Kim SK, Yoo KH, Jung KS, Lee SH, et al. Factors associated with chronic obstructive pulmonary disease exacerbation, based on big data analysis. Scientific reports. [Research Support, Non-U.S. Gov't]. 2019 Apr 30;9(1):6679.
27. Rhee CK, Kim JW, Yoo KH, Jung KS. Prediction model of COPD acute exacerbation with big data by machine learning methods. European Respiratory Journal. 2020 Sep 7;56.
28. Halbert RJ, Natoli JL, Gano A, Badamgarav E, Buist AS, Mannino DM. Global burden of COPD: systematic review and meta-analysis. Eur Respir J. [Meta-Analysis
Research Support, Non-U.S. Gov't
Review
Systematic Review]. 2006 Sep;28(3):523-32.
29. Han MK, Postma D, Mannino DM, Giardino ND, Buist S, Curtis JL, et al. Gender and chronic obstructive pulmonary disease: why it matters. Am J Respir Crit Care Med. [Editorial
Research Support, N.I.H., Extramural
Research Support, U.S. Gov't, Non-P.H.S.]. 2007 Dec 15;176(12):1179-84.
30. Moretti AM, Gallone MS, Parisi D, Tafuri S, Germinario C. Gender differences and hospitalization for COPD: an analysis of data from Puglia (Italy). Ital J Gender-Specific Med. 2015;Vol. 1, N. 2:66-72.
31. Lykkegaard J, dePont Christensen R, Davidsen JR, Stovring H, Andersen M, Sondergaard J. Trends in the lifetime risk of COPD exacerbation requiring hospitalisation. Eur Respir J. [Research Support, Non-U.S. Gov't]. 2013 Oct;42(4):964-71.

For peer review only

32. Eisner MD, Blanc PD, Omachi TA, Yelin EH, Sidney S, Katz PP, et al. Socioeconomic status, race and COPD health outcomes. J Epidemiol Community Health. [Research Support, N.I.H., Extramural]. 2011 Jan;65(1):26-34.
33. van Doorslaer E, Masseria C, Koolman X. Inequalities in access to medical care by income in developed countries. Cmaj. [Research Support, Non-U.S. Gov't]. 2006 Jan 17;174(2):177-83.
34. Fong KC, Hart JE, James P. A Review of Epidemiologic Studies on Greenness and Health: Updated Literature Through 2017. Curr Env Hlth Rep. 2018 Mar;5(1):77-87.
35. Crouse DL, Pinault L, Balram A, Hystad P, Peters PA, Chen H, et al. Urban greenness and mortality in Canada's largest cities: a national cohort study. Lancet Planet Health. 2017 Oct;1(7):e289-e97.
36. Fan J, Guo Y, Cao Z, Cong S, Wang N, Lin H, et al. Neighborhood greenness associated with chronic obstructive pulmonary disease: A nationwide cross-sectional study in China. Environ Int. [Research Support, Non-U.S. Gov't]. 2020 Nov;144:106042.
37. Burkes RM, Gassett AJ, Ceppe AS, Anderson W, O'Neal WK, Woodruff PG, et al. Rural Residence and Chronic Obstructive Pulmonary Disease Exacerbations. Analysis of the SPIROMICS Cohort. Ann Am Thorac Soc. 2018 Jul;15(7):808-16.
38. Hu G, Zhou Y, Tian J, Yao W, Li J, Li B, et al. Risk of COPD from exposure to biomass smoke: a metaanalysis. Chest. [Meta-Analysis
Review]. 2010 Jul;138(1):20-31.
39. Perez-Padilla R, Regalado J, Vedal S, Pare P, Chapela R, Sansores R, et al. Exposure to biomass smoke and chronic airway disease in Mexican women. A case-control study. Am J Respir Crit Care Med. [Research Support, Non-U.S. Gov't]. 1996 Sep;154(3 Pt 1):701-6.

For peer review only

Supplementary 1: **Table 1** Descriptive characteristics and univariate analysis of COPD exacerbations in cohort of COPD elderly patients and prevalence of COPD in PHC (Lodz province in 2016). (Raw data before PSM).

**Patient Characteristic (Before PSM)**	**COPD patients without exacerbations (n=14607)**	**COPD patients with exacerbations (n=1492)**	**Total (n=16099)**	**OR (95% CI), p-value #Univariate Logistic Regression.**	**P-value ##Univariate High-Performance Logistic Regression.**
Patient level					
Demographic					
COPD Patients (Sum)(%) Total	14607 (90.73)	1492 (9.27)	16099(100)		
Sex					
Male	7652(52.39)	886(59.38)	8538(53.03)	1.329(1.192-1.481) <0.0001	<0.0001
Female	6955(47.61)	606(40.62)	7561(46.97)	1 Ref. Cat.	1 Ref. Cat.
Age (years)					
<75-85)	5046(34.55)	600(40.21)	5646(35.07)	1.322(1.18-1.482) <0.0001	<0,0001
<85+>	1587(10.86)	175(11.73)	1762(10.94)	1.226(1.031-1.459) 0.0214	0.0214
<65-75)	7974(54.59)	717(48.06)	8691(53.98)	1 Ref. Cat.	1 Ref. Cat.
Patient's place of residence in gmina (localization)					
1-urban	8971(61.42)	864(57.91)	9835(61.09)	0.840(0.728-0.971) 0.0179	0.0179
2-rural	3280(22.45)	358(23.99)	3638(22.6)	0.952(0.806-1.125) 0.5661	0.5662
3-urban-rural	2356(16.13)	270(18.1)	2626(16.31)	1 Ref. Cat.	1 Ref. Cat.
Healthcare uses					
Specialization of physician working in PHC					
Family doctors	5483(37.54)	587(39.34)	6070(37.7)	1 Ref. Cat.	1 Ref. Cat.
Other	9124(62.46)	905(60.66)	10029(62.3)	0.926(0.831-1.033) 0.1703	0.1703
Number of consultations in PHC^					
high (>12)	5309(36.35)	689(46.18)	5998(37.26)	1.277(1.063-1.535) 0.0091	0.0092
medium <5-12>	7783(53.28)	649(43.5)	8432(52.38)	0.820(0.682-0.986) 0.0351	0.0350

For peer review only

low (<5)	1515(10.37)	154(10.32)	1669(10.37)	1 Ref. Cat.	
Number of COPD consultations in PHC^					
0	166 (1.14)	548 (36.73)	714 (4.44)	21.718 (17.658-26.712) <0.0001	<0.0001
1	7701 (52.72)	286 (19.17)	7987 (49.61)	0.244 (0.208-0.288) <0.0001	<0.0001
2	2930 (20.06)	191 (12.8)	3121 (19.39)	0.429 (0.357-0.516) <0.0001	<0.0001
3	1514 (10.36)	118 (7.91)	1632 (10.14)	0.513 (0.412-0.638) <0.0001	<0.0001
4+	2296 (15.72)	349 (23.39)	2645 (16.45)	1 Ref. Cat.	1 Ref. Cat.
Gmina/Commune, post-codes level					
Socioeconomic Status					
Total personal income of residents per PHC post-codes (Tax Office)					
high (>21992,62)	3717 (25.45)	240 (16.09)	3957 (24.58)	0.573 (0.486-0.677) <.0001	<0.0001
medium <17612,68-21992,62>	7231 (49.5)	840 (56.3)	8071 (50.13)	1.032 (0.911-1.168) 0.6232	0.6232
zlow (<17612,68)	3659 (25.05)	412 (27.61)	4071 (25.29)	1 Ref. Cat.	1 Ref. Cat.
Environmental factors					
Forest cover of the patient's place of residence in gmina (GUS)					
high (>17,10%)	3043 (20.83)	308 (20.64)	3351 (20.81)	0.791 (0.686-0.913) 0.0014	0.0014
medium <8,40%-17,10%>	6583 (45.07)	547 (36.66)	7130 (44.29)	0.65 (0.576-0.733) <.0001	<0.0001
zlow (<8,40%)	4981 (34.1)	637 (42.69)	5618 (34.9)	1 Ref. Cat.	1 Ref. Cat.
Total	14607 (90.73)	1492 (9.27)	16099 (100)		

^Pearson Correlation Coefficient=0,2468, p<0.0001 between "Number of consultations in PHC" and "Number of COPD consultations in PHC".
CI=Confidence Interval, OR=Odds Ratio. #=Odds Ratio Estimates (Point Estimate). 95% Wald Confidence Limits. OR (95% CI). #Univariate analysis Univariate Logistic Regression. ##Univariate High-Performance Logistic Regression.

For peer review only

APPENDIX

Table 1. Descriptive characteristics of cohort of COPD elderly patients within Lodz province (Raw data before PSM).

Characteristic	COPD without exacerbations (n=14 607)	COPD with exacerbations (n=1 492)	Total (n=16 099)	Odds Ratio Estimates Point Estimate 95% Wald Confidence Limits OR [95% CI] - Univariate analysis# HP Logistic Regression	P-value HP Logistic Regression
**Total J44 Patients Sum (%)**	14 607 (90.73)	1 492 (9.27)	16 099		
**Exacerbation number**	0	1	Total	Data only for the group with exacerbations.	
**1**	0 (0)	1 294 (86.73)	1 294 (8.04)		
**2**	0 (0)	148 (9.92)	148 (0.92)		
**3**	0 (0)	32 (2.14)	32 (0.2)		
**4**	0 (0)	11 (0.74)	11 (0.07)		
**5**	0 (0)	5 (0.34)	5 (0.03)		
**6**	0 (0)	2 (0.13)	2 (0.01)		
**Total**	**14 607 (90.73)**	**1 492 (9.27)**	**16 099 (100)**		
**Exacerbation number**					
**(min. - max.)**	0	(1 - 6)	(1 - 6)		
**Missing**	14 607	0	14 607	Data only for the group with exacerbations.	
**Total (including Missing)**	14 607 (90.73)	1 492 (9.27)	16 099 (100)		

For peer review only - http://bmjopen.bmj.com/site/about/guidelines.xhtml

Age of the person 65+Median(q1 - q3)	73 (68 - 80)	75 (69 - 81)	74 (69 - 80)	1.016 [1.009, 1.023] **Continuous var.	<.0001
Number of COPD consultations (PHC) clinics					
(min. - max.)	(0 - 24)	(0 - 20)	(0 - 24)		
mode	1	0	1		
Number of consultations in PHC (min. - max.) **Continuous var.	(0 - 78)	(0 - 59)	(0 - 78)		
Number of consultations in PHC mode**Continuous var.	7	13	10		
COSTS **Continuous var. N=16 099	Percent of median cost per COPD with exacerbations/median cost per COPD without exacerbations [%]				
Total stationary cost [%]	100.00%			HP LR estimate= 0.000050 **Continuous var.	<.0001
Total cost of reimbursed Drugs [%]	156.49%			HP LR estimate= 0.000226 **Continuous var.	<.0001
TOTAL NFZ cost [%]	244.02%			HP LR estimate= 0.000040 **Continuous var.	<.0001
Drugs Number of packages Avg±SD					
R03AC NMiss=1906	5±5.7	6±6.95	5±5.86	1.036 [1.028,1.044] **Continuous var.	<.0001
R03AK NMiss=1906	2±3.83	2±3.99	2±3.84	1.022 [1.008,1.035] **Continuous var.	0.0015

For peer review only - http://bmjopen.bmj.com/site/about/guidelines.xhtml

**R03AL NMiss=1906**	1±(5.39	3±8.99	1±5.89	1.041 [1.034, 1.048] **Continuous var.	<.0001
**R03BA NMiss=1906**	2±4.51	4±5.72	2±4.66	1.043 [1.034, 1.053] **Continuous var.	<.0001
**R03BB NMiss=1906**	5±6.11	6±7.3	5±6.27	1.037 [1.029, 1.044] **Continuous var.	<.0001
**R03DA NMiss=1906**	3±4.99	4±5.12	3±5.02	1.038 [1.028, 1.048] **Continuous var.	<.0001
**R03DC NMiss=1906**	0±1.68	0±1.52	0±1.67	0.985 [0.952, 1.020] **Continuous var.	0.4080
**COST OF MEDICINES (Drugs) [%]**	Percent of median cost per COPD with exacerbations/median cost per COPD without exacerbations [%]				
**Total Fund Cost (Drugs) in [%]NMiss=1906 (11.84%)**	144.69%			HP LR estimate= 0.000316 **Continuous var.	<.0001
**Income per post code NMiss=99 Difference between median COPD with exacerbations and median without exacerbations [PLN]**	584.02 PLN			**Continuous	<.0001
**Did the patient die in 2016?**					
**N**	14 607 (94.51)	1 492 (83.63)	16 099 (93.38)		
**T**	849 (5.49)	292 (16.37)	1 141 (6.62)		
**Total**	15 456 (89.65)	1 784 (10.35)	17 240 (100)		
**Physicians' characteristics in PHC in Lodz Voivodeship**					
**Physicians' Age**					
**Median(q1-q3)**	52 (43.61)				
**Number**	2 465				

For peer review only - http://bmjopen.bmj.com/site/about/guidelines.xhtml

**Physician's sex**					
**Women=0**	1 674 (67.91)				
**Men=1**	791 (32.09)				
**Total**	2 465 (100)				
**Physician's spacjalization**					
**Other=0**	1 838 (74.56)				
**Family doctor=1**	627 (25.44)				
**Total**	2 465 (100)				
**Total Physicians in Lodz voivodeship****	2 465 (100)				
**Prevalence**					
**Prevalence of total of COPD n=17240 among 65+ n=472 314 in Lodz Voivodeship, in PHC in 2016 (including death) = 3.65%**					
**Prevalence of total of exacerbations n=1784 among COPD 65+ n=17240 in Lodz Voivodeship, in PHC in 2016 (including death)= 10.35%**					
**Patient Characteristic**	**COPD without exacerbations (n=15 456)**	**COPD with exacerbations (n=1 784)**	**Total (n=17 240)**	**OR (95% CI) - Univariate analysis Logistic Regression**	**P-value Univariate HP Logistic Regression**
**Total Patients COPD 65+ including deaths**	15 456 (89.65)	1 784 (10.35)	17 240 (100)		
**Did the patient die in 2016?**					
**No**	14 607 (94.51)	1 492 (83.63)	16 099 (93.38)		
**Yes**	849 (5.49)	292 (16.37)	1 141 (6.62)		
**Total**	15 456 (89.65)	1 784 (10.35)	17 240 (100)		
**Sex**					
**Female**	7 256 (46.95)	708 (39.69)	7 964 (46.19)	F Ref. Cat.	
**Male**	8 200 (53.05)	1 076 (60.31)	9 276 (53.81)	1.345 [1.217,1.486]	<0.0001
**Total**	15 456 (89.65)	1 784 (10.35)	17 240 (100)		

For peer review only - http://bmjopen.bmj.com/site/about/guidelines.xhtml

**Age of the person 65 categories**					
**<75-85) vs <65-75)**	5 375 (34.78)	707 (39.63)	6 082 (35.28)	1.3 [1.169,1.445]	<0.0001
**<85+> vs <65-75)**	1 819 (11.77)	241 (13.51)	2 060 (11.95)	1.309 [1.125,1.524]	0.0005
**<65-75)**	8 262 (53.45)	836 (46.86)	9 098 (52.77)	<65-75) Ref. Cat.	
**Total**	15 456 (89.65)	1 784 (10.35)	17 240 (100)		
**Age of the person 65+, *continuous**					
**Median(q1-q3)**	74 (69-80)	75 (69-81)	74 (69-80)		
**Avg±SD**	74.79±7.29	75.68±7.2	74.88±7.28	1.017 [1.010,1.023]	<.0001

*Data are presented as n (%), mean ±SD or median (q1 - q3)..
#Crude, Univariate Model, OR (95% CI), Odds Ratio with 95% Wald, Confidence Limits using proc logistic, proc hplogistic.
**Lodz Voivodeship known as Lodz Province (Wikipedia): https://en.wikipedia.org/wiki/%C5%81%C3%B3d%C5%BA_Voivodeship

For peer review only - http://bmjopen.bmj.com/site/about/guidelines.xhtml

Table 2 Characteristic of COPD patients with and without exacerbations (After PSM).

Characteristic	COPD patients without exacerbations (n=1 492) Control (J44) n(%)	COPD patients with exacerbations (n=1 492) Case (Hospitalized due to J44) n(%)	Total patients (n=2 984) n(%)	OR (95% Wald Confidence Limits). (Point Estimate.) OR (95% CI) - Univariate analysis# Paired LR	P-value
**Total**	1 492 (50)	1 492 (50)	2 984 (100)		
**Exacerbation number**				Data only for the group with exacerbations.	
**1**	0 (0)	1 294 (86.73)	1 294 (8.04)		
**2**	0 (0)	148 (9.92)	148 (0.92)		
**3**	0 (0)	32 (2.14)	32 (0.2)		
**4**	0 (0)	11 (0.74)	11 (0.07)		
**5**	0 (0)	5 (0.34)	5 (0.03)		
**6**	0 (0)	2 (0.13)	2 (0.01)		
**Total**	1 492 (50)	1 492 (50)	2 984 (100)		
Age of the person 65+ (years) Median(q1-q3)	75 (69.00- 81.00)	75 (69.00- 81.00)	75 (69.00- 81.00)	PSM NS LR No Effect after PSM	NS
**Number of refunded drugs packages (ATC groups) (Missing 7.37%) Avg±SD)**					
**R03AC**	4.65±±5.63	6.08±6.95	5.4±6.39	1.037 [1.023,1.051]	<0.0001
**R03AK**	2.01±3.9	2.46±3.99	2.25±3.95	1.028 [1.007,1.049]	0.0078
**R03AL**	0.95±4.18	3.41±8.99	2.24±7.21	1.087 [1.064,1.11]	<0.0001
**R03BA**	2.38±4.25	3.55±5.72	3±5.1	1.051 [1.033,1.069]	<0.0001
**R03BB**	4.47±5.83	6.38±7.3	5.46±6.71	1.049 [1.035,1.064]	<0.0001
**R03DA**	3.06±5.12	4.01±5.12	3.56±5.14	1.038 [1.022,1.055]	<0.0001
**R03DC**	0.25±1.45	0.28±1.52	0.26±1.49	0.997 [0.945,1.051]	0.9022
**Total cost of the NFZ for the realized PHC declarations from all lists of 2016, all current and settled services in 2016, regardless of the main diagnosis and reimbursement of drugs from prescriptions completed in 2016 (in general the above costs)#.**					
**high (>21 056.52)**	186 (12.47)	560 (37.53)	746 (25)	42.804 [28.145,65.097]	<0.0001
**medium <2 497.62-8 264.65>**	618 (41.42)	875 (58.65)	1 493 (50.03)	18.503 [12.627,27.115]	<0.0001
**low (<2 497.62)**	688 (46.11)	57 (3.82)	745 (24.97)	1 Ref. Cat.	
**Total**	1 492 (50)	1 492 (50)	2 984 (100)		

*Data are presented as n (%), mean ±SD or median (q1q3).
IQR – interquartile range

For peer review only - http://bmjopen.bmj.com/site/about/guidelines.xhtml

Big Data Quality (Big Data Cleansing) Results: for Medical (NFZ) and Business (Tax Office) Data.

Files were obtained from Big Data databases (NFZ, Tax Office) and GUS. In total, data for n = 472,314 cohorts of 65+ patients registered in primary health care (PHC) were obtained from electronic medical records (EHR) of the National Health Fund in the Lodz Voivodeship. In total, n = 439,577 inhabitants 65+ living in the Lodz Voivodeship were obtained from the databases of 28 Tax Offices located in the Lodz Voivodeship. Variables for n = 177 communes of the Lodz Voivodship, which, according to the literature review, were related to exacerbations were collected from the GUS databases. Patient table from NFZ showed that the identifier has no duplicates. "Codes of comorbidities with COPD" had 98.8% of missing data (NFZ), and the variable "ICD9 procedures for other stays" had 98.1% of missing data (NFZ). Only 0.1% was missing in the variable "Postal code for 65+ residents" in Tax Office databases. Data on air quality for the group "Air pollutant emission from particular nuisance plants (G216)" consisting of 6 subgroups were not available at the level of data sharing for gmina level for 2016 in the GUS database (accessed on November 3, 2021) .

Propensity score matching (PSM) - before and after - overlay histogram_ age, gender

Histograms for the age before and after PSM.

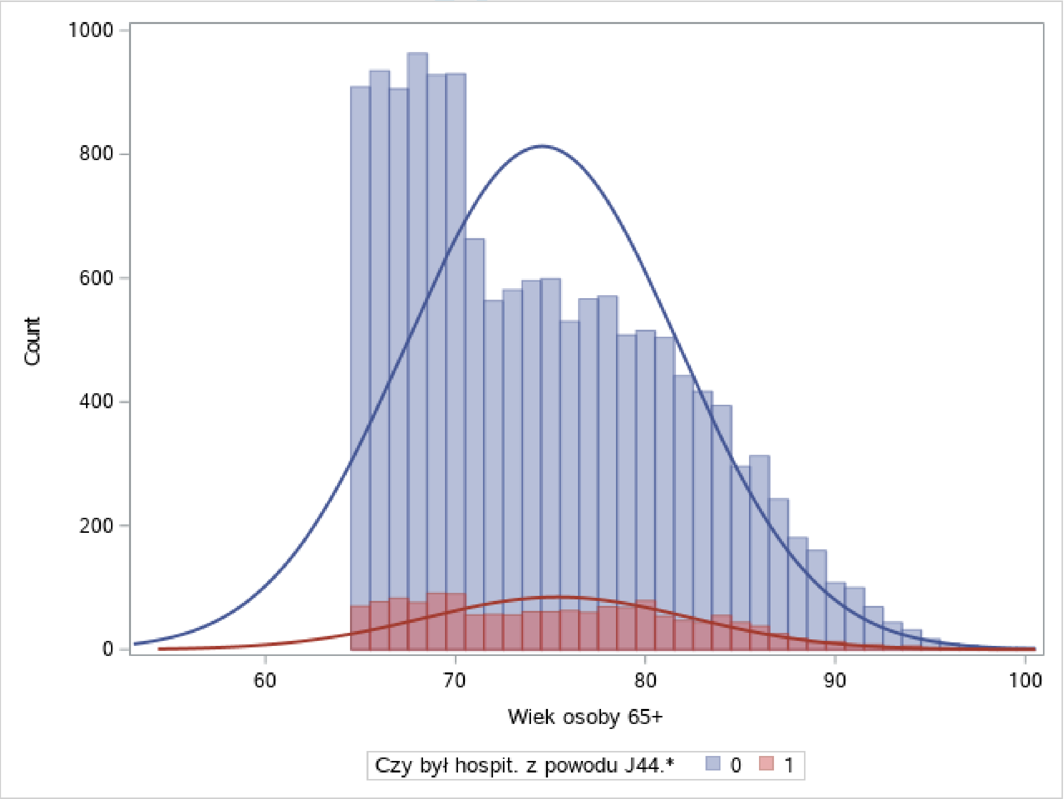

For peer review only - http://bmjopen.bmj.com/site/about/guidelines.xhtml

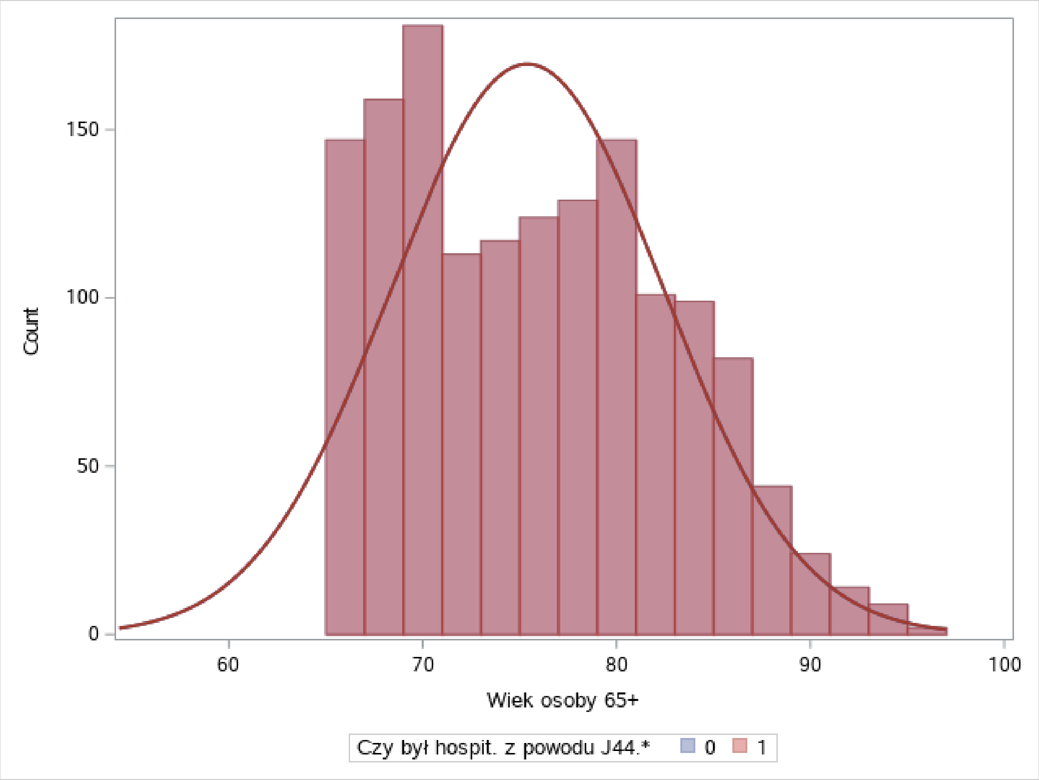

Histograms for the gender before and after PSM.

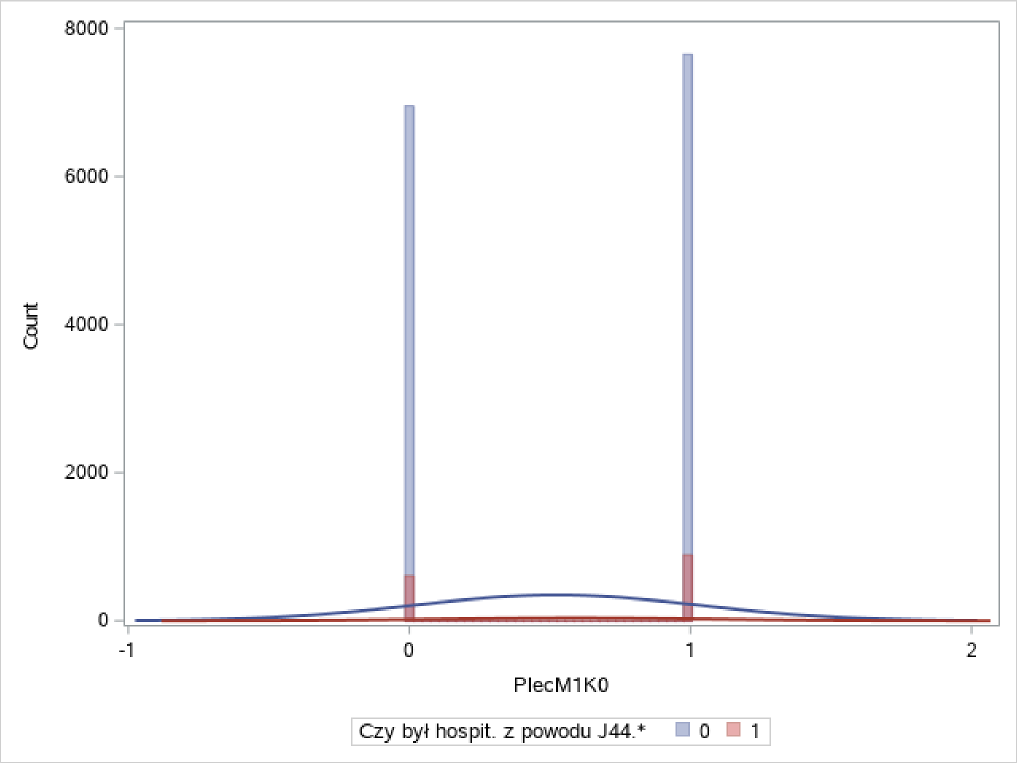

For peer review only - http://bmjopen.bmj.com/site/about/guidelines.xhtml

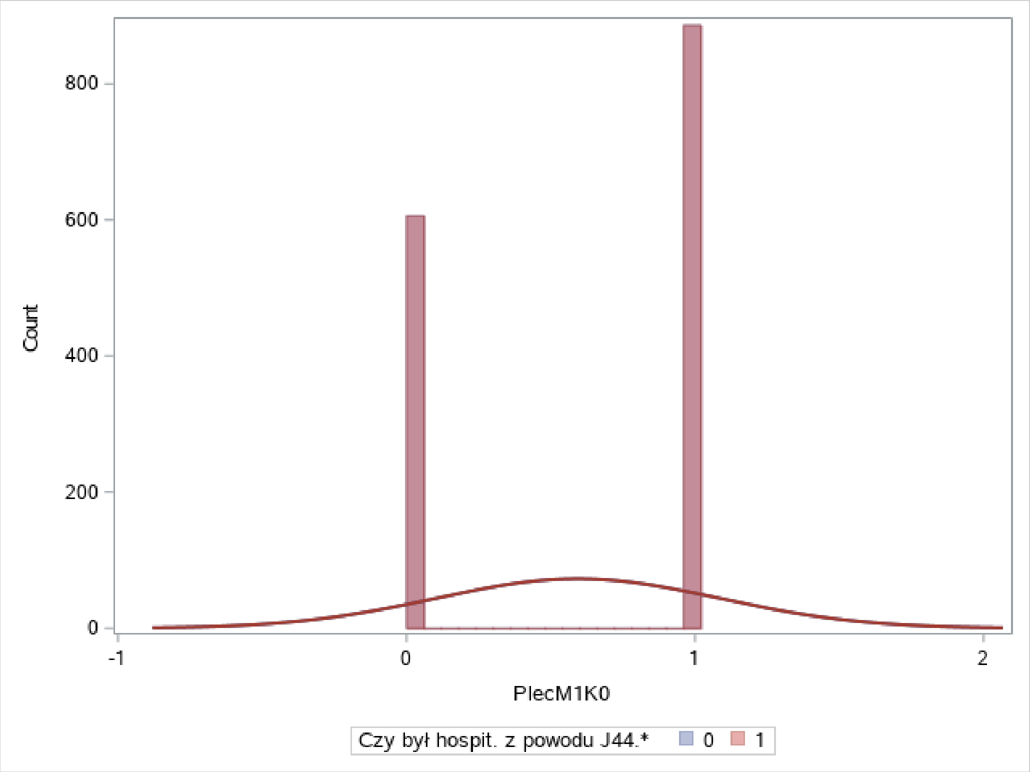

For peer review only - http://bmjopen.bmj.com/site/about/guidelines.xhtml

STROBE Statement—checklist of items that should be included in reports of observational studies

Title: Community determinants of COPD exacerbations in elderly patients in Lodz Province, Poland: a retrospective observational Big Data cohort study

	Item No	Recommendation	Page No
**Title and abstract**	1	(*a*) Indicate the study's design with a commonly used term in the title or the abstract	1-3
		(*b*) Provide in the abstract an informative and balanced summary of what was done and what was found	1-4
**Introduction**			
Background/rationale	2	Explain the scientific background and rationale for the investigation being reported	4-6
Objectives	3	State specific objectives, including any prespecified hypotheses	6
**Methods**			
Study design	4	Present key elements of study design early in the paper	5-8
Setting	5	Describe the setting, locations, and relevant dates, including periods of recruitment, exposure, follow-up, and data collection	5-6
Participants	6	(*a*) *Cohort study*—Give the eligibility criteria, and the sources and methods of selection of participants. Describe methods of follow-up<br>*Case-control study*—Give the eligibility criteria, and the sources and methods of case ascertainment and control selection. Give the rationale for the choice of cases and controls<br>*Cross-sectional study*—Give the eligibility criteria, and the sources and methods of selection of participants	6-7
		(*b*) *Cohort study*—For matched studies, give matching criteria and number of exposed and unexposed<br>*Case-control study*—For matched studies, give matching criteria and the number of controls per case	7
Variables	7	Clearly define all outcomes, exposures, predictors, potential confounders, and effect modifiers. Give diagnostic criteria, if applicable	6,8
Data sources/ measurement	8*	For each variable of interest, give sources of data and details of methods of assessment (measurement). Describe comparability of assessment methods if there is more than one group	6-7
Bias	9	Describe any efforts to address potential sources of bias	4,17
Study size	10	Explain how the study size was arrived at	8-9
Quantitative variables	11	Explain how quantitative variables were handled in the analyses. If applicable, describe which groupings were chosen and why	8
Statistical methods	12	(*a*) Describe all statistical methods, including those used to control for confounding	8
		(*b*) Describe any methods used to examine subgroups and interactions	-
		(*c*) Explain how missing data were addressed	8
		(*d*) *Cohort study*—If applicable, explain how loss to follow-up was addressed<br>*Case-control study*—If applicable, explain how matching of cases and controls was addressed<br>*Cross-sectional study*—If applicable, describe analytical methods taking account of sampling strategy	8

For peer review only - http://bmjopen.bmj.com/site/about/guidelines.xhtml

		(*e*) Describe any sensitivity analyses	-
**Results**			
Participants	13*	(a) Report numbers of individuals at each stage of study—eg numbers potentially eligible, examined for eligibility, confirmed eligible, included in the study, completing follow-up, and analysed	9
		(b) Give reasons for non-participation at each stage	-
		(c) Consider use of a flow diagram	-
Descriptive data	14*	(a) Give characteristics of study participants (eg demographic, clinical, social) and information on exposures and potential confounders	9-14
		(b) Indicate number of participants with missing data for each variable of interest	APPENDIX Page 9
		(c) *Cohort study*—Summarise follow-up time (eg, average and total amount)	9-12
Outcome data	15*	*Cohort study*—Report numbers of outcome events or summary measures over time	*9-12*
		Case-control study—Report numbers in each exposure category, or summary measures of exposure	*12-14*
		Cross-sectional study—Report numbers of outcome events or summary measures	*12-14*
Main results	16	(*a*) Give unadjusted estimates and, if applicable, confounder-adjusted estimates and their precision (eg, 95% confidence interval). Make clear which confounders were adjusted for and why they were included	14
		(*b*) Report category boundaries when continuous variables were categorized	14
		(*c*) If relevant, consider translating estimates of relative risk into absolute risk for a meaningful time period	-
Other analyses	17	Report other analyses done—eg analyses of subgroups and interactions, and sensitivity analyses	-
**Discussion**			
Key results	18	Summarise key results with reference to study objectives	14-17
Limitations	19	Discuss limitations of the study, taking into account sources of potential bias or imprecision. Discuss both direction and magnitude of any potential bias	4
Interpretation	20	Give a cautious overall interpretation of results considering objectives, limitations, multiplicity of analyses, results from similar studies, and other relevant evidence	14-17
Generalisability	21	Discuss the generalisability (external validity) of the study results	17
**Other information**			
Funding	22	Give the source of funding and the role of the funders for the present study and, if applicable, for the original study on which the present article is based	18

For peer review only

BMJ Open

Community determinants of COPD exacerbations in elderly patients in Lodz province, Poland: a retrospective observational Big Data cohort study

Journal:	*BMJ Open*
Manuscript ID	bmjopen-2021-060247.R1
Article Type:	Original research
Date Submitted by the Author:	07-Jun-2022
Complete List of Authors:	Kowalczyk, Anna; Medical University of Lodz, Centre for Family and Community Medicine<br>Kosiek, Katarzyna; Family Doctors' Clinic, Lodz, Poland<br>Godycki-Cwirko , Maciek; Medical University of Lodz, Centre for Family and Community Medicine<br>Zakowska, Izabela; Medical University of Lodz, Centre for Family and Community Medicine
**Primary Subject Heading**:	Respiratory medicine
Secondary Subject Heading:	Respiratory medicine, Public health, General practice / Family practice
Keywords:	Respiratory infections < THORACIC MEDICINE, PUBLIC HEALTH, PRIMARY CARE

SCHOLARONE™ Manuscripts

I, the Submitting Author has the right to grant and does grant on behalf of all authors of the Work (as defined in the below author licence), an exclusive licence and/or a non-exclusive licence for contributions from authors who are: i) UK Crown employees; ii) where BMJ has agreed a CC-BY licence shall apply, and/or iii) in accordance with the terms applicable for US Federal Government officers or employees acting as part of their official duties; on a worldwide, perpetual, irrevocable, royalty-free basis to BMJ Publishing Group Ltd ("BMJ") its licensees and where the relevant Journal is co-owned by BMJ to the co-owners of the Journal, to publish the Work in this journal and any other BMJ products and to exploit all rights, as set out in our licence.

The Submitting Author accepts and understands that any supply made under these terms is made by BMJ to the Submitting Author unless you are acting as an employee on behalf of your employer or a postgraduate student of an affiliated institution which is paying any applicable article publishing charge ("APC") for Open Access articles. Where the Submitting Author wishes to make the Work available on an Open Access basis (and intends to pay the relevant APC), the terms of reuse of such Open Access shall be governed by a Creative Commons licence – details of these licences and which Creative Commons licence will apply to this Work are set out in our licence referred to above.

Other than as permitted in any relevant BMJ Author's Self Archiving Policies, I confirm this Work has not been accepted for publication elsewhere, is not being considered for publication elsewhere and does not duplicate material already published. I confirm all authors consent to publication of this Work and authorise the granting of this licence.

For peer review only

Title page

Community determinants of COPD exacerbations in elderly patients in Lodz province, Poland: a retrospective observational Big Data cohort study

Authors:

Anna Kowalczyk[1], Katarzyna Kosiek[2], Maciek Godycki-Cwirko[1], Izabela Zakowska[1]

1. Centre for Family and Community Medicine, the Faculty of Health Sciences, the Medical University of Lodz, Poland
2. Family Doctors' Clinic, Lodz, Poland.

Corresponding author:

Izabela Zakowska

Centre for Family and Community Medicine, Faculty of Medical Sciences,

Medical University of Lodz,

Kopcinskiego 20, 90-153 Lodz, Poland

izabela.zakowska@umed.lodz.pl

tel.: +48 42 679 55 46

Key words: COPD, Big Data, Exacerbations, Primary Health Care, Electronic Health Records.

Word count: 3828

For peer review only

Abbreviations:
65+ - patients 65 years old and older
AECOPD – Acute Exacerbations of chronic obstructive pulmonary disease
ASC – Ambulatory Specialists Care
ATC - Anatomical-therapeutic-chemical classification
COPD - Chronic Obstructive Pulmonary Disease
EHRs - electronic health records
Gmina-administrative part of province
GUS - National Statistics Center (Statistics Poland)
ICD-10 - International Classification of Diseases
NFZ - National Health Fund
PHC – Primary Health Care
PSM – Propensity Score Matching
SES - socioeconomic status
SOR - Hospital Emergency Department
US - Tax Office (Revenue Administration Regional Office in Lodz)
TERYT - Registry identifiers (GUS)
ATC group - Anatomical Therapeutic Chemical (ATC) Classification
R03 - drugs for obstructive airway diseases
R03A - adrenergics, inhalants:
*R03AC - Selective beta-2-adrenoreceptor agonists
*R03AK - Adrenergics in combination with corticosteroids or other drugs, excl. anticholinergics
*R03AL - Adrenergics in combination with anticholinergics incl. triple combinations with corticosteroids
R03B - other drugs for obstructive airway diseases, inhalants:
*R03BA – Glucocorticoids
*R03BB - Anticholinergics
R03D - other systemic drugs for obstructive airway diseases:
*R03DA - Xanthines
*R03DC - Leukotriene receptor antagonists

ABSTRACT

Objectives To evaluate the prevalence and identify demographic, economic and environmental local community determinants of COPD exacerbations in elderly in primary care using Big Data approach.
Design Retrospective observational case-control study. based on Big Data from the National Health Found, Tax Office and National Statistics Center databases in 2016.
Setting Primary care clinics in the Lodz province in Poland.
Participants 472 314 patients aged 65 and older in primary care including 17 240 COPD patients, and 1784 with exacerbations (including deaths).
Outcome measures Exacerbations with demographic, economic and environmental local community determinants were retrieved. Conditional logistic regression for matched pairs was used to evaluate the local community determinants of COPD exacerbations among COPD patients.
Results The overall prevalence of COPD in the population of elderly patients registered in PHC clinics in Lodz province in 2016 was 3.65%, 95%CI (3.60-3.70) [%] and the prevalence of exacerbations was 10.35%, 95%CI (9.89-10.80) [%]. The high number of consultations in primary care clinics was associated with higher risk of COPD exacerbations (p=0.0687).

For peer review only

High-income patients were less likely to have exacerbations than low-income patients (high vs low OR = 0.601, 95% CI (0.385-0.939)). The specialization of the primary care physician did not have an effect on exacerbations (OR = 1.076, 95% CI (0.920-1.257)). Neither the forest cover per gmina (high vs low OR = 0.897, 95% CI (0.605-1.331); medium vs low OR = 0.925, 95% CI (0.648-1.322)), nor location of gmina (urban vs. urban-rural OR = 1.044; 95% CI (0.673-1.620)), (rural vs. urban-rural OR = 0.897, 95% CI (0.630-1.277)) appears to influence COPD exacerbations.

Conclusions Big Data statistical analysis facilitated the evaluation of the prevalence and determinants of COPD exacerbation in the elderly residents of Lodz province, Poland.

Modification of identified local community determinants may potentially decrease the number of exacerbations in COPD elderly patients.

For peer review only

Strengths and limitations

- Retrieving knowledge from three relevant community big data bases (National Health Found, Tax Office and National Statistics Center).
- Evaluation of prevalence of COPD and determinants of COPD exacerbations in elderly primary care patients in Lodz province in Poland.
- Its main limitation was the lack of standardization in the method of recording exacerbations between primary care, accident and emergency departments and hospital wards.
- No data existed for some parameters, such as air quality for gaseous and dust pollutants, at the commune level for examined period.

INTRODUCTION

Chronic obstructive pulmonary disease (COPD) is the third leading cause of death worldwide, causing 3.23 million deaths in 2019. [1] Despite the great effort directed at prevention and treatment guidelines, the prevalence continues to increase and is now estimated to range from 4% to 10% worldwide; this value varies considerably across the European union, i.e. from 1.26% to 13.87%, partly because of the differences in the definitions used. [2-4] In Poland, a medium-sized country with population of about 38 million inhabitants, studies suggest a COPD prevalence of 11% in the cites of Warsaw and Lublin; however, data is scarce in other regions. [5]

COPD is a progressive disease characterized by exacerbations, i.e. an acute worsening of the respiratory symptoms which may lead to hospitalization [6-8]. In addition, patients with stable COPD often have acute exacerbations (AECOPD) due to risk factors, comorbidities, poor treatment adherence and insufficient treatment. [9] Frequent exacerbations, defined as at least

For peer review only

two episodes per year, are considered a marker of increased burden and reduced survival in clinical epidemiology studies. [10]

Before such exacerbations can be effectively prevented, there is a need to identify the factors contributing to them. To date, most studies have focused on factors specific to COPD patients themselves, rather than on external factors. [11, 12] However, it is possible that Socioeconomic status (SES) may play a role. SES is a complex assessment of a person's economic and social position in society based on several factors including income, occupation, home and neighborhood environment, and educational attainment. [13]

COPD appears to have a stronger socioeconomic element than other common causes of morbidity and death, including cardiac disease. [13] Also, low income appears to have a greater influence on the prevalence of COPD than asthma. [14] In Canada, where the overall prevalence of COPD was 5.69%, this prevalence was found to be significantly higher among older patients, and those who are currently smoking, underweight or obese, and those with total personal income <$ 20,000 and some post-secondary education. [15]

A number of environmental factors can have effects on health, both positive and negative..

Green spaces, parks and community gardens support community health by reducing stress, promoting physical activity, and improving perceived general health. [16] These measures can be identified and modified to promote positive health behaviors and avoid negative ones, [17] and to shape specific promotional and educational initiatives aimed at reducing the prevalence of COPD and its exacerbations.

Recently, *Big Data* analyses have made it possible to measure, analyze, and document the health of individuals and populations, with the aim of improving public health and enhancing decision making at the individual and community levels. [18] Such applications have been used to monitor the quality of medical and healthcare facilities, improve patient service, detect

For peer review only

disease spread earlier, provide new insights into disease mechanisms through risk factors, and improve treatments. [19, 20]

Earlier studies on COPD exacerbations have been mostly prospective and have involved patients from secondary and/or tertiary care. In contrast, the present study examines associations between well-known patient-level risk factors and triggers of exacerbations of COPD, including local community factors.

OBJECTIVES

To estimate the prevalence of COPD in elderly patients in the province of Lodz Poland, and to determine the prevalence of AECOPD in this population.

To identify local community determinants of disease exacerbations among elderly COPD patients.

METHODS

A retrospective observational case-control cross-sectional study was performed among elderly patients with COPD in the Lodz province in Poland. The study was conducted between January 1st and December 31st, 2016.

DATA SOURCES

Data were retrieved from three Big Data databases of the NFZ (National Health Service), US (Tax Office) and GUS (Central Statistical Office).

The NFZ electronic health records (EHRs) database was used to gain information on sociodemographic (age, sex), inpatient and outpatient healthcare utilisation, including costs of hospitalization, and the cost of dispensed medications in the form of ATC (anatomical

For peer review only

therapeutic chemical) codes, as well as other corresponding costs and information on physicians working in primary health care clinics (PHC) in the study area.

The US database provided information on the income of residents aged 65 and older (65+) in the province of Lodz.

The GUS database contained variables potentially associated with exacerbations for 177 gminas (local communities) within the Lodz province.

Local community factors potentially associated with COPD exacerbations in the elderly were selected and classified by experts into (1) health factors, (2) socioeconomic factors and (3) community environmental factors at gmina level, according to the Remington and Catlin methodology [21].

PARTICIPANTS

All participants were patients registered in PHC clinics, aged 65+, residents of Lodz province. All were assigned to a particular gmina; it was assumed that the majority of patients aged 65+ choose a healthcare clinic close to their place of residence. COPD was indicated by the presence of the International Classification of Diseases (ICD-10) code J44 in patients' medical records; exacerbations were defined as cases "hospitalized with the J44 code as a main reason for admission".

Big Data Quality process

The Big Data quality process was carried out according to the SAS Data Management Methodology. After cleaning the data, the rules and alerts for monitoring were defined (details given in APPENDIX).

STATISTICAL ANALYSIS

For peer review only

The data was analysed as described previously in protocol. [22] Additionally, the propensity score matching (PSM) technique and multivariate conditional logistic regression for matched pairs were applied to estimate the local community determinants of AECOPD. [23]

Descriptive statistics were calculated for the total cohort of COPD patients aged 65+ before and after propensity score matching (PSM). Missing data was excluded from the analysis. The relationships between tested variables and exacerbations (1-with, 0-without) were determined using univariate high-performance logistic regression models, and univariate logistic regression models applied and stratified logistic regression (a single model is for each line). Continuous variables were categorized by quartile. Pearson's correlation coefficient was estimated. Patients with exacerbations were matched retrospectively for gender and age to patients without exacerbations to ensure 1:1 group ratio without repetition using propensity score matching (PSM). Patients who died were excluded from the analysis. Costs were calculated as the percentage of costs per patient to the mean COPD patient population and categorized by quartiles (APPENDIX).

After adjusting for potential covariates, the odds ratio (OR) and 95% confidence intervals 95% (CI) of exacerbations between cases and control patients were calculated by multivariate conditional logistic regression for matched pairs models. For the prevalence of the disease, 95% confidence intervals (95%CI) of the fraction of patients in the population were calculated.

Statistically significant findings were assumed as p-value <0.05.

The analysis was performed with the SAS V.9.4 statistical package, MLwiN V.2.24 and STATISTICA V.13.1.

Study size/Power Calculation

The power was calculated for paired t-test for mean difference. Taking number of pairs 1492, calculated mean difference between number of consultations in PHC clinic for patients with

For peer review only

exacerbation and without exacerbations was 1.214 (SD=9.658). The standard deviation for the number of consultations in PHC clinic in group with exacerbation was 7.62 and in group without exacerbation was 6.76 , the correlation between them was 0.103 (p<0.0001), which gave the power of 99.8%. Total of 499 pairs would allow to 80% power.

Patient and Public Involvement

This was a Big Data study using patients' anonymized electronic health records without patient involvement.

RESULTS

Prevalence

The Lodz province comprised 2 485 323 inhabitants as of December 31, 2016 [24]. According to the NFZ electronic health records (EHRs) database within the Lodz province, 472 314 elderly patients were registered in PHC clinics with contracts with the National Health Fund (19% of total number of residents).

Prevalence of COPD in the population of elderly patients registered in PHC clinics in Lodz province in 2016 was 3.65% (95%CI (3.60-3.70) [%]) (17 240 patients coded as J44) of all patients (n= 472 314). Among all COPD patients, 10.35%, 95%CI (9.89-10.80) [%] (1 784) were recorded as having an exacerbation; this group represents 0.38% of the entire elderly patient population in the Lodz province. During the study, 1 141 of 17 240 (6.62%) patients died and their data was excluded from further analysis. In total, 16 099 COPD patients were included in the analysis.

Characteristics of the COPD patient groups (raw data before PSM)

The group characteristic is presented in Supplementary table 1S and APPENDIX 1A.

For peer review only - http://bmjopen.bmj.com/site/about/guidelines.xhtml

In total, 1 492 (9.27%) of 16 099 COPD patients reported exacerbations. The number of exacerbations per individual COPD patient ranged from one to six. Most of these patients reported one exacerbation (1 294 patients; 86.73%), with fewer reporting two (148 patients; 9.92%), three (32 patients; 2.14%), four (11 patients; 0.74%); five (five patients; 0.34%) and six exacerbations (two patients; 0.13%).

A statistically significant relationship was observed between sex and exacerbations ($p < 0.0001$). 59.38% of patients in group with exacerbation was male. In addition, a significant relationship was noted between age and exacerbations ($p<0.0001$): the COPD group with exacerbations had a higher median age 75 years old (Lower Quartile= 69, Upper Quartile =81) than the COPD patients without exacerbations 73 years old (q1=68, q3=80).

The number of COPD consultations in PHC clinics, classed as the number of health care consultations with the main diagnosis J44, ranged from 0 to 24 per patient during the study year, with the most common value being one consultation in PHC clinic per COPD 65 patient. Among the people with exacerbation 36.73% did not have any COPD-related consultations in the PHC clinic and 1.14% in the group without exacerbation. 19.17% patients in group with exacerbations had received one COPD consultation (52.72% in group without exacerbation), 12.8% two consultations (20.06% in group without exacerbation), 7.91% three consultations (10.36% in group without exacerbation), 23.39% four and more COPD consultations (15.72% in group without exacerbation).

The number of COPD consultations in 2016 was associated with the number of exacerbations ($p<0.0001$).

The number of consultations for any reason in PHC clinic per 65 +, COPD patient during the study year ranged from 0 to 78, with the most common value being 10 consultations.

The number of consultations in PHC in 2016 were significantly associated with disease exacerbation (medium vs. low $p = 0.0350$ and high vs. low $p = 0.0092$). Among patients with

For peer review only - http://bmjopen.bmj.com/site/about/guidelines.xhtml

exacerbation, about 46% had 12 or more consultations, 44% had a medium number of consultations (n=5 to 12), and only 10% had low number (n=0 to 4).

In total, 2 465 physicians were working in PHC clinics within the Lodz province, including 627 (25.44%) family doctors, and 1 838 (74.56%) other physicians (general internal medicine, pediatrics, physicians without specialization). Of the total physicians, 1 674 (67.91%) were female. The median age of physicians was 52 (min. 25, max. 88) years old.

Similar percentages of patients whose physician was a family doctor vs. other were observed in the group of COPD patient with exacerbations (39.34%) and in the group without exacerbations (37.54%) ($p=0.1703$).

There was an association between the numbers of packages of reimbursed drugs and exacerbations (R03 AC, AL, BA, BB, DA: $p = 0.0001$, AK $p = 0.0015$, R03DC $p= 0.4080$), although this variables had n=1906 (11.84%) of missing data and need to be carefully interpreted.

The COPD group with exacerbations demonstrated higher numbers of packages of reimbursed drugs with ATC codes R03 AC, AL, BA, BB, DA AK except R03DC compared to those without exacerbations, based on prescriptions provided to the patient.

The NFZ drug reimbursement costs per patient were significantly associated with exacerbations ($p<0.0001$, n=1906 (11.84%) of missing data). There are also a significant associations between total stationary costs and exacerbations ($p <0.0001$) (APPENDIX). The total NFZ costs per patient (as sum of costs of PHC, ASC, hospitalization, drug reimbursement and other) was significantly associated with exacerbations ($p <0.0001$) (APPENDIX table 3A).

Income was also significantly associated with exacerbations ($p <0.0001$).

The group with exacerbations demonstrated lower income, by PLN 584.02 per year, compared to the group without exacerbations.

For peer review only

Forest cover of the gmina where the patient lived had a significant impact on the exacerbations (high vs. low p=0.0014, medium vs. low p=0<0.0001). 43% of patients in group with exacerbations were residents of gminas with a lower level of forest cover.

COPD exacerbations were found to be significantly associated with patient's place of residence in gmina (urban, rural, urban-rural).

Patients with COPD exacerbations throughout the one-year study period were matched for gender and age at baseline with patients without COPD exacerbations in a 1:1 ratio: n=1 492 cases (50%) and n=1 492 controls (50%) (before and after PSM: Histograms Fig.1 – Fig.4 in APPENDIX).

The characteristics of COPD patients with and without exacerbations after PSM are given in Table 1and tables in APPENDIX 2A and Supplementary materials 2S.

Table 1. Descriptive characteristics and local determinants univariate analysis of COPD exacerbations in cohort of COPD elderly patients (Lodz province, 2016; case-control). After PSM.

PSM analysis identified the similar relationships between variables and exacerbations as in the raw data. The median age was 75 (q1=69, q3=81) years for J44 with exacerbations and 75 (q1=69, q3=81) for J44 without exacerbations.

Patients' consultations in PHC clinics, and COPD consultations in PHC clinic were statistically associated with exacerbations (high vs. low p = 0.0687 border of significance, p < 0.0001, respectively).

High number of consultations in PHC clinics (any reason) had 46.18% of patients in group with exacerbations and 37.40% in group without exacerbations.

In addition, 36.73% patients with exacerbations and 0.87% of patients without exacerbations had not received any COPD consultations in PHC clinic. When the number of consultations in PHC with the main diagnosis J44 increased from one to three, a decrease in the number of exacerbations was observed.

There was an association between the numbers of packages of reimbursed drugs and exacerbations (R03 AC, AL, BA, BB, DA: p = 0.0001, AK p = 0. 0.0078, R03DC p= 0. 0.9022. Patients with exacerbations used a greater number of prescriptions for reimbursed drug packaging The patients with exacerbations realized higher numbers of prescriptions of reimbursed drugs from ATC groups with codes R03AC, AL, BA, BB, DA, AK compared to those without exacerbations. Again the numbers of R03DC packages did not differ significantly between the groups.

Significant association was found between total NFZ cost per patient (as the sum of costs of PHC, ASC, hospitalization, drugs reinvestment and other) and COPD exacerbation (p = <0.0001) (APPENDIX table 3B).

Of all patients in the exacerbations group, 37.53% had high total NFZ cost per patient, and 58.65% had medium. Among those without exacerbations, the costs were high in only 12%, medium in 41%, and 46% had low costs.

A significant association was found between income and disease exacerbations (high vs. low p= 0.0439). There were fewer exacerbations in the group of patients with high income.

There were no association between the forest cover of the patient's place of residence in gmina and exacerbations (high vs. low p=0.5082, medium vs. low p=0.6986).

Model of local determinants of COPD exacerbations for elderly COPD patients

For peer review only

Table 2 shows local determinants of COPD exacerbations for 65+ patients. For more descriptive characteristics of the determinants please see Supplementary materials and APPENDIX.

Table 2. Local determinants of COPD exacerbations in cohort of COPD elderly patients (Lodz province, 2016; case-control). After PSM.

The final model includes five independent variables: number of consultations in PHC, income, physicians' specialization in PHC, the area covered with forest in gmina of patient's residence and location of gmina (urban, rural, urban-rural).

Among them, two variables turned out to be significant: number of consultations in PHC (border significance) and income.

Patients with a medium number of consultations in PHC demonstrated insignificantly lower chance of exacerbations than those with a low number of consultations (medium <5-12> vs low (<5) OR=0.823, 95% CI (0.643-1.053)) (table 2).

Those COPD patients with high number of consultations (above 12) in PHC had 1.26 the odds of having exacerbations relative to those with low consultation number (less than 5), but this was of borderline significance (high (>12) vs low (<5): OR=1.261, 95% CI (0.974-1.633)).

COPD patients living in areas with a high income had a lower chance of exacerbations than those in areas with a low income (high vs low OR = 0.601 (0.385-0.939)). Living in a high income area is a positive and protective factor vs. living in a low income one.

Type of physician's specialization in PHC had no influence on the exacerbations (family doctor vs other OR = 1.076, 95% CI (0.920-1.257) neither the forest cover area (high vs low OR=0.897, 95% CI (0.605-1.331), medium vs low OR=0.925, 95% CI (0.648-1.322)) or patient's place of residence in gmina (urban vs. urban-rural OR=0.897, 95% CI (0.630-1.277), rural vs. urban-rural).

For peer review only

DISCUSSION

Despite significant advances in the diagnosis and treatment of COPD, its incidence remains high. In addition, in some cases, the chance of exacerbation can be increased as a result of local risk factors.

Fortunately, nowadays information on community health and health care system can be easily gathered from a number of large databases, as Big Data, and analyzed statistically. [25]

Big Data technology can be used to improve the course of chronic disease and its exacerbations. For example, Korean researchers were able to precisely match COPD exacerbations with external factors. [26, 27]

Similarly, the present study applied Big Data analysis to a large primary care cohort of 472 314 patients aged 65 years and older in the Lodz province, Poland. The results yielded new information on the disease itself and its relationship with local community factors (demographic factors, healthcare use, economic and environmental factors).

A meta-analysis of COPD prevalence reported a pooled prevalence of 9.8% among men and 5.6% among women. [28]

In present study, this value was 3.65% among all elderly patients. COPD exacerbations, hospitalizations and deaths in women have been increasing in several countries, with more women being hospitalized for COPD in recent years [29]; indeed, the percentage of hospitalizations among women in Puglia, Italy was found to increase from 27.8% in 2001 to 35.6% in 2011. [30]

However that tendency was not found in our study where 59.38 % of total COPD patients with exacerbation were male (raw data p<0.0001).

For peer review only - http://bmjopen.bmj.com/site/about/guidelines.xhtml

More research is needed to verify these apparent gender differences, not only among hospitalized patients but also in their daily environment.

In a Danish study, the risk of hospitalization for COPD increased with age until ~80 years for females and 85 years for males. [31]

Our findings also indicate a significant relationship between age and exacerbation (raw data p <0.0001). Of those with exacerbations, 47% are aged 65-75 years, 40% are aged 75-85 years, and approximately 14% are 85 years of age or older. In our study, the age of patients in the exacerbation group was higher than in group without exacerbation: 75 years old vs. 74 (raw data p<0.0001)

The high number of primary care consultations per patient and high income in the local area were also significant determinants of exacerbations among COPD patients aged 65+ (p=0.0784 border of significance and p=0.0253, respectively), controlled for the specialization of the physician in the PHC, forest cover of the local area and the location of the gmina.

In the present study, patients with a higher number of consultations have more than 1.26 times the chances of exacerbations compared to patients with a low number of consultations, but this was on the border of statistical significance (OR= 1.261; 95% CI=0.974, 1.633).

It is likely that the former group have a greater chance of referral to a hospital by a primary care physician, and thus faster diagnosis and treatment.

Patients with high income are less likely to have COPD exacerbations than those with low income (OR= 0.601; 95% CI 0.385, 0.939). Other studies have reported similar trends. [32] This may be related to decreased access to health care services among lower income groups. Such problems with access may delay the diagnosis of COPD, leading to a poorer prognosis and increased morbidity and mortality. [33] Addressing this issue may reduce the workload of the healthcare system and allow more targeted use of available resources.

The association between neighbourhood greenness and respiratory outcomes in adults appears ambiguous. [34] One cohort study including 1.3 million adults in Canada found that greater neighbourhood greenness could reduce the risk of respiratory mortality [35]; however, a Chinese cohort study concluded that neighborhood greenness might in fact be a risk factor. [36] In our present study, forest cover per gmina was not found to have any statistically significant effect on the COPD exacerbation.

Rural residence has been found to be associated with an increased incidence of COPD exacerbations, although not severe ones. [37] In countries with large rural populations, COPD is more prevalent in rural than urban areas. This disparity may be related to increased agricultural exposure (wheat, grain, cotton dust, pesticides), among others. [38] In addition, studies suggest this may be associated with increased indoor air exposure associated with burning biomass fuels. [39]

In our study, patient's place of residence in gmina (urban, rural, urban-rural) did not appear to have any influence on the COPD exacerbations.

In addition, the specialization of the physician in primary care did not have any effect on the COPD exacerbations.

Identification of patient population relied only on the ICD-10 J-44 code in patients primary care records, so COPD patient in specialist clinics and on hospitals wards without this ICD-10 code might not be identified, what can be perceived as study limitation. The validation of the J-44 Code of the patients enrolled in the study was not possible at the PHC clinics level because the NFZ provided us with anonymised patient data.

Patients with code J44 were included in this study, without excluding codes for other diseases, including asthma, due to missing data in the variable "Codes for comorbidities with COPD" (98.83% (466 780 out of 472 314) in total 65+ patients in PHC in Lodz province in 2016), so the variable was excluded from the analysis.

For peer review only

Another limitation of this study was lack of standardization in the method of recording exacerbations between primary care, accident and emergency departments and hospital wards.

CONCLUSIONS

This study investigates the determinants for AECOPD with the aim of developing activities to improve prevention and treatment interventions. Our findings regarding COPD in the Lodz province, Poland may be also used for formulating health policy recommendations by the National Health Fund (NFZ) and local government. The Big Data methodological approach used herein can be applied to other chronic diseases. However, further study is needed to determine the role of local medical and environmental factors in improving the health of COPD patients.

ACKNOWLEDGMENTS

We acknowledge the role of experts from various fields: primary care physicians, public health specialists, a Big Data analyst and data managers in public institutions (NFZ, GUS and US) in the study.

The authors specially thank Edward Lowczowski for English language assistance.

Funding Statement

This work was supported by Narodowe Centrum Nauki (National Science Centre, Poland) grant number 2016/21/B/NZ7/02052.

Competing interests statement.

No other relationships or activities that could appear to have influenced the submitted work.

Data sharing

No additional data available.

Research Ethics Approval

The study protocol has been approved by the Bioethical Committee of Medical University of Lodz (RNN/248/18/KE, 10 July 2018).

This Big Data study using patients' anonymized electronic health records data.

Author's contribution

The study design was conceptualized by A.K., M.G.-C K.K. and I.Z. Analyses were performed by I.Z. K.K. and I.Z. prepared the first draft of the manuscript. All authors have read and agreed to the published version of the manuscript.

For peer review only

Table 1. Descriptive characteristics and local determinants of COPD exacerbations in cohort of COPD elderly patients (Lodz province, 2016; case-control). After PSM.

**Patient Characteristic (After PSM)**	**COPD patients without exacerbation Control (n=1492 pairs)**	**COPD patients with exacerbations Cases (n=1492 pairs)**	**Total (n=2984)**	**OR (95% CI) Univariate analysis****
**Patient level**				
**Demographic**				
**COPD Patients (Sum)(%) Total**	1492 (50)	1492 (50)	2984 (100)	
**Sex (strata variable)**				
**Male**	886 (59.38)	886 (59.38)	1772 (59.38)	1 Ref. Cat^.
**Female**	606 (40.62)	606 (40.62)	1212 (40.62)	1.000 (0.864-1.157)^
**Age (years) (strata variable)**				
**<75-85)**	600(40.21)	600(40.21)	1200(40.21)	1.000 (0.788-1.269)^
**<85+>**	175(11.73)	175(11.73)	350(11.73)	1 Ref. cat.^
**<65-75)**	717(48.06)	717(48.06)	1434(48.06)	1.000 (0.792-1.263)^
**Patient's place of residence in gmina (localization)**				
**1-urban vs. 3**	862 (57.77)	864 (57.91)	1726 (57.84)	1.028 (0.848-1.246)
**2-rural vs. 3**	353 (23.66)	358 (23.99)	711 (23.83)	1.04 (0.833-1.3)
**3-urban-rural (Ref. Cat.)**	277 (18.57)	270 (18.1)	547 (18.33)	1 Ref. Cat.
**Healthcare uses**				
**Specialization of physician working in PHC**				
**Family doctors vs. Other**	562(37.67)	587(39.34)	1149(38.51)	1.080(0.926-1.259)
**Other (Ref. Cat.)**	930(62.33)	905(60.66)	1835(61.49)	1 Ref. Cat.
**Number of consultations in PHC^**				

For peer review only

**high (>12) vs. low (<5)**	558 (37.4)	689 (46.18)	1247 (41.79)	1.269 (0.982-1.639)
**medium <5-12> vs. low (<5)**	783 (52.48)	649 (43.5)	1432 (47.99)	0.831 (0.651-1.061)
**low (<5) (Ref. Cat.)**	151 (10.12)	154 (10.32)	305 (10.22)	1 Ref. Cat.
**Number of COPD consultations in PHC^**				
**0 vs. 4+**	13 (0.87)	548 (36.73)	561 (18.8)	41.078 (18.064-93.413)
**1 vs. 4+**	751 (50.34)	286 (19.17)	1037 (34.75)	0.288 (0.224-0.37)
**2 vs. 4+**	299 (20.04)	191 (12.8)	490 (16.42)	0.488 (0.369-0.645)
**3 vs. 4+**	185 (12.4)	118 (7.91)	303 (10.15)	0.528 (0.377-0.738)
**4+ (Ref. Cat.)**	244 (16.35)	349 (23.39)	593 (19.87)	Ref. Cat.
**Gmina/Commune, post-codes level**				
**Socioeconomic Status**				
**Total personal income of residents per PHC post-codes (Tax Office)**				
**high (>21056,52) vs. low (<17079,50)**	371 (24.87)	345 (23.12)	716 (23.99)	0.696 (0.489-0.990)
**medium <17079,50-21056,52> vs. low (<17079,50)**	759 (50.87)	768 (51.47)	1527 (51.17)	0.910 (0.722-1.147)
**low (<17079,50) (Ref. Cat.)**	362 (24.26)	379 (25.4)	741 (24.83)	1 Ref. Cat.
**Environmental factors**				
**Forest cover of the patient's place of residence in gmina (GUS)**				
**high (>20,60%) vs. low (<7,00%)**	348 (23.32)	340 (22.79)	688 (23.06)	0.896 (0.648-1.24)
**medium <7,00%-20,60%> vs. low (<7,00%)**	776 (52.01)	776 (52.01)	1552 (52.01)	0.94 (0.688-1.285)
**low (<7,00%) (ref. Cat.)**	368 (24.66)	376 (25.2)	744 (24.93)	1 Ref. Cat.
**Total**	1492 (50)	1492 (50)	2984 (100)	

CI=Confidence Interval, OR=Odds Ratio. #=Odds Ratio Estimates (Point Estimate). 95% Wald Confidence Limits. OR (95% CI).
Ref. cat.=reference category.
^- Sex and age were strata variables (the LOGISTIC Procedure, Logistic Regression).
**The LOGISTIC Procedure, Conditional Logistic Regression for Matched Pairs.

For peer review only

Table 2. Local determinants of COPD exacerbations in cohort of COPD elderly patients (Lodz province, 2016; case-control). After PSM.

	**Final model Conditional Logistic Regression for Matched Pairs**
**Local determinants of COPD exacerbations (After PSM)**	**OR (95% Wald Confidence Limits), (point estimate).**
**Patient level**	
**Demographic**	
**COPD Patients (Sum)(%) Total**	N=1492 pairs
**Patient's place of residence in gmina (localization)**	
**1-urban vs. 3**	1.044 (0.673-1.620)
**2-rural vs. 3**	0.897 (0.630-1.277)
**3-urban-rural (Ref. Cat.)**	1 Ref. Cat.
**Healthcare uses**	
**Specialization of physician working in PHC**	
**Family doctors vs. Other**	1.076 (0.920-1.257)
**Other (Ref. Cat.)**	1 Ref. Cat.
**Number of consultations in PHC^**	
**high (>12) vs. low (<5)**	1.261 (0.974-1.633)
**medium <5-12> vs. low (<5)**	0.823 (0.643-1.053)
**low (<5) (Ref. Cat.)**	1 Ref. Cat.
**Gmina/Commune, post-codes level**	
**Socioeconomic Status**	
**Total personal income of residents per PHC post-codes (Tax Office)**	
**high (>21056,52) vs. low (<17079,50)**	0.601 (0.385-0.939)
**medium <17079,50-21056,52> vs. low (<17079,50)**	0.824 (0.581-1.169)
**low (<17079,50) (Ref. Cat.)**	1 Ref. Cat.
**Environmental factors**	
**Forest cover of the patient's place of residence in gmina (GUS)**	
**high (>20,60%) vs. low (<7,00%)**	0.897 (0.605-1.331)
**medium <7,00%-20,60%> vs. low (<7,00%)**	0.925 (0.648-1.322)
**low (<7,00%) (ref. Cat.)**	1 Ref. Cat.

OR=Odds Ratio Estimates (Point Estimate). 95% Wald Confidence Limits. Ref. cat.=reference category.
#The LOGISTIC Procedure, Model binary logit, 1:1 Matched Case-Control Conditional Logistic Regression for Matched Pairs. Sex and age variables were included as stratification variables (the 1:1 matched case-control), thus they are not explicitly included in the Conditional Logistic Regression for Matched Pairs models.

For peer review only

References

1. WHO. Newsroom. The top 10 causes of death. WHO's Global Health Estimates 2021 [05.11.2021]: Available from: https://www.who.int/news-room/fact-sheets/detail/the-top-10-causes-of-death
2. Adeloye D, Chua S, Lee C, Basquill C, Papana A, Theodoratou E, et al. Global and regional estimates of COPD prevalence: Systematic review and meta-analysis. *J. Glob. Health* 2015; 5:020415.
3. Boven Jv, Gaughan J, Soriano J, Correia de Sousa J, Baxter N, Román-Rodríguez M, et al. P139 The burden of copd across the european union: development of the european copd atlas. *Thorax* 2016;71(Suppl 3):A158-A.
4. Gibson GJ, Loddenkemper R, Lundback B, Sibille Y. Respiratory health and disease in Europe: the new European Lung White Book. *Eur Respir J* 2013; 42:559-63.
5. Blanco I, Diego I, Bueno P, Fernandez E, Casas-Maldonado F, Esquinas C, et al. Geographical distribution of COPD prevalence in Europe, estimated by an inverse distance weighting interpolation technique. *Int J Chron Obstruct Pulmon Dis* 2018;13:57-67.
6. Mullerova H, Shukla A, Hawkins A, Quint J. Risk factors for acute exacerbations of COPD in a primary care population: a retrospective observational cohort study. *BMJ Open* 2014;18:e006171.
7. Perera PN, Armstrong EP, Sherrill DL, Skrepnek GH. Acute exacerbations of COPD in the United States: inpatient burden and predictors of costs and mortality. *COPD* 2012;9:131-41.
8. Qureshi H, Sharafkhaneh A, Hanania NA. Chronic obstructive pulmonary disease exacerbations: latest evidence and clinical implications. *Ther Adv Chronic Dis* 2014;5:212-27.
9. Esteban C, Quintana JM, Moraza J, Aburto M, Egurrola M, Espana PP, et al. Impact of hospitalisations for exacerbations of COPD on health-related quality of life. *Respir Med* 2009;103:1201-8.
10. Hurst JR, Vestbo J, Anzueto A, Locantore N, Mullerova H, Tal-Singer R, et al. Susceptibility to exacerbation in chronic obstructive pulmonary disease. *N Engl J Med* 2010;363:1128-38.
11. Husebo GR, Bakke PS, Aanerud M, Hardie JA, Ueland T, Gronseth R, et al. Predictors of exacerbations in chronic obstructive pulmonary disease--results from the Bergen COPD cohort study. *PLoS One* 2014;9:e109721.
12. Kerkhof M, Freeman D, Jones R, Chisholm A, Price DB, Grp RE. Predicting frequent COPD exacerbations using primary care data. *Int J Chron Obstruct Pulmon Dis* 2015;10:2439-50.
13. Steenland K, Hu S, Walker J. All-cause and cause-specific mortality by socioeconomic status among employed persons in 27 US states, 1984-4997. *Am. J. Public Health* 2004;94:1037-42.
14. Pleasants RA, Ohar JA, Croft JB, Liu Y, Kraft M, Mannino DM, et al. Chronic Obstructive Pulmonary Disease and Asthma - Patient Characteristics and Health Impairment. *COPD* 2014;11:256-66.
15. Osman S, Ziegler C, Gibson R, Mahmood R, Moraros J. The Association between Risk Factors and Chronic Obstructive Pulmonary Disease in Canada: A Cross-sectional Study Using the 2014 Canadian Community Health Survey. *Int J Prev Med*2017;8:86.
16. Maas J, Verheij RA, Groenewegen PP, de Vries S, Spreeuwenberg P. Green space, urbanity, and health: how strong is the relation? *J Epidemiol Community Health* 2006;60:587-92.
17. Jakubowski B, Frumkin H. Environmental Metrics for Community Health Improvement. *Prev Chronic Dis* 2010;7:A76.

For peer review only

18. Galea S, Abdalla SM, Sturchio JL. Social determinants of health, data science, and decision-making: Forging a transdisciplinary synthesis. *PLoS Med* 2020;17:e1003174.
19. Dinov ID, Heavner B, Tang M, Glusman G, Chard K, Darcy M, et al. Predictive Big Data Analytics: A Study of Parkinson's Disease Using Large, Complex, Heterogeneous, Incongruent, Multi-Source and Incomplete Observations. *PLoS One* 2016;11:e0157077.
20. Tan SS, Gao G, Koch S. Big Data and Analytics in Healthcare. *Methods Inf Med* 2015;54:546-7.
21. Remington PL, Catlin BB, Gennuso KP. The County Health Rankings: rationale and methods. *Popul Health Metr* 2015;13:11.
22. Zakowska I, Kosiek K, Kowalczyk A, Grabowski J, Godycki-Cwirko M. Community determinants of COPD exacerbations in elderly patients in Poland: protocol for a retrospective Big Data observational cohort study. *BMJ Open* 2019;9:e030524.
23. Morgan AD, Herrett E, De Stavola BL, Smeeth L, Quint JK. COPD disease severity and the risk of venous thromboembolic events: a matched case-control study. *Int J Chron Obstruct Pulmon Dis* 2016;**11**:899-908.
24. GUS. Łódzkie Voivodship 2017 - Subregions, Powiats, Gminas. Statistical Office in Łódź. Statistical Yearbook of Łódzkie Voivodship 2017. GUS Publishing 2017 https://lodz.stat.gov.pl/en/publications/statistical-yearbook/lodzkie-voivodship-2017-subregions-powiats-gminas,2,19.html
25. Wamba SF, Akter S, Edwards A, Chopin G, Gnanzou D. How 'big data' can make big impact: Findings from a systematic review and a longitudinal case study. *Int J Prod Econ* 2015;165:234-46.
26. Lee J, Jung HM, Kim SK, Yoo KH, Jung KS, Lee SH, et al. Factors associated with chronic obstructive pulmonary disease exacerbation, based on big data analysis. *Sci. Rep* 2019;9:6679.
27. Rhee CK, Kim JW, Yoo KH, Jung KS. Prediction model of COPD acute exacerbation with big data by machine learning methods. Virtual Congress. 2020;4911 https://www.ers-education.org/lr/show-details/?idP=247196
28. Halbert RJ, Natoli JL, Gano A, Badamgarav E, Buist AS, Mannino DM. Global burden of COPD: systematic review and meta-analysis. *Eur Respir J* 2006;28:523-32.
29. Han MK, Postma D, Mannino DM, Giardino ND, Buist S, Curtis JL, et al. Gender and chronic obstructive pulmonary disease: why it matters. *Am J Respir Crit Care Med* 2007;176:1179-84.
30. Moretti AM, Gallone MS, Parisi D, Tafuri S, Germinario C. Gender differences and hospitalization for COPD: an analysis of data from Puglia (Italy). *Ital J Gender-Specific Med* 2015;1:66-72.
31. Lykkegaard J, dePont Christensen R, Davidsen JR, Stovring H, Andersen M, Sondergaard J. Trends in the lifetime risk of COPD exacerbation requiring hospitalisation. *Eur Respir J* 2013;42:964-71.
32. Eisner MD, Blanc PD, Omachi TA, Yelin EH, Sidney S, Katz PP, et al. Socioeconomic status, race and COPD health outcomes. *J Epidemiol Community Health* 2011;65:26-34.
33. van Doorslaer E, Masseria C, Koolman X. Inequalities in access to medical care by income in developed countries. *CMAJ* 200617;174:177-83.
34. Fong KC, Hart JE, James P. A Review of Epidemiologic Studies on Greenness and Health: Updated Literature Through 2017. *Curr Env Hlth Rep* 2018;5:77-87.
35. Crouse DL, Pinault L, Balram A, Hystad P, Peters PA, Chen H, et al. Urban greenness and mortality in Canada's largest cities: a national cohort study. *Lancet Planet Health* 2017;1:e289-e97.

For peer review only

36. Fan J, Guo Y, Cao Z, Cong S, Wang N, Lin H, et al. Neighborhood greenness associated with chronic obstructive pulmonary disease: A nationwide cross-sectional study in China. *Environ Int* 2020;144:106042.
37. Burkes RM, Gassett AJ, Ceppe AS, Anderson W, O'Neal WK, Woodruff PG, et al. Rural Residence and Chronic Obstructive Pulmonary Disease Exacerbations. Analysis of the SPIROMICS Cohort. *Ann Am Thorac Soc* 2018;15:808-16.
38. Hu G, Zhou Y, Tian J, Yao W, Li J, Li B, et al. Risk of COPD from exposure to biomass smoke: a metaanalysis. *Chest* 2010;138:20-31.
39. Perez-Padilla R, Regalado J, Vedal S, Pare P, Chapela R, Sansores R, et al. Exposure to biomass smoke and chronic airway disease in Mexican women. A case-control study. *Am J Respir Crit Care Med* 1996;154:701-6.

For peer review only

Supplementary: Table 1S Descriptive characteristics and univariate analysis of COPD exacerbations in cohort of COPD elderly patients and prevalence of COPD in PHC (Lodz province in 2016). (Raw data before PSM).

**Patient Characteristic (Before PSM)**	**COPD patients without exacerbations (n=14607)**	**COPD patients with exacerbations (n=1492)**	**Total (n=16099)**	**OR (95% CI), p-value #Univariate Logistic Regression.**
Patient level				
Demographic				
COPD Patients (Sum)(%) Total	14607 (90.73)	1492 (9.27)	16099 (100)	
Sex				
Male	7652(52.39)	886(59.38)	8538(53.03)	1.329(1.192-1.481)
Female	6955(47.61)	606(40.62)	7561(46.97)	1 Ref. Cat.
Age (years)				
<75-85)	5046(34.55)	600(40.21)	5646(35.07)	1.322(1.18-1.482)
<85+>	1587(10.86)	175(11.73)	1762(10.94)	1.226(1.031-1.459)
<65-75)	7974(54.59)	717(48.06)	8691(53.98)	1 Ref. Cat.
Patient’s place of residence in gmina (localization)				
1-urban	8971(61.42)	864(57.91)	9835(61.09)	0.840(0.728-0.971)
2-rural	3280(22.45)	358(23.99)	3638(22.6)	0.952(0.806-1.125)
3-urban-rural	2356(16.13)	270(18.1)	2626(16.31)	1 Ref. Cat.
Healthcare uses				
Specialization of physician working in PHC				
Family doctors	5483(37.54)	587(39.34)	6070(37.7)	1 Ref. Cat.
Other	9124(62.46)	905(60.66)	10029(62.3)	0.926(0.831-1.033)
Number of consultations in PHC^				
high (>12)	5309(36.35)	689(46.18)	5998(37.26)	1.277(1.063-1.535)
medium <5-12>	7783(53.28)	649(43.5)	8432(52.38)	0.820(0.682-0.986)

For peer review only

low (<5)	1515(10.37)	154(10.32)	1669(10.37)	1 Ref. Cat.
Number of COPD consultations in PHC^				
0	166 (1.14)	548 (36.73)	714 (4.44)	21.718 (17.658-26.712)
1	7701 (52.72)	286 (19.17)	7987 (49.61)	0.244 (0.208-0.288)
2	2930 (20.06)	191 (12.8)	3121 (19.39)	0.429 (0.357-0.516)
3	1514 (10.36)	118 (7.91)	1632 (10.14)	0.513 (0.412-0.638)
4+	2296 (15.72)	349 (23.39)	2645 (16.45)	1 Ref. Cat.
Gmina/Commune, post-codes level				
Socioeconomic Status				
Total personal income of residents per PHC post-codes (Tax Office)				
high (>21992,62)	3717 (25.45)	240 (16.09)	3957 (24.58)	0.573 (0.486-0.677)
medium <17612,68-21992,62>	7231 (49.5)	840 (56.3)	8071 (50.13)	1.032 (0.911-1.168)
low (<17612,68)	3659 (25.05)	412 (27.61)	4071 (25.29)	1 Ref. Cat.
Environmental factors				
Forest cover of the patient's place of residence in gmina (GUS)				
high (>17,10%)	3043 (20.83)	308 (20.64)	3351 (20.81)	0.791 (0.686-0.913)
medium <8,40%-17,10%>	6583 (45.07)	547 (36.66)	7130 (44.29)	0.65 (0.576-0.733)
low (<8,40%)	4981 (34.1)	637 (42.69)	5618 (34.9)	1 Ref. Cat.
Total	14607 (90.73)	1492 (9.27)	(100)	

^Pearson Correlation Coefficient=0,2468, p<0.0001 between "Number of consultations in PHC" and "Number of COPD consultations in PHC". CI=Confidence Interval, OR=Odds Ratio. #=Odds Ratio Estimates (Point Estimate). 95% Wald Confidence Limits. OR (95% CI). #Univariate analysis Univariate Logistic Regression. ##Univariate High-Performance Logistic Regression

For peer review only

Table 2S. Descriptive characteristics and local determinants of COPD exacerbations in cohort of COPD elderly patients (Lodz province, 2016; case-control). After PSM.

**Patient Characteristic (After PSM)**	**COPD patients without exacerbations Control (n=1492 pairs)**	**COPD patients with exacerbations Cases (n=1492 pairs)**	**Total (n=2984)**	**OR (95% CI) Univariate analysis****	**OR (95% CI)**
**Patient level**					
**Demographic**					
**COPD Patients (Sum)(%) Total**	1492 (50)	1492 (50)	2984 (100)		
**Sex (strata variable)**					
**Male**	886 (59.38)	886 (59.38)	1772 (59.38)	1 Ref. Cat^	Strata variable
**Female**	606 (40.62)	606 (40.62)	1212 (40.62)	1.000 (0.864-1.157)^	
**Age (years) (strata variable)**					
**<75-85)**	600(40.21)	600(40.21)	1200(40.21)	1.000 (0.788-1.269)^	Strata variable
**<85+>**	175(11.73)	175(11.73)	350(11.73)	1 Ref. cat.^	
**<65-75)**	717(48.06)	717(48.06)	1434(48.06)	1.000 (0.792-1.263)^	
**Patient's place of residence in gmina (localization)**					
**1-urban vs. 3**	862 (57.77)	864 (57.91)	1726 (57.84)	1.028 (0.848-1.246)	1.044 (0.673-1.620)
**2-rural vs. 3**	353 (23.66)	358 (23.99)	711 (23.83)	1.04 (0.833-1.3)	0.897 (0.630-1.277)
**3-urban-rural (Ref. Cat.)**	277 (18.57)	270 (18.1)	547 (18.33)	1 Ref. Cat.	1 Ref. Cat.
**Healthcare uses**					
**Specialization of physician working in PHC**					
**Family doctors vs. Other**	562(37.67)	587(39.34)	1149(38.51)	1.080(0.926-1.259)	1.076 (0.920-1.257)
**Other (Ref. Cat.)**	930(62.33)	905(60.66)	1835(61.49)	1 Ref. Cat.	1 Ref. Cat.
**Number of consultations in PHC^**					
**high (>12) vs. low (<5)**	558 (37.4)	689 (46.18)	1247 (41.79)	1.269 (0.982-1.639)	1.261 (0.974-1.633)

For peer review only

**medium <5-12> vs. low (<5)**	783 (52.48)	649 (43.5)	1432 (47.99)	0.831 (0.651-1.061)	0.823 (0.643-1.053)
**low (<5) (Ref. Cat.)**	151 (10.12)	154 (10.32)	305 (10.22)	1 Ref. Cat.	1 Ref. Cat.
**Number of COPD consultations in PHC^**					
**0 vs. 4+**	13 (0.87)	548 (36.73)	561 (18.8)	41.078 (18.064-93.413)	
**1 vs. 4+**	751 (50.34)	286 (19.17)	1037 (34.75)	0.288 (0.224-0.37)	
**2 vs. 4+**	299 (20.04)	191 (12.8)	490 (16.42)	0.488 (0.369-0.645)	
**3 vs. 4+**	185 (12.4)	118 (7.91)	303 (10.15)	0.528 (0.377-0.738)	
**4+ (Ref. Cat.)**	244 (16.35)	349 (23.39)	593 (19.87)	Ref. Cat.	
**Gmina/Commune, post-codes level**					
**Socioeconomic Status**					
**Total personal income of residents per PHC post-codes (Tax Office)**					
**high (>21056,52) vs. low (<17079,50)**	371 (24.87)	345 (23.12)	716 (23.99)	0.696 (0.489-0.990)	0.601 (0.385-0.939)
**medium <17079,50-21056,52> vs. low (<17079,50)**	759 (50.87)	768 (51.47)	1527 (51.17)	0.910 (0.722-1.147)	0.824 (0.581-1.169)
**low (<17079,50) (Ref. Cat.)**	362 (24.26)	379 (25.4)	741 (24.83)	1 Ref. Cat.	1 Ref. Cat.
**Environmental factors**					
**Forest cover of the patient's place of residence in gmina (GUS)**					
**high (>20,60%) vs. low (<7,00%)**	348 (23.32)	340 (22.79)	688 (23.06)	0.896 (0.648-1.24)	0.897 (0.605-1.331)
**medium <7,00%-20,60%> vs. low (<7,00%)**	776 (52.01)	776 (52.01)	1552 (52.01)	0.94 (0.688-1.285)	0.925 (0.648-1.322)
**low (<7,00%) (ref. Cat.)**	368 (24.66)	376 (25.2)	744 (24.93)	1 Ref. Cat.	1 Ref. Cat.
**Total**	1492 (50)	1492 (50)	2984 (100)		

CI=Confidence Interval, OR=Odds Ratio. #=Odds Ratio Estimates (Point Estimate). 95% Wald Confidence Limits. OR (95% CI).
Ref. cat.=reference category.
^- Sex and age were strata variables (the LOGISTIC Procedure, Logistic Regression).
**The LOGISTIC Procedure, Conditional Logistic Regression for Matched Pairs.

For peer review only

Descriptive characteristics of determinants

"Patient with ICD-10 code J44." - Determination whether J44 ICD-10 code is present in patients' medical records in PHC clinics

"Exacerbations" - "Hospitalization because of J44." - Determination of whether the patient was "hospitalized with the J44 code as a main reason for admission. (Main diagnosis checked at the hospitalization level and not at individual wards).

"Patient's place of residence in gmina" (localization) - based on patients' PHC clinic address with assigned post- code

Division into the type of communes according to the Central Statistical Office: 1-urban, 2-rural, 3-urban-rural (Ref. Cat.). GUS Kind of gminas name: https://bdl.stat.gov.pl/BDL/metadane/teryt/rodzaj

"Specialization of physician working in PHC": Family doctors, Other (Ref. Cat.) - Specialization of the physician working in PHC clinic within the Lodz province - family doctors, and other physicians (internal medicine specialist, pediatrician, physician without specialization). (NFZ database)

"Number of consultations in PHC": high (>12), medium <5-12>, low (<5) (Ref. Cat.) - The number of patient's consultations for any reason in PHC clinic in 2016

"Number of COPD consultations in PHC": 0, 1, 2, 3, 4+ (Ref. Cat.) – the number of patient's health care consultations with the main diagnosis J44in 2016

"Codes of diseases associated with COPD (PHC)" – codes of comorbidities in the medical record of a patient with COPD. in 2016

"Total personal income of residents per PHC post-codes" – (Tax Office): high (>21056,52 PLN), medium <17079,50 PLN -21056,52 PLN > , low (<17079,50 PLN) (Ref. Cat.)

"Forest cover of the patient's place of residence in gmina" (GUS): high (>20,60%), medium <7,00%-20,60%>, low (<7,00%) (ref. Cat.) in 2016

"The total cost of STAC" - The NFZ costs for all hospital services in 2016 for a given patient, regardless of the main diagnosis

For peer review only

"Total cost of drug reimbursement" - The NFZ costs for all prescribed medications in 2016 for a given patient

"Total NFZ cost" - The NFZ cost – (as sum of costs of PHC, ASC, hospitalization, drug reimbursement and other) in 2016 per patient, regardless of the main diagnosis

For peer review only

APPENDIX

Table 1A. Descriptive characteristics of cohort of COPD elderly patients within Lodz province (Raw data before PSM, n=16 099) and prevalence of COPD, including prevalence of COPD exacerbations (n=17 240, including deaths).

Characteristic	COPD without exacerbations (n=14 607)	COPD with exacerbations (n=1 492)	Total (n=16 099)	Odds Ratio Estimates Point Estimate 95% Wald Confidence Limits OR [95% CI] - Univariate analysis# HP Logistic Regression
**Total J44 Patients Sum (%)**	14 607 (90.73)	1 492 (9.27)	16 099	
**Exacerbation number**	0	1	Total	Data only for the group with exacerbations.
**1**	0 (0)	1 294 (86.73)	1 294 (8.04)	
**2**	0 (0)	148 (9.92)	148 (0.92)	
**3**	0 (0)	32 (2.14)	32 (0.2)	
**4**	0 (0)	11 (0.74)	11 (0.07)	
**5**	0 (0)	5 (0.34)	5 (0.03)	
**6**	0 (0)	2 (0.13)	2 (0.01)	
**Exacerbation number**				
**(min. - max.)**	0	(1 - 6)	(1 - 6)	
**Missing**	14 607	0	14 607	Data only for the group with exacerbations.
**Total (including Missing)**	14 607 (90.73)	1 492 (9.27)	16 099 (100)	
**Age of the patient 65+Median(q1 - q3)**	73 (68 - 80)	75 (69 - 81)	74 (69 - 80)	1.016 [1.009, 1.023]

For peer review only - http://bmjopen.bmj.com/site/about/guidelines.xhtml

				**Continuous var.
**Number of COPD consultations (PHC)**				
**(min. - max.)**	(0 - 24)	(0 - 20)	(0 - 24)	
**Number of consultations in PHC (min. - max.) **Continuous var.**	(0 - 78)	(0 - 59)	(0 - 78)	
**Drugs## Number of packages Avg±SD**				
**R03AC***	5±5.7	6±6.95	5±5.86	1.036 [1.028,1.044] **Continuous var.
**R03AK***	2±3.83	2±3.99	2±3.84	1.022 [1.008,1.035] **Continuous var.
**R03AL***	1±(5.39	3±8.99	1±5.89	1.041 [1.034, 1.048] **Continuous var.
**R03BA***	2±4.51	4±5.72	2±4.66	1.043 [1.034, 1.053] **Continuous var.
**R03BB***	5±6.11	6±7.3	5±6.27	1.037 [1.029, 1.044] **Continuous var.
**R03DA***	3±4.99	4±5.12	3±5.02	1.038 [1.028, 1.048] **Continuous var.
**R03DC***	0±1.68	0±1.52	0±1.67	0.985 [0.952, 1.020] **Continuous var.
**COST OF MEDICINES (Drugs) [%]**	Percent of median cost per COPD with exacerbations/median cost per COPD without exacerbations [%]			

For peer review only - http://bmjopen.bmj.com/site/about/guidelines.xhtml

**Total Fund Cost (Drugs) in [%]NMiss=1906 (11.84%)**	144.69%			HP LR estimate= 0.000316 **Continuous var.
**Income per post code NMiss=99 Difference between median COPD with exacerbations and median without exacerbations [PLN]**	584.02 PLN			**Continuous
**Did the patient die in 2016?**				
**No**	14 607 (94.51)	1 492 (83.63)	16 099 (93.38)	
**Yes**	849 (5.49)	292 (16.37)	1 141 (6.62)	
**Total**	15 456 (89.65)	1 784 (10.35)	17 240 (100)	
**Physicians'characteristics in PHC in Lodz Voivodeship**				
**Physicians'Age**				
**Median(q1-q3)**	52 (43.61)			
**Number**	2 465			
**Physician's sex**				
**Women=0**	1 674 (67.91)			
**Men=1**	791 (32.09)			
**Total**	2 465 (100)			
**Physician's spacialization**				
**Other=0**	1 838 (74.56)			
**Family doctor=1**	627 (25.44)			
**Total**	2 465 (100)			
**Total Physicians in Lodz voivodeship****	2 465 (100)			
**Prevalence**				
**Prevalence of total of COPD n=17240 among 65+ n=472 314 in Lodz Voivodeship, in PHC in 2016 (including deaths) = 3.65%, 95%CI (3.60-3.70) [%].**				
**Prevalence of total of exacerbations n=1784 among COPD 65+ n=17240 in Lodz Voivodeship, in PHC in 2016 (including death)= 10.35%, 95%CI (9.89-10.80) [%].**				

For peer review only

For peer review only - http://bmjopen.bmj.com/site/about/guidelines.xhtml

Patient Characteristic	COPD without exacerbations (n=15 456)	COPD with exacerbations (n=1 784)	Total (n=17 240)	OR (95% CI) - Univariate analysis Logistic Regression
**Total Patients COPD 65+ including deaths**	15 456 (89.65)	1 784 (10.35)	17 240 (100)	
**Sex**				
**Female**	7 256 (46.95)	708 (39.69)	7 964 (46.19)	F Ref. Cat.
**Male**	8 200 (53.05)	1 076 (60.31)	9 276 (53.81)	1.345 [1.217,1.486]
**Total**	15 456 (89.65)	1 784 (10.35)	17 240 (100)	

*Data are presented as n (%), mean ±SD or median (q1 - q3)..
#Crude, Univariate Model, OR (95% CI), Odds Ratio with 95% Wald, Confidence Limits using proc logistic, proc hplogistic.
**Lodz Voivodeship known as Lodz province (Wikipedia): https://en.wikipedia.org/wiki/%C5%81%C3%B3d%C5%BA_Voivodeship
ATC group - Anatomical Therapeutic Chemical (ATC) Classification
R03 - drugs for obstructive airway diseases
R03A - adrenergics, inhalants:
*R03AC - Selective beta-2-adrenoreceptor agonists
*R03AK - Adrenergics in combination with corticosteroids or other drugs, excl. anticholinergics
*R03AL - Adrenergics in combination with anticholinergics incl. triple combinations with corticosteroids
R03B - other drugs for obstructive airway diseases, inhalants:
*R03BA – Glucocorticoids
*R03BB - Anticholinergics
R03D - other systemic drugs for obstructive airway diseases:
*R03DA - Xanthines
*R03DC - Leukotriene receptor antagonists
https://indeks.mp.pl/leki/find.php?kind=atc&subatc=1&id=R03
- NMiss=1906 (11.84%) of missing data per each of the 7 variables with drugs:*R03AC, *R03AK, *R03AL, *R03BA, *R03BB, *R03DA, *R03DC.

For peer review only - http://bmjopen.bmj.com/site/about/guidelines.xhtml

Table 2A Characteristic of COPD patients with and without exacerbations (After PSM).

Characteristic	COPD patients without exacerbations (n=1 492 pairs) Control (J44) n(%)	COPD patients with exacerbations (n=1 492 pairs) Case (Hospitalized due to J44) n(%)	Total patients (n=2 984) n(%)	OR (95% Wald Confidence Limits). (Point Estimate.) OR (95% CI) - Univariate analysis# Paired LR
**Total**	1 492 (50)	1 492 (50)	2 984 (100)	
**Exacerbation number**				Data only for the group with exacerbations.
**1**	0 (0)	1 294 (86.73)	1 294 (8.04)	
**2**	0 (0)	148 (9.92)	148 (0.92)	
**3**	0 (0)	32 (2.14)	32 (0.2)	
**4**	0 (0)	11 (0.74)	11 (0.07)	
**5**	0 (0)	5 (0.34)	5 (0.03)	
**6**	0 (0)	2 (0.13)	2 (0.01)	
**Total**	1 492 (50)	1 492 (50)	2 984 (100)	
**Age of the patient 65+ (years) Median(q1-q3)**	75 (69.00- 81.00)	75 (69.00- 81.00)	75 (69.00- 81.00)	PSM NS LR No Effect after PSM
**Number of refunded drugs packages (ATC groups) (Missing 7.37%) Avg±SD)**				
**R03AC***	4.65±±5.63	6.08±6.95	5.4±6.39	1.037 [1.023,1.051]
**R03AK***	2.01±3.9	2.46±3.99	2.25±3.95	1.028 [1.007,1.049]
**R03AL***	0.95±4.18	3.41±8.99	2.24±7.21	1.087 [1.064,1.11]
**R03BA***	2.38±4.25	3.55±5.72	3±5.1	1.051 [1.033,1.069]
**R03BB***	4.47±5.83	6.38±7.3	5.46±6.71	1.049 [1.035,1.064]
**R03DA***	3.06±5.12	4.01±5.12	3.56±5.14	1.038 [1.022,1.055]
**R03DC***	0.25±1.45	0.28±1.52	0.26±1.49	0.997 [0.945,1.051]
**Total NFZ cost for, all current and settled services in 2016, regardless of the main diagnosis and reimbursement of drugs from prescriptions completed in 2016**				
**high (>21 056.52)**	186 (12.47)	560 (37.53)	746 (25)	42.804 [28.145,65.097]
**medium <2 497.62-8 264.65>**	618 (41.42)	875 (58.65)	1 493 (50.03)	18.503 [12.627,27.115]
**low (<2 497.62)**	688 (46.11)	57 (3.82)	745 (24.97)	1 Ref. Cat.
**Total**	1 492 (50)	1 492 (50)	2 984 (100)	

*Data are presented as n (%), mean ±SD or median (q1q3).
IQR – interquartile range

For peer review only - http://bmjopen.bmj.com/site/about/guidelines.xhtml

Table 3A Costs for the raw data (Raw data before PSM, n=16 099) of COPD patients with and without exacerbations.
Cost indicators (%) were calculated as contribution of patient costs with exacerbations (or without) among the cost for all COPD patients.

Characteristic Costs n(%) N=16099	COPD without exacerbations (n=14 607)	COPD with exacerbations (n=1 492)	Total (n=16 099)	p-value - Univariate analysis# HP Logistic Regression
**Total J44 Patients Sum (%)**	14 607 (90.73)	1 492 (9.27)	16 099	
**Total stationary costs (%)***				
**high**	3189 (21,83)	838 (56,17)	4027 (25,01)	<.0001
**medium**	1756 (12,02)	642 (43,03)	2398 (14,9)	<.0001
**low**	9662 (66,15)	12 (0,8)	9674 (60,09)	Ref. Cat.
**Total**	14607 (90,73)	1492 (9,27)	16099 (100)	
**Total cost of reimbursed drugs (%)***				
**high**	3434 (23,51)	590 (39,54)	4024 (25)	<.0001
**medium**	7363 (50,41)	687 (46,05)	8050 (50)	<.0001
**low**	3810 (26,08)	215 (14,41)	4025 (25)	Ref. Cat.
**Total**	14607 (90,73)	1492 (9,27)	16099 (100)	
**TOTAL NFZ cost (%)***				
**high**	3191 (21,85)	833 (55,83)	4024 (25)	<.0001
**medium**	7398 (50,65)	653 (43,77)	8051 (50,01)	<.0001
**low**	4018 (27,51)	6 (0,4)	4024 (25)	Ref. Cat.
**Total**	14607 (90,73)	1492 (9,27)	16099 (100)	

*Costs were calculated as the proportion of costs per patient in the mean of the COPD patient population [%], and categorized by lower (q1) and upper (q3) quartiles.
q1-Lower Quartile; q3-Upper Quartile, CL-Confidence Limit, HP LR-High Performance Logistic Regression.
N=16099
Total stationary costs (%) q1=0.00 q3=101.72
Total cost of reimbursed drugs (%) q1=34.75 q3=141.10
TOTAL NFZ cost (%) q1=28.71 q3=111.69

For peer review only - http://bmjopen.bmj.com/site/about/guidelines.xhtml

Table 3B Costs for the data after PSM transformation (N=1492 pairs) of COPD patients with and without exacerbations.

Characteristic Costs n(%) N=1492 pairs	COPD patients without exacerbations (n=1492 pairs) n(%)	COPD patients with exacerbations exacerbations (n=1492 pairs) n(%)	TotalAll patients (n=2984) n(%)	p-value - Univariate analysis Conditional Logistic Regression for Matched Pairs
**Total J44 Patients Sum (%)**	1492 (50)	1492 (50)	2984 (100)	
**Total stationary costs (%)***				
**high**	168 (11,26)	564 (37,8)	732 (24,53)	<.0001
**medium**	332 (22,25)	916 (61,39)	1248 (41,82)	<.0001
**low**	992 (66,49)	12 (0,8)	1004 (33,65)	Ref. Cat.
**Total**	1492 (50)	1492 (50)	2984 (100)	
**Total cost of reimbursed drugs (%)***				
**high**	270 (18,1)	476 (31,9)	746 (25)	<.0001
**medium**	748 (50,13)	744 (49,87)	1492 (50)	<.0001
**low**	474 (31,77)	272 (18,23)	746 (25)	Ref. Cat.
**Total**	1492 (50)	1492 (50)	2984 (100)	
**TOTAL NFZ cost (%)***				
**high**	186 (12,47)	560 (37,53)	746 (25)	<.0001
**medium**	618 (41,42)	875 (58,65)	1493 (50,03)	<.0001
**low**	688 (46,11)	57 (3,82)	745 (24,97)	Ref. Cat.
**Total**	1492 (50)	1492 (50)	2984 (100)	

*Costs were calculated as the proportion of costs per patient in the mean of the COPD patient population [%], and categorized by lower (q1) and upper (q3) quartiles.
q1-Lower Quartile; q3-Upper Quartile, CL-Confidence Limit.
N=1492 pairs
Total stationary costs (%) q1=0.00 q3=111.56
Total cost of reimbursed drugs (%) q1=37.19 q3=142.19
TOTAL NFZ cost (%) q1=34.23 q3=113.27

For peer review only - http://bmjopen.bmj.com/site/about/guidelines.xhtml

Big Data Quality (Big Data Cleansing) Results: for Medical (NFZ) and Business (Tax Office) Data.

Files were obtained from Big Data databases (NFZ, Tax Office and GUS). In total, data for n = 472,314 cohorts of 65+ patients registered in primary health care (PHC) clinics were obtained from electronic medical records (EHR) of NFZ in the Lodz province. In total, n = 439,577 inhabitants 65+ living in the Lodz province were obtained from the databases of 28 Tax Offices located in the Lodz province. Variables for n = 177 communes of the Lodz province, which, according to the literature review, were related to exacerbations were collected from the GUS databases. Patient data from NFZ showed that the identifier has no duplicates. "Codes of comorbidities with COPD" had 98.8% of missing data (NFZ) out of 472 314 in total 65+ patients in PHC in Lodz province in 2016, and the variable "ICD9 procedures for other stays" had 98.1% of missing data (NFZ). Only 0.1% was missing in the variable "Postal code for 65+ residents" in Tax Office databases. Data on air quality for the group "Air pollutant emission from particular nuisance plants (G216)" consisting of 6 subgroups were not available at the level of data sharing for gmina level for 2016 in the GUS database (accessed on November 3, 2021) .

Propensity score matching (PSM) - before and after - overlay histograms age, gender

Fig. 1 Histogram for the age before PSM.

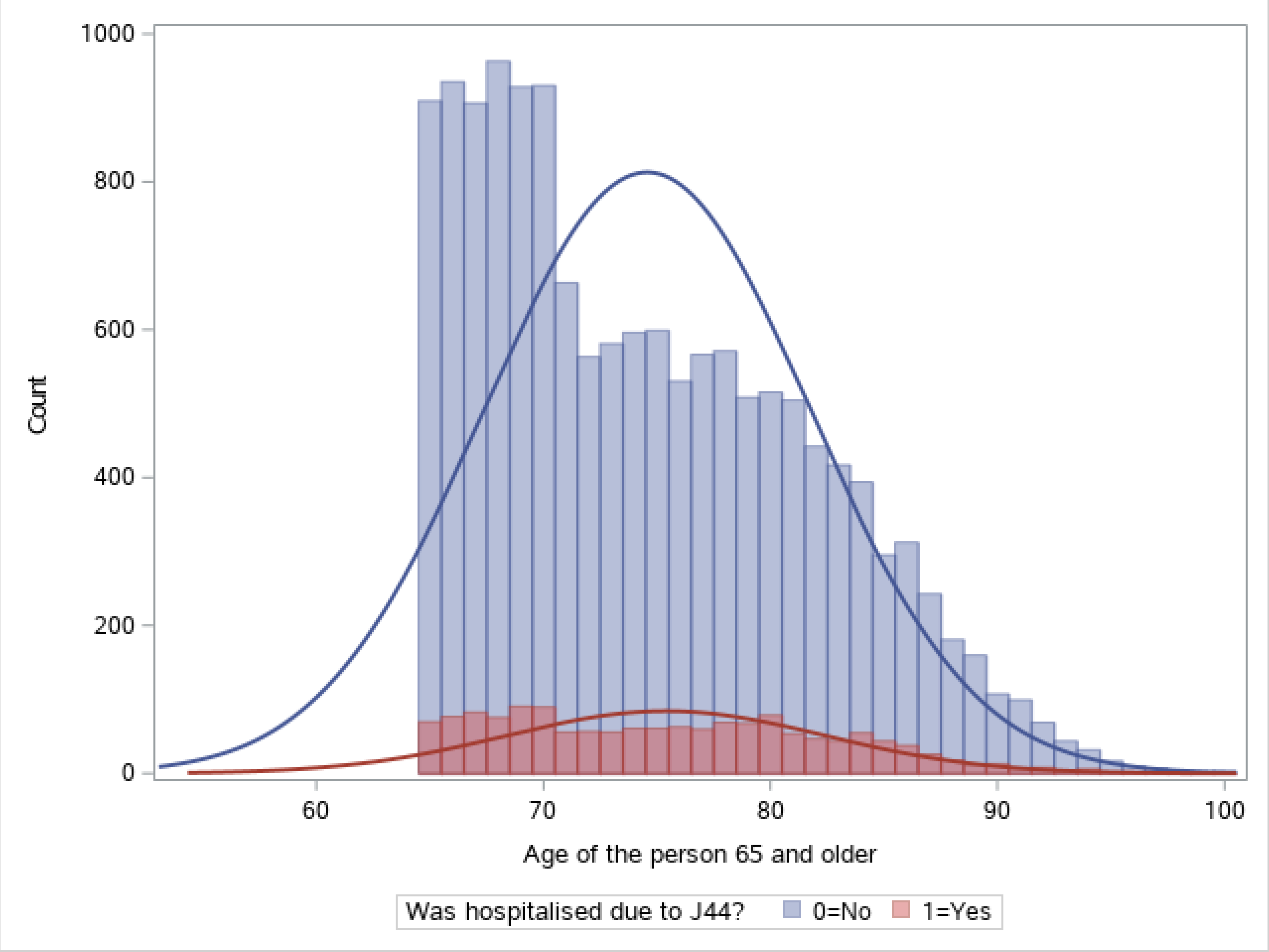

For peer review only - http://bmjopen.bmj.com/site/about/guidelines.xhtml

Fig. 2 Histogram for the age after PSM.

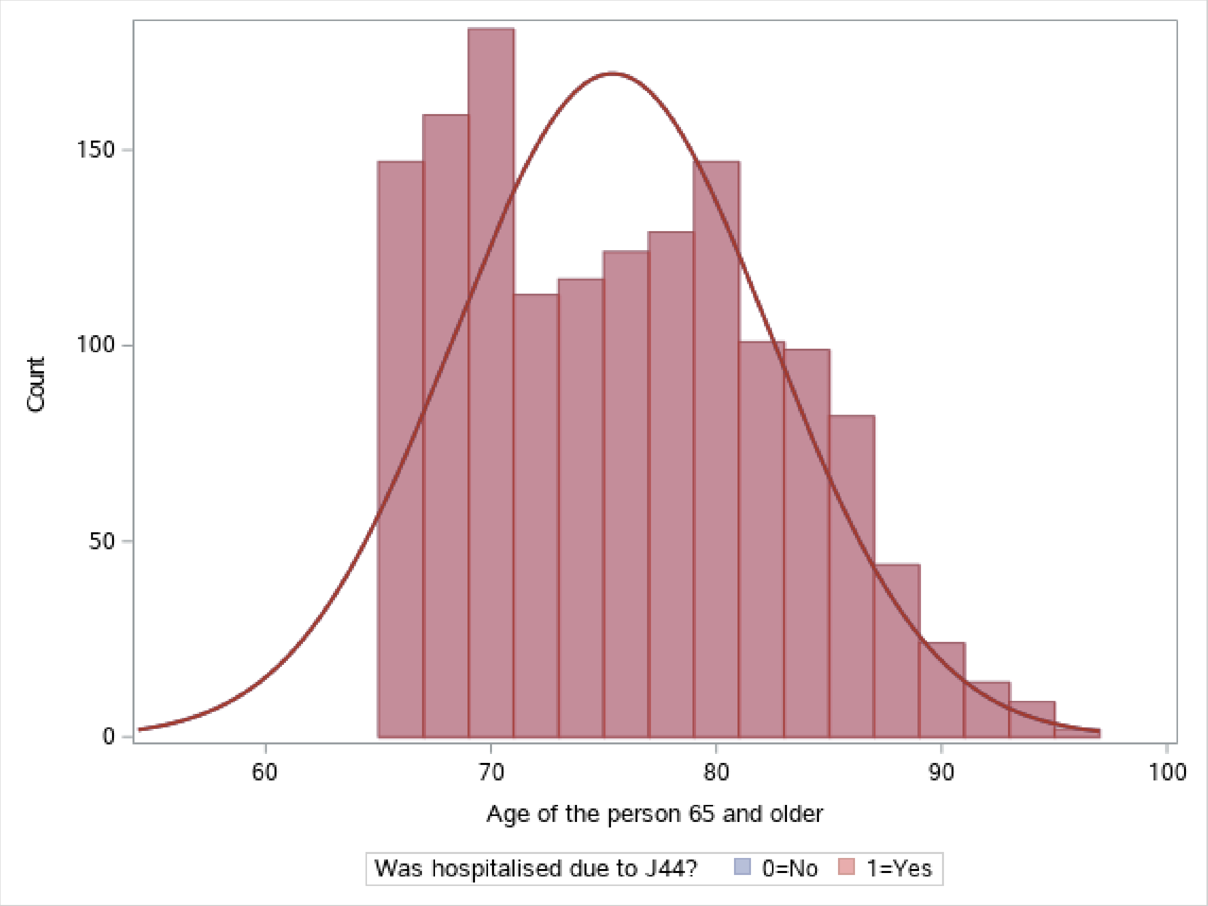

For peer review only - http://bmjopen.bmj.com/site/about/guidelines.xhtml

Fig. 3 Histogram for the gender before PSM.

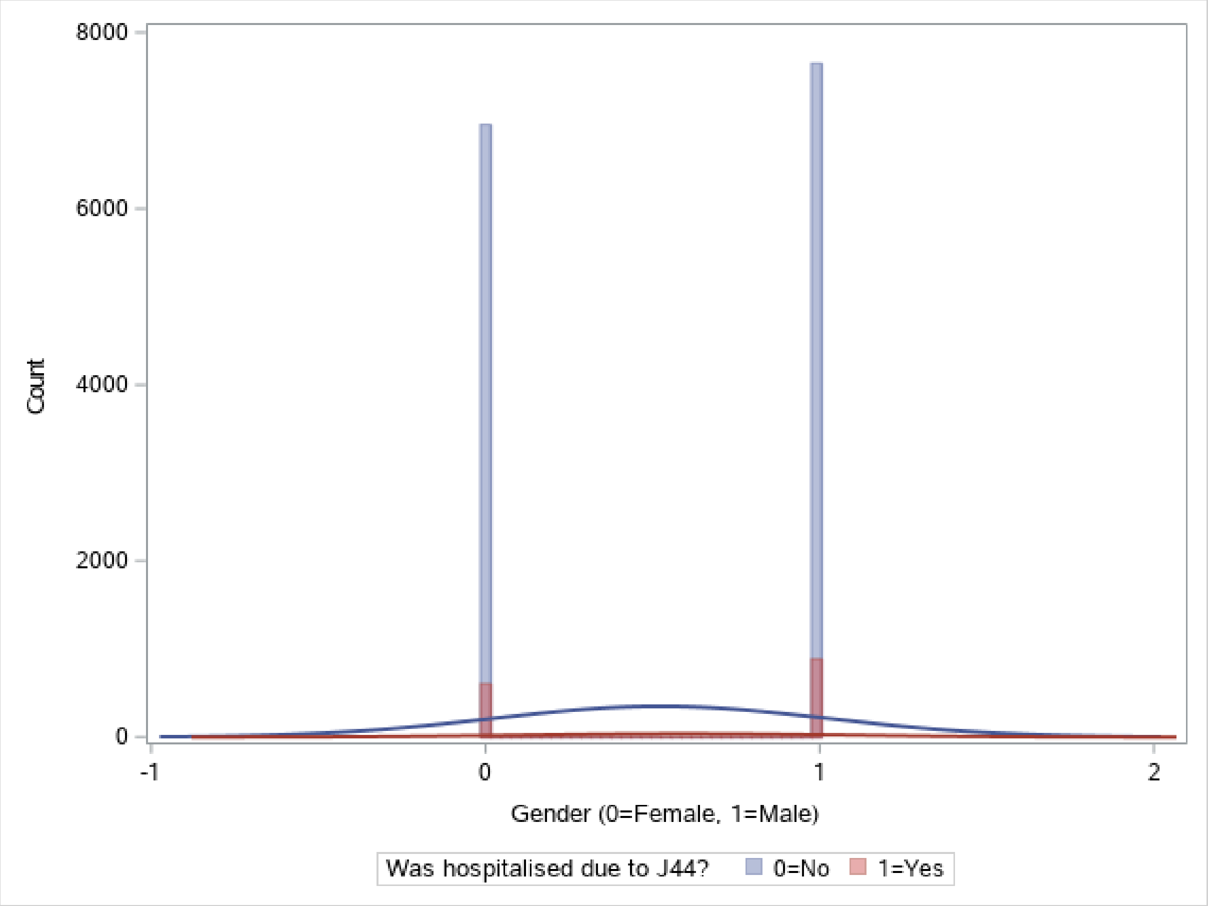

For peer review only - http://bmjopen.bmj.com/site/about/guidelines.xhtml

Fig. 4 Histogram for the gender after PSM.

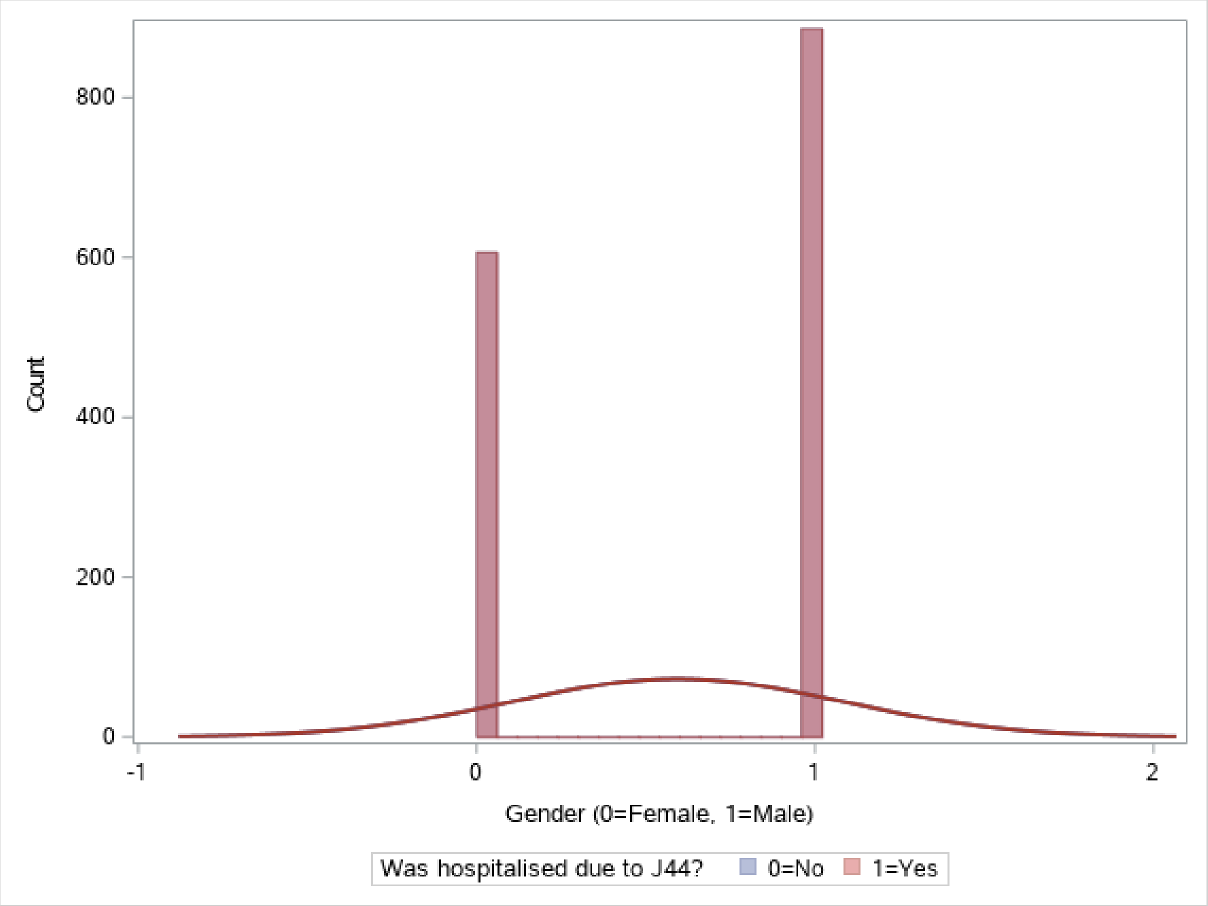

For peer review only - http://bmjopen.bmj.com/site/about/guidelines.xhtml

STROBE Statement—checklist of items that should be included in reports of observational studies

Title: Community determinants of COPD exacerbations in elderly patients in Lodz Province, Poland: a retrospective observational Big Data cohort study

	Item No	Recommendation	Page No
**Title and abstract**	1	(*a*) Indicate the study's design with a commonly used term in the title or the abstract	~~1-3~~44-46
		(*b*) Provide in the abstract an informative and balanced summary of what was done and what was found	~~1-4~~46
**Introduction**			
Background/rationale	2	Explain the scientific background and rationale for the investigation being reported	~~4-6~~47-49
Objectives	3	State specific objectives, including any prespecified hypotheses	~~6~~47
**Methods**			
Study design	4	Present key elements of study design early in the paper	~~5-8~~49-50
Setting	5	Describe the setting, locations, and relevant dates, including periods of recruitment, exposure, follow-up, and data collection	49-50~~5-6~~
Participants	6	(*a*) *Cohort study*—Give the eligibility criteria, and the sources and methods of selection of participants. Describe methods of follow-up<br>*Case-control study*—Give the eligibility criteria, and the sources and methods of case ascertainment and control selection. Give the rationale for the choice of cases and controls<br>*Cross-sectional study*—Give the eligibility criteria, and the sources and methods of selection of participants	~~6-7~~50
		(*b*) *Cohort study*—For matched studies, give matching criteria and number of exposed and unexposed<br>*Case-control study*—For matched studies, give matching criteria and the number of controls per case	~~7~~51
Variables	7	Clearly define all outcomes, exposures, predictors, potential confounders, and effect modifiers. Give diagnostic criteria, if applicable	~~6,8~~51-52
Data sources/ measurement	8*	For each variable of interest, give sources of data and details of methods of assessment (measurement). Describe comparability of assessment methods if there is more than one group	~~6-7~~49-50
Bias	9	Describe any efforts to address potential sources of bias	447, ~~17~~61-62
Study size	10	Explain how the study size was arrived at	~~8-9~~52
Quantitative variables	11	Explain how quantitative variables were handled in the analyses. If applicable, describe which groupings were chosen and why	~~8~~49-52
Statistical methods	12	(*a*) Describe all statistical methods, including those used to control for confounding	~~8~~51-52
		(*b*) Describe any methods used to examine subgroups and interactions	-
		(*c*) Explain how missing data were addressed	~~8~~51
		(*d*) *Cohort study*—If applicable, explain how loss to follow-up was addressed	~~8~~51

For peer review only - http://bmjopen.bmj.com/site/about/guidelines.xhtml

		Case-control study—If applicable, explain how matching of cases and controls was addressed<br>*Cross-sectional study*—If applicable, describe analytical methods taking account of sampling strategy	51-52
		(*e*) Describe any sensitivity analyses	-
**Results**			
Participants	13*	(a) Report numbers of individuals at each stage of study—eg numbers potentially eligible, examined for eligibility, confirmed eligible, included in the study, completing follow-up, and analysed	~~9~~52-53
		(b) Give reasons for non-participation at each stage	-
		(c) Consider use of a flow diagram	-
Descriptive data	14*	(a) Give characteristics of study participants (eg demographic, clinical, social) and information on exposures and potential confounders	~~9-14~~53-57, 64-65, Supplementary 70-73, APPENDIX Pages 76-81
		(b) Indicate number of participants with missing data for each variable of interest	APPENDIX Pages ~~9~~1-5, 9
		(c) *Cohort study*—Summarise follow-up time (eg, average and total amount)	~~9-12~~52-55
Outcome data	15*	*Cohort study*—Report numbers of outcome events or summary measures over time	52-55~~9-12~~
		Case-control study—Report numbers in each exposure category, or summary measures of exposure	55-58~~12-14~~
		Cross-sectional study—Report numbers of outcome events or summary measures	55-58~~12-14~~
Main results	16	(*a*) Give unadjusted estimates and, if applicable, confounder-adjusted estimates and their precision (eg, 95% confidence interval). Make clear which confounders were adjusted for and why they were included	52-53, 57-58, and 62~~14~~
		(*b*) Report category boundaries when continuous variables were categorized	64-65, Supplementary 70-74, ~~14~~APPENDIX Page 76-83
		(*c*) If relevant, consider translating estimates of relative risk into absolute risk for a meaningful time period	-
Other analyses	17	Report other analyses done—eg analyses of subgroups and interactions, and sensitivity analyses	-
**Discussion**			
Key results	18	Summarise key results with reference to study objectives	~~14-17~~60
Limitations	19	Discuss limitations of the study, taking into account sources of potential bias or imprecision. Discuss both direction and magnitude of any potential bias	~~4~~61
Interpretation	20	Give a cautious overall interpretation of results considering objectives, limitations, multiplicity of analyses, results from similar studies, and other relevant evidence	~~14-17~~58-61

For peer review only - http://bmjopen.bmj.com/site/about/guidelines.xhtml

For peer review only

Generalisability	21	Discuss the generalisability (external validity) of the study results	~~17~~62
**Other information**			
Funding	22	Give the source of funding and the role of the funders for the present study and, if applicable, for the original study on which the present article is based	~~18~~62

For peer review only

For peer review only - http://bmjopen.bmj.com/site/about/guidelines.xhtml

BMJ Open

Community determinants of COPD exacerbations in elderly patients in Lodz province, Poland: a retrospective observational Big Data cohort study

Journal:	*BMJ Open*
Manuscript ID	bmjopen-2021-060247.R2
Article Type:	Original research
Date Submitted by the Author:	15-Sep-2022
Complete List of Authors:	Kowalczyk, Anna; Medical University of Lodz, Centre for Family and Community Medicine, Faculty of Medical Sciences<br>Kosiek, Katarzyna; Family Doctors' Clinic<br>Godycki-Cwirko , Maciek; Medical University of Lodz, Centre for Family and Community Medicine, Faculty of Medical Sciences<br>Zakowska, Izabela; Medical University of Lodz, 1. Centre for Family and Community Medicine, the Faculty of Health Sciences
**Primary Subject Heading**:	Respiratory medicine
Secondary Subject Heading:	Respiratory medicine, Public health, General practice / Family practice
Keywords:	Respiratory infections < THORACIC MEDICINE, PUBLIC HEALTH, PRIMARY CARE

SCHOLARONE™
Manuscripts

I, the Submitting Author has the right to grant and does grant on behalf of all authors of the Work (as defined in the below author licence), an exclusive licence and/or a non-exclusive licence for contributions from authors who are: i) UK Crown employees; ii) where BMJ has agreed a CC-BY licence shall apply, and/or iii) in accordance with the terms applicable for US Federal Government officers or employees acting as part of their official duties; on a worldwide, perpetual, irrevocable, royalty-free basis to BMJ Publishing Group Ltd ("BMJ") its licensees and where the relevant Journal is co-owned by BMJ to the co-owners of the Journal, to publish the Work in this journal and any other BMJ products and to exploit all rights, as set out in our licence.

The Submitting Author accepts and understands that any supply made under these terms is made by BMJ to the Submitting Author unless you are acting as an employee on behalf of your employer or a postgraduate student of an affiliated institution which is paying any applicable article publishing charge ("APC") for Open Access articles. Where the Submitting Author wishes to make the Work available on an Open Access basis (and intends to pay the relevant APC), the terms of reuse of such Open Access shall be governed by a Creative Commons licence – details of these licences and which Creative Commons licence will apply to this Work are set out in our licence referred to above.

Other than as permitted in any relevant BMJ Author's Self Archiving Policies, I confirm this Work has not been accepted for publication elsewhere, is not being considered for publication elsewhere and does not duplicate material already published. I confirm all authors consent to publication of this Work and authorise the granting of this licence.

For peer review only

Title page

Community determinants of COPD exacerbations in elderly patients in Lodz province, Poland: a retrospective observational Big Data cohort study

Authors:

Anna Kowalczyk[1], Katarzyna Kosiek[2], Maciek Godycki-Cwirko[1], Izabela Zakowska[1]

1. Centre for Family and Community Medicine, the Faculty of Health Sciences, the Medical University of Lodz, Poland
2. Family Doctors' Clinic, Lodz, Poland.

Corresponding author:

Izabela Zakowska

Centre for Family and Community Medicine, Faculty of Medical Sciences,

Medical University of Lodz,

Pomorska 251 Street, building A2, room 203, 92-213 Lodz, Poland

izabela.zakowska@umed.lodz.pl

tel.: +48 42 272 53 77

Key words: COPD, Big Data, Exacerbations, Primary Health Care, Electronic Health Records.

Word count: 3874

For peer review only

Abbreviations:
65+ - patients 65 years old and older
AECOPD – Acute Exacerbations of chronic obstructive pulmonary disease
ASC – Ambulatory Specialists Care
ATC - Anatomical-therapeutic-chemical classification
COPD - Chronic Obstructive Pulmonary Disease
EHRs - electronic health records
Gmina-administrative part of province
GUS - National Statistics Center (Statistics Poland)
ICD-10 - International Classification of Diseases
NFZ - National Health Fund
PHC – Primary Health Care
PSM – Propensity Score Matching
SES - socioeconomic status
SOR - Hospital Emergency Department
US - Tax Office (Revenue Administration Regional Office in Lodz)
TERYT - Registry identifiers (GUS)
ATC group - Anatomical Therapeutic Chemical (ATC) Classification
R03 - drugs for obstructive airway diseases
R03A - adrenergics, inhalants:
*R03AC - Selective beta-2-adrenoreceptor agonists
*R03AK - Adrenergics in combination with corticosteroids or other drugs, excl. anticholinergics
*R03AL - Adrenergics in combination with anticholinergics incl. triple combinations with corticosteroids
R03B - other drugs for obstructive airway diseases, inhalants:
*R03BA – Glucocorticoids
*R03BB - Anticholinergics
R03D - other systemic drugs for obstructive airway diseases:
*R03DA - Xanthines
*R03DC - Leukotriene receptor antagonists

ABSTRACT

Objectives To evaluate the prevalence and identify demographic, economic and environmental local community determinants of COPD exacerbations in elderly in primary care using Big Data approach.
Design Retrospective observational case-control study. based on Big Data from the National Health Found, Tax Office and National Statistics Center databases in 2016.
Setting Primary care clinics in the Lodz province in Poland.
Participants 472 314 patients aged 65 and older in primary care including 17 240 COPD patients, and 1784 with exacerbations (including deaths).
Outcome measures Exacerbations with demographic, economic and environmental local community determinants were retrieved. Conditional logistic regression for matched pairs was used to evaluate the local community determinants of COPD exacerbations among COPD patients.
Results The overall prevalence of COPD in the population of elderly patients registered in PHC clinics in Lodz province in 2016 was 3.65%, 95%CI (3.60-3.70) [%] and the prevalence of exacerbations was 10.35%, 95%CI (9.89-10.80) [%]. The high number of consultations in primary care clinics was associated with higher risk of COPD exacerbations (p=0.0687).

For peer review only

High-income patients were less likely to have exacerbations than low-income patients (high vs low OR = 0.601, 95% CI (0.385-0.939)). The specialization of the primary care physician did not have an effect on exacerbations (OR = 1.076, 95% CI (0.920-1.257)). Neither the forest cover per gmina (high vs low OR = 0.897, 95% CI (0.605-1.331); medium vs low OR = 0.925, 95% CI (0.648-1.322)), nor location of gmina (urban vs. urban-rural OR = 1.044; 95% CI (0.673-1.620)), (rural vs. urban-rural OR = 0.897, 95% CI (0.630-1.277)) appears to influence COPD exacerbations.

Conclusions Big Data statistical analysis facilitated the evaluation of the prevalence and determinants of COPD exacerbation in the elderly residents of Lodz province, Poland.
Modification of identified local community determinants may potentially decrease the number of exacerbations in COPD elderly patients.

For peer review only

Strengths and limitations

- Retrieving knowledge from three relevant community big data bases (National Health Found, Tax Office and National Statistics Center).
- Evaluation of prevalence of COPD and determinants of COPD exacerbations in elderly primary care patients in Lodz province in Poland.
- Its main limitation was the lack of standardization in the method of recording exacerbations between primary care, accident and emergency departments and hospital wards.
- No data existed for some parameters, such as air quality for gaseous and dust pollutants, at the commune level for examined period.

INTRODUCTION

Chronic obstructive pulmonary disease (COPD) is the third leading cause of death worldwide, causing 3.23 million deaths in 2019. [1] Despite the great effort directed at prevention and treatment guidelines, the prevalence continues to increase and is now estimated to range from 4% to 10% worldwide; this value varies considerably across the European union, i.e. from 1.26% to 13.87%, partly because of the differences in the definitions used. [2-4] In Poland, a medium-sized country with population of about 38 million inhabitants, studies suggest a COPD prevalence of 11% in the cities of Warsaw and Lublin; however, data is scarce in other regions. [5]

COPD is a progressive disease characterized by exacerbations, i.e. an acute worsening of the respiratory symptoms which may lead to hospitalization [6-8]. In addition, patients with stable COPD often have acute exacerbations (AECOPD) due to risk factors, comorbidities, poor treatment adherence and insufficient treatment. [9] Frequent exacerbations, defined as at least

For peer review only

two episodes per year, are considered a marker of increased burden and reduced survival in clinical epidemiology studies. [10]

Before such exacerbations can be effectively prevented, there is a need to identify the factors contributing to them. To date, most studies have focused on factors specific to COPD patients themselves, rather than on external factors. [11, 12] However, it is possible that Socioeconomic status (SES) may play a role. SES is a complex assessment of a person's economic and social position in society based on several factors including income, occupation, home and neighborhood environment, and educational attainment. [13]

COPD appears to have a stronger socioeconomic element than other common causes of morbidity and death, including cardiac disease. [13] Also, low income appears to have a greater influence on the prevalence of COPD than asthma. [14] In Canada, where the overall prevalence of COPD was 5.69%, this prevalence was found to be significantly higher among older patients, and those who are currently smoking, underweight or obese, and those with total personal income <$ 20,000 and some post-secondary education. [15]

A number of environmental factors can have effects on health, both positive and negative..

Green spaces, parks and community gardens support community health by reducing stress, promoting physical activity, and improving perceived general health. [16] These measures can be identified and modified to promote positive health behaviors and avoid negative ones, [17] and to shape specific promotional and educational initiatives aimed at reducing the prevalence of COPD and its exacerbations.

Recently, *Big Data* analyses have made it possible to measure, analyze, and document the health of individuals and populations, with the aim of improving public health and enhancing decision making at the individual and community levels. [18] Such applications have been used to monitor the quality of medical and healthcare facilities, improve patient service, detect

For peer review only

disease spread earlier, provide new insights into disease mechanisms through risk factors, and improve treatments. [19, 20]

Earlier studies on COPD exacerbations have been mostly prospective and have involved patients from secondary and/or tertiary care. In contrast, the present study examines associations between well-known patient-level risk factors and triggers of exacerbations of COPD, including local community factors.

OBJECTIVES

To estimate the prevalence of COPD in elderly patients in the province of Lodz Poland, and to determine the prevalence of AECOPD in this population.

To identify local community determinants of disease exacerbations among elderly COPD patients.

METHODS

A retrospective observational case-control cross-sectional study was performed among elderly patients with COPD in the Lodz province in Poland. The study was conducted between January 1st and December 31st, 2016.

DATA SOURCES

Data were retrieved from three Big Data databases of the NFZ (National Health Service), US (Tax Office) and GUS (Central Statistical Office).

The NFZ electronic health records (EHRs) database was used to gain information on sociodemographic (age, sex), inpatient and outpatient healthcare utilisation, including costs of hospitalization, and the cost of dispensed medications in the form of ATC (anatomical

For peer review only

therapeutic chemical) codes, as well as other corresponding costs and information on physicians working in primary health care clinics (PHC) in the study area.

The US database provided information on the income of residents aged 65 and older (65+) in the province of Lodz.

The GUS database contained variables potentially associated with exacerbations for 177 gminas (local communities) within the Lodz province.

Local community factors potentially associated with COPD exacerbations in the elderly were selected and classified by experts into (1) health factors, (2) socioeconomic factors and (3) community environmental factors at gmina level, according to the Remington and Catlin methodology [21].

PARTICIPANTS

All participants were patients registered in PHC clinics, aged 65+, residents of Lodz province. All were assigned to a particular gmina; it was assumed that the majority of patients aged 65+ choose a healthcare clinic close to their place of residence. COPD was indicated by the presence of the International Classification of Diseases (ICD-10) code J44 in patients' medical records; exacerbations were defined as cases "hospitalized with the J44 code as a main reason for admission".

Big Data Quality process

The Big Data quality process was carried out according to the SAS Data Management Methodology. After cleaning the data, the rules and alerts for monitoring were defined (details given in APPENDIX).

STATISTICAL ANALYSIS

For peer review only

The data was analysed as described previously in protocol. [22] Additionally, the propensity score matching (PSM) technique and multivariate conditional logistic regression for matched pairs were applied to estimate the local community determinants of AECOPD. [23]

Descriptive statistics were calculated for the total cohort of COPD patients aged 65+ before and after propensity score matching (PSM). Missing data was excluded from the analysis. The relationships between tested variables and exacerbations (1-with, 0-without) were determined using univariate high-performance logistic regression models, and univariate logistic regression models applied and stratified logistic regression (a single model is for each line). Continuous variables were categorized by quartile. Pearson's correlation coefficient was estimated. Patients with exacerbations were matched retrospectively for gender and age to patients without exacerbations to ensure 1:1 group ratio without repetition using propensity score matching (PSM). Patients who died were excluded from the analysis. Costs were calculated as the percentage of costs per patient to the mean COPD patient population and categorized by quartiles (APPENDIX).

After adjusting for potential covariates, the odds ratio (OR) and 95% confidence intervals 95% (CI) of exacerbations between cases and control patients were calculated by multivariate conditional logistic regression for matched pairs models. For the prevalence of the disease, 95% confidence intervals (95%CI) of the fraction of patients in the population were calculated.

Statistically significant findings were assumed as p-value <0.05.

The analysis was performed with the SAS V.9.4 statistical package, MLwiN V.2.24 and STATISTICA V.13.1.

Study size/Power Calculation

The power was calculated for paired t-test for mean difference. Taking number of pairs 1492, calculated mean difference between number of consultations in PHC clinic for patients with

exacerbation and without exacerbations was 1.214 (SD=9.658). The standard deviation for the number of consultations in PHC clinic in group with exacerbation was 7.62 and in group without exacerbation was 6.76 , the correlation between them was 0.103 ($p<0.0001$), which gave the power of 99.8%. A total of 499 pairs would allow 80% power.

Patient and Public Involvement

This was a Big Data study using patients' anonymized electronic health records without patient involvement.

RESULTS

Prevalence

The Lodz province comprised 2 485 323 inhabitants as of December 31, 2016 [24]. According to the NFZ electronic health records (EHRs) database within the Lodz province, 472 314 elderly patients were registered in PHC clinics with contracts with the National Health Fund (19% of total number of residents).

Prevalence of COPD in the population of elderly patients registered in PHC clinics in Lodz province in 2016 was 3.65% (95%CI (3.60-3.70) [%]) (17 240 patients coded as J44) of all patients (n= 472 314). Among all COPD patients, 10.35%, 95%CI (9.89-10.80) [%] (1 784) were recorded as having an exacerbation; this group represents 0.38% of the entire elderly patient population in the Lodz province. During the study, 1 141 of 17 240 (6.62%) patients died and their data was excluded from further analysis. In total, 16 099 COPD patients were included in the analysis.

Characteristics of the COPD patient groups (raw data before PSM)

For peer review

The group characteristic is presented in Supplementary Material Table 1S and APPENDIX Table 1A.

In total, 1 492 (9.27%) of 16 099 COPD patients reported exacerbations. The number of exacerbations per individual COPD patient ranged from one to six. Most of these patients reported one exacerbation (1 294 patients; 86.73%), with fewer reporting two (148 patients; 9.92%), three (32 patients; 2.14%), four (11 patients; 0.74%); five (five patients; 0.34%) and six exacerbations (two patients; 0.13%).

A statistically significant relationship was observed between sex and exacerbations ($p < 0.0001$). 59.38% of patients in group with exacerbation was male. In addition, a significant relationship was noted between age and exacerbations ($p<0.0001$): the COPD group with exacerbations had a higher median age 75 years old (Lower Quartile= 69, Upper Quartile =81) than the COPD patients without exacerbations 73 years old (q1=68, q3=80).

The number of COPD consultations in PHC clinics, classed as the number of health care consultations with the main diagnosis J44, ranged from 0 to 24 per patient during the study year, with the most common value being one consultation in PHC clinic per 65+ COPD patient. Among the people with exacerbation 36.73% did not have any COPD-related consultations in the PHC clinic and 1.14% in the group without exacerbation. 19.17% patients in group with exacerbations had received one COPD consultation (52.72% in group without exacerbation), 12.8% two consultations (20.06% in group without exacerbation), 7.91% three consultations (10.36% in group without exacerbation), 23.39% four or more COPD consultations (15.72% in group without exacerbation).

The number of COPD consultations in 2016 was associated with the number of exacerbations ($p<0.0001$).

The number of consultations for any reason in PHC clinic per 65 +, COPD patient during the study year ranged from 0 to 78, with the most common value being 10 consultations.

For peer review only - http://bmjopen.bmj.com/site/about/guidelines.xhtml

The number of consultations in PHC in 2016 were significantly associated with disease exacerbation (medium vs. low p = 0.0350 and high vs. low p = 0.0092). Among patients with exacerbation, about 46% had 12 or more consultations, 44% had a medium number of consultations (n=5 to 12), and only 10% had low number (n=0 to 4).

In total, 2 465 physicians were working in PHC clinics within the Lodz province, including 627 (25.44%) family doctors, and 1 838 (74.56%) other physicians (general internal medicine, pediatrics, physicians without specialization). Of the total physicians, 1 674 (67.91%) were female. The median age of physicians was 52 (range 25 - 88) years.

Similar percentages of patients whose physician was a family doctor vs. other were observed in the group of COPD patient with exacerbations (39.34%) and in the group without exacerbations (37.54%) (p=0.1703).

There was an association between the numbers of packages of reimbursed drugs and exacerbations (R03 AC, AL, BA, BB, DA: p = 0.0001, AK p = 0.0015, R03DC p= 0.4080), although this variables had n=1906 (11.84% missing data) and need to be carefully interpreted. The COPD group with exacerbations demonstrated higher numbers of packages of reimbursed drugs with ATC codes R03 AC, AL, BA, BB, DA AK except R03DC compared to those without exacerbations, based on prescriptions provided to the patient.

The NFZ drug reimbursement costs per patient were significantly associated with exacerbations (p<0.0001, n=1906 (11.84%) of missing data). There are also a significant associations between total stationary costs and exacerbations (p <0.0001) (APPENDIX). The total NFZ costs per patient (as sum of costs of PHC, ASC, hospitalization, drug reimbursement and other) was significantly associated with exacerbations (p <0.0001) (APPENDIX).

Income was also significantly associated with exacerbations (p <0.0001).

The group with exacerbations demonstrated lower income, by PLN 584.02 per year, compared to the group without exacerbations.

For peer review only

Forest cover of the gmina where the patient lived had a significant impact on the exacerbations (high vs. low p=0.0014, medium vs. low p=0<0.0001). 43% of patients in group with exacerbations were residents of gminas with a lower level of forest cover.

COPD exacerbations were found to be significantly associated with patient's place of residence in gmina (urban, rural, urban-rural).

Patients with COPD exacerbations throughout the one-year study period were matched for gender and age at baseline with patients without COPD exacerbations in a 1:1 ratio: n=1 492 cases (50%) and n=1 492 controls (50%) (before and after PSM: Histograms Fig.1 – Fig.4 in APPENDIX).

The characteristics of COPD patients with and without exacerbations after PSM are given in tables in APPENDIX Table 2A and Supplementary Material Table 2S.

Table 1 shows descriptive characteristics and local determinants univariate analysis of COPD exacerbations in cohort of elderly COPD patients (Lodz province, 2016; case-control). After PSM.

Table 1. Descriptive characteristics of COPD exacerbations in cohort of elderly COPD patients (Lodz province, 2016; case-control). After PSM.

For peer review only

Patient Characteristic (After PSM)	COPD patients without exacerbations Control (n=1492 pairs)	COPD patients with exacerbations Cases (n=1492 pairs)	Total (n=2984)	OR (95% CI) Univariate analysis**
**Patient level**				
**Demographic**				
**COPD Patients (Sum)(%) Total**	1492 (50)	1492 (50)	2984 (100)	
**Sex (strata variable)**				
**Male**	886 (59.38)	886 (59.38)	1772 (59.38)	1 Ref. Cat^.
**Female**	606 (40.62)	606 (40.62)	1212 (40.62)	1.000 (0.864-1.157)^
**Age (years) (strata variable)**				
**<75-85)**	600(40.21)	600(40.21)	1200(40.21)	1.000 (0.788-1.269)^
**<85+>**	175(11.73)	175(11.73)	350(11.73)	1 Ref. cat.^
**<65-75)**	717(48.06)	717(48.06)	1434(48.06)	1.000 (0.792-1.263)^
**Patient's place of residence in gmina (localization)**				
**1-urban vs. 3**	862 (57.77)	864 (57.91)	1726 (57.84)	1.028 (0.848-1.246)
**2-rural vs. 3**	353 (23.66)	358 (23.99)	711 (23.83)	1.04 (0.833-1.3)
**3-urban-rural (Ref. Cat.)**	277 (18.57)	270 (18.1)	547 (18.33)	1 Ref. Cat.
Healthcare use				
**Specialization of physician working in PHC**				
**Family doctors vs. Other**	562(37.67)	587(39.34)	1149(38.51)	1.080(0.926-1.259)
**Other (Ref. Cat.)**	930(62.33)	905(60.66)	1835(61.49)	1 Ref. Cat.
**Number of consultations in PHC^**				
**high (>12) vs. low (<5)**	558 (37.4)	689 (46.18)	1247 (41.79)	1.269 (0.982-1.639)
**medium <5-12> vs. low (<5)**	783 (52.48)	649 (43.5)	1432 (47.99)	0.831 (0.651-1.061)

For peer review only

BMJ Open

**low (<5) (Ref. Cat.)**	151 (10.12)	154 (10.32)	305 (10.22)	1 Ref. Cat.
**Number of COPD consultations in PHC (binary)^^**				
0-1	764 (51.21)	834 (55.90)	1598 (53.55)	p=0.0096^^
2+	728 (48.79)	658 (44.10)	1386 (46.45)	
**Gmina/Commune, post-codes level**				
**Socioeconomic Status**				
**Total personal income of residents per PHC post-codes (Tax Office)**				
**high (>21056,52) vs. low (<17079,50)**	371 (24.87)	345 (23.12)	716 (23.99)	0.696 (0.489-0.990)
**medium <17079,50-21056,52> vs. low (<17079,50)**	759 (50.87)	768 (51.47)	1527 (51.17)	0.910 (0.722-1.147)
**low (<17079,50) (Ref. Cat.)**	362 (24.26)	379 (25.4)	741 (24.83)	1 Ref. Cat.
**Environmental factors**				
**Forest cover of the patient's place of residence in gmina (GUS)**				
**high (>20,60%) vs. low (<7,00%)**	348 (23.32)	340 (22.79)	688 (23.06)	0.896 (0.648-1.24)
**medium <7,00%-20,60%> vs. low (<7,00%)**	776 (52.01)	776 (52.01)	1552 (52.01)	0.94 (0.688-1.285)
**low (<7,00%) (ref. Cat.)**	368 (24.66)	376 (25.2)	744 (24.93)	1 Ref. Cat.
**Total**	1492 (50)	1492 (50)	2984 (100)	

CI=Confidence Interval, OR=Odds Ratio. #=Odds Ratio Estimates (Point Estimate). 95% Wald Confidence Limits. OR (95% CI).
Ref. cat.=reference category. Df=degree of freedom.
^- Sex and age were strata variables (the LOGISTIC Procedure, Logistic Regression).
^^- McNemar's test B/C (□^{2}=6.71, df=1, p=0.0096) in STATISTICA 13.
**The LOGISTIC Procedure, Conditional Logistic Regression for Matched Pairs.

For peer review only

PSM analysis identified similar relationships between variables and exacerbations as in the raw data. Median age was the same for patients with and without exacerbations (75 years, q1=69, q3=81, for both groups).

Patient consultations, and consultations for COPD in PHC clinics had a weak association with exacerbations (high vs low p = 0.0687).

High number of consultations in PHC clinics (any reason) had 46.18% of patients in group with exacerbations and 37.40% in group without exacerbations.

In addition, 36.73% patients with exacerbations and 0.87% of patients without exacerbations had not received any COPD consultations in PHC clinic. When the number of consultations in PHC with the main diagnosis J44 increased from one to three, a decrease in the number of exacerbations was observed.

There was an association between the numbers of packages of reimbursed drugs and exacerbations (R03 AC, AL, BA, BB, DA: p = 0.0001, AK p = 0. 0.0078, R03DC p= 0. 0.9022. Patients with exacerbations used a greater number of prescriptions for reimbursed drug packages. Patients with exacerbations had higher numbers of prescriptions of reimbursed drugs from ATC groups with codes R03AC, AL, BA, BB, DA, AK compared to those without exacerbations. Again the numbers of R03DC packages did not differ significantly between the groups.

Significant associations was found between total NFZ cost per patient (as the sum of costs of PHC, ASC, hospitalization, drugs reimbursement and other) and COPD exacerbations for both data before PSM (p <0.0001) (APPENDIX table 3A) and after PSM (p = <0.0001) (APPENDIX table 3B).

For peer review only

Of all patients in the exacerbations group, 37.53% had high total NFZ cost per patient, and 58.65% had medium. Among those without exacerbations, the costs were high in only 12%, medium in 41%, and 46% had low costs.

A significant association was found between income and disease exacerbations (high vs. low p= 0.0439). There were fewer exacerbations in the group of patients with high income.

There was no association between the forest cover of the patient's place of residence in gmina and exacerbations (high vs. low p=0.5082, medium vs. low p=0.6986).

Model of local determinants of COPD exacerbations for elderly COPD patients

Table 2 shows local determinants of COPD exacerbations for 65+ patients. For more descriptive characteristics of the determinants please see Supplementary Material and APPENDIX.

Table 2. Local determinants of COPD exacerbations in cohort of elderly COPD patients (Lodz province, 2016; case-control). After PSM.

	**Final model Conditional Logistic Regression for Matched Pairs**
**Local determinants of COPD exacerbations (After PSM)**	**OR (95% Wald Confidence Limits), (point estimate).**
**Patient level**	
**Demographic**	
**COPD Patients (Sum)(%) Total**	N=1492 pairs
**Patient's place of residence in gmina (localization)**	
**1-urban vs. 3**	1.044 (0.673-1.620)
**2-rural vs. 3**	0.897 (0.630-1.277)
**3-urban-rural (Ref. Cat.)**	1 Ref. Cat.
**Healthcare uses**	
**Specialization of physician working in PHC**	

For peer review only

**Family doctors vs. Other**	1.076 (0.920-1.257)
**Other (Ref. Cat.)**	1 Ref. Cat.
**Number of consultations in PHC^**	
**high (>12) vs. low (<5)**	1.261 (0.974-1.633)
**medium <5-12> vs. low (<5)**	0.823 (0.643-1.053)
**low (<5) (Ref. Cat.)**	1 Ref. Cat.
**Gmina/Commune, post-codes level**	
**Socioeconomic Status**	
**Total personal income of residents per PHC post-codes (Tax Office)**	
**high (>21056,52) vs. low (<17079,50)**	0.601 (0.385-0.939)
**medium <17079,50-21056,52> vs. low (<17079,50)**	0.824 (0.581-1.169)
**low (<17079,50) (Ref. Cat.)**	1 Ref. Cat.
**Environmental factors**	
**Forest cover of the patient's place of residence in gmina (GUS)**	
**high (>20,60%) vs. low (<7,00%)**	0.897 (0.605-1.331)
**medium <7,00%-20,60%> vs. low (<7,00%)**	0.925 (0.648-1.322)
**low (<7,00%) (ref. Cat.)**	1 Ref. Cat.

OR=Odds Ratio Estimates (Point Estimate). 95% Wald Confidence Limits. Ref. cat.=reference category.
#The LOGISTIC Procedure, Model binary logit, 1:1 Matched Case-Control Conditional Logistic Regression for Matched Pairs. Sex and age variables were included as stratification variables (the 1:1 matched case-control), thus they are not explicitly included in the Conditional Logistic Regression for Matched Pairs models.

For peer review only

The final model includes five independent variables: number of consultations in PHC, income, physicians' specialization in PHC, the area covered with forest in gmina of patient's residence and location of gmina (urban, rural, urban-rural).

Two variables seemed important: number of consultations in PHC (border significance) and income.

Patients with a medium number of consultations in PHC demonstrated a slightly lowerchance of exacerbations than those with a low number of consultations (medium <5-12> vs low (<5) OR=0.823, 95% CI (0.643-1.053)) (table 2).

COPD patients with a high number of consultations (>12) in PHC showed a trend for more exacerbations compared to those with a low number (<5) of consultations (OR 1.261, CI 0.974-1.633).COPD patients living in areas with a high income had a lower chance of exacerbations than those in areas with a low income (high vs low OR = 0.601 (0.385-0.939)). Living in a high income area is a positive and protective factor vs. living in a low income one.

Type of physician's specialization in PHC had no influence on the exacerbations (family doctor vs other OR = 1.076, 95% CI (0.920-1.257) neither the forest cover area (high vs low OR=0.897, 95% CI (0.605-1.331), medium vs low OR=0.925, 95% CI (0.648-1.322)) or patient's place of residence in gmina (urban vs. urban-rural OR=0.897, 95% CI (0.630-1.277), rural vs. urban-rural).

DISCUSSION

Despite significant advances in the diagnosis and treatment of COPD, its incidence remains high. In addition, in some cases, the chance of exacerbation may increase as a result of local risk factors.

For peer review only - http://bmjopen.bmj.com/site/about/guidelines.xhtml

Fortunately, nowadays information on community health and health care system can be easily gathered from a number of large databases, as Big Data, and analyzed statistically. [25]

Big Data technology can be used to improve the course of chronic disease and its exacerbations. For example, Korean researchers were able to match COPD exacerbations with external factors. [26, 27]

Similarly, the present study applied Big Data analysis to a large primary care cohort of 472 314 patients aged 65 years and older in the Lodz province, Poland. The results yielded new information on disease prevalence and its relationship with local community factors (demographic factors, healthcare use, economic and environmental factors).

A meta-analysis of COPD prevalence reported a pooled prevalence of 9.8% among men and 5.6% among women. [28]

In present study, this value was 3.65% among all elderly patients. COPD exacerbations, hospitalizations and deaths in women have been increasing in several countries, with more women being hospitalized for COPD in recent years [29]; indeed, the percentage of hospitalizations among women in Puglia, Italy was found to increase from 27.8% in 2001 to 35.6% in 2011. [30]

However, in our study 59.38% of COPD patients with exacerbation were male (raw data $p<0.0001$).

More research is needed to verify these apparent gender differences, not only among hospitalized patients but also in their daily environment.

In a Danish study, the risk of hospitalization for COPD increased with age until ~80 years for females and 85 years for males. [31]

Our findings also indicate a significant relationship between age and exacerbation (raw data $p<0.0001$). Of those with exacerbations, 47% are aged 65-75 years, 40% are aged 75-85 years, and approximately 14% are 85 years of age or older. In our study, the age of patients in the

For peer review only

exacerbation group was higher than in group without exacerbation: 75 years old vs. 74 (raw data p<0.0001)

The high number of primary care consultations per patient and a low income in the local area were important determinants of exacerbations among COPD patients aged 65+ (p=0.0784 border of significance and p=0.0253, respectively), whereas there was no effect of specialization of the PHC physician, forest cover of the local area, and the location of the gmina on rates of exacerbations.

In the present study, patients with a higher number of consultations had a slightly higher risk of exacerbations compared to patients with a low number of consultations (OR= 1.261; 95% CI=0.974, 1.633).

It is likely that the former group have a greater chance of referral to a hospital by a primary care physician, and thus faster diagnosis and treatment.

Patients with high income are less likely to have COPD exacerbations than those with low income (OR= 0.601; 95% CI 0.385, 0.939). Other studies have reported similar trends. [32] This may be related to decreased access to health care services among lower income groups. Such problems with access may delay the diagnosis of COPD, leading to a poorer prognosis and increased morbidity and mortality. [33] Addressing this issue may reduce the workload of the healthcare system and allow more targeted use of available resources. Low income in the local area was important determinant of exacerbations among COPD patients aged 65+ and it can be explained by a number of other co-factors such as: more severe COPD, co-morbidities, less inclination to use regular inhalers / medications, less healthy lifestyle or poor living conditions. The association between neighbourhood greenness and respiratory outcomes in adults appears ambiguous. [34] One cohort study including 1.3 million adults in Canada found that greater neighbourhood greenness could reduce the risk of respiratory mortality [35];

however, a Chinese cohort study concluded that neighborhood greenness might in fact be a risk factor. [36]

In our present study, forest cover per gmina was not found to have any statistically significant effect on COPD exacerbations

Rural residence has been found to be associated with an increased incidence of COPD exacerbations, although not severe ones. [37] In countries with large rural populations, COPD is more prevalent in rural than urban areas. This disparity may be related partly to increased agricultural dust/aerosol exposure (wheat, grain, cotton dust, pesticides). [38] In addition, studies suggest an association with increased indoor pollution due to burning biomass fuels. [39]

In our study, patient's place of residence in gmina (urban, rural, urban-rural) did not appear to have any influence on the COPD exacerbations.

In addition, the specialization of the physician in primary care did not have any effect on the COPD exacerbations.

Identification of patient population relied only on the ICD-10 J-44 code in patients primary care records, so COPD patient in specialist clinics and on hospitals wards without this ICD-10 code might not be identified, what can be perceived as study limitation. The validation of the J-44 Code of the patients enrolled in the study was not possible at the PHC clinics level because the NFZ provided us with anonymised patient data.

Patients with code J44 were included in this study, without excluding codes for other diseases, including asthma, due to missing data in the variable "Codes for comorbidities with COPD" (98.83% (466 780 out of 472 314) in total 65+ patients in PHC in Lodz province in 2016), so the variable was excluded from the analysis.

Another limitation of this study was lack of standardization in the method of recording exacerbations between primary care, accident and emergency departments and hospital wards.

CONCLUSIONS

This study investigates the determinants for AECOPD with the aim of developing activities to improve prevention and treatment interventions. Our findings regarding COPD in the Lodz province, Poland may also be used for formulating health policy recommendations by the National Health Fund (NFZ) and local government. The Big Data methodological approach used herein can be applied to other chronic diseases. Further study is needed to determine the role of local medical and environmental factors in improving the health of COPD patients.

ACKNOWLEDGMENTS

We acknowledge the role of experts from various fields: primary care physicians, public health specialists, a Big Data analyst and data managers in public institutions (NFZ, GUS and US) in the study.

The authors specially thank Edward Lowczowski for English language assistance.

Funding Statement

This work was supported by Narodowe Centrum Nauki (National Science Centre, Poland) grant number 2016/21/B/NZ7/02052.

Competing interests statement.

No other relationships or activities that could appear to have influenced the submitted work.

Data sharing

No additional data available.

For peer review only

Research Ethics Approval

The study protocol has been approved by the Bioethical Committee of Medical University of Lodz (RNN/248/18/KE, 10 July 2018).

This Big Data study using patients' anonymized electronic health records data.

Author’s contribution

The study design was conceptualized by A.K., M.G.-C., K.K. and I.Z. Analyses were performed by I.Z., K.K. and I.Z. prepared the first draft of the manuscript. All authors have read and agreed to the published version of the manuscript.

For peer review only

References

1. WHO. Newsroom. The top 10 causes of death. WHO's Global Health Estimates 2021 [05.11.2021]: Available from: https://www.who.int/news-room/fact-sheets/detail/the-top-10-causes-of-death
2. Adeloye D, Chua S, Lee C, Basquill C, Papana A, Theodoratou E, et al. Global and regional estimates of COPD prevalence: Systematic review and meta-analysis. *J. Glob. Health* 2015; 5:020415.
3. Boven Jv, Gaughan J, Soriano J, Correia de Sousa J, Baxter N, Román-Rodríguez M, et al. P139 The burden of copd across the european union: development of the european copd atlas. *Thorax* 2016;71(Suppl 3):A158-A.
4. Gibson GJ, Loddenkemper R, Lundback B, Sibille Y. Respiratory health and disease in Europe: the new European Lung White Book. *Eur Respir J* 2013; 42:559-63.
5. Blanco I, Diego I, Bueno P, Fernandez E, Casas-Maldonado F, Esquinas C, et al. Geographical distribution of COPD prevalence in Europe, estimated by an inverse distance weighting interpolation technique. *Int J Chron Obstruct Pulmon Dis* 2018;13:57-67.
6. Mullerova H, Shukla A, Hawkins A, Quint J. Risk factors for acute exacerbations of COPD in a primary care population: a retrospective observational cohort study. *BMJ Open* 2014;18:e006171.
7. Perera PN, Armstrong EP, Sherrill DL, Skrepnek GH. Acute exacerbations of COPD in the United States: inpatient burden and predictors of costs and mortality. *COPD* 2012;9:131-41.
8. Qureshi H, Sharafkhaneh A, Hanania NA. Chronic obstructive pulmonary disease exacerbations: latest evidence and clinical implications. *Ther Adv Chronic Dis* 2014;5:212-27.
9. Esteban C, Quintana JM, Moraza J, Aburto M, Egurrola M, Espana PP, et al. Impact of hospitalisations for exacerbations of COPD on health-related quality of life. *Respir Med* 2009;103:1201-8.
10. Hurst JR, Vestbo J, Anzueto A, Locantore N, Mullerova H, Tal-Singer R, et al. Susceptibility to exacerbation in chronic obstructive pulmonary disease. *N Engl J Med* 2010;363:1128-38.
11. Husebo GR, Bakke PS, Aanerud M, Hardie JA, Ueland T, Gronseth R, et al. Predictors of exacerbations in chronic obstructive pulmonary disease--results from the Bergen COPD cohort study. *PLoS One* 2014;9:e109721.
12. Kerkhof M, Freeman D, Jones R, Chisholm A, Price DB, Grp RE. Predicting frequent COPD exacerbations using primary care data. *Int J Chron Obstruct Pulmon Dis* 2015;10:2439-50.
13. Steenland K, Hu S, Walker J. All-cause and cause-specific mortality by socioeconomic status among employed persons in 27 US states, 1984-4997. *Am. J. Public Health* 2004;94:1037-42.
14. Pleasants RA, Ohar JA, Croft JB, Liu Y, Kraft M, Mannino DM, et al. Chronic Obstructive Pulmonary Disease and Asthma - Patient Characteristics and Health Impairment. *COPD* 2014;11:256-66.
15. Osman S, Ziegler C, Gibson R, Mahmood R, Moraros J. The Association between Risk Factors and Chronic Obstructive Pulmonary Disease in Canada: A Cross-sectional Study Using the 2014 Canadian Community Health Survey. *Int J Prev Med*2017;8:86.
16. Maas J, Verheij RA, Groenewegen PP, de Vries S, Spreeuwenberg P. Green space, urbanity, and health: how strong is the relation? *J Epidemiol Community Health* 2006;60:587-92.
17. Jakubowski B, Frumkin H. Environmental Metrics for Community Health Improvement. *Prev Chronic Dis* 2010;7:A76.

For peer review only

18. Galea S, Abdalla SM, Sturchio JL. Social determinants of health, data science, and decision-making: Forging a transdisciplinary synthesis. *PLoS Med* 2020;17:e1003174.
19. Dinov ID, Heavner B, Tang M, Glusman G, Chard K, Darcy M, et al. Predictive Big Data Analytics: A Study of Parkinson's Disease Using Large, Complex, Heterogeneous, Incongruent, Multi-Source and Incomplete Observations. *PLoS One* 2016;11:e0157077.
20. Tan SS, Gao G, Koch S. Big Data and Analytics in Healthcare. *Methods Inf Med* 2015;54:546-7.
21. Remington PL, Catlin BB, Gennuso KP. The County Health Rankings: rationale and methods. *Popul Health Metr* 2015;13:11.
22. Zakowska I, Kosiek K, Kowalczyk A, Grabowski J, Godycki-Cwirko M. Community determinants of COPD exacerbations in elderly patients in Poland: protocol for a retrospective Big Data observational cohort study. *BMJ Open* 2019;9:e030524.
23. Morgan AD, Herrett E, De Stavola BL, Smeeth L, Quint JK. COPD disease severity and the risk of venous thromboembolic events: a matched case-control study. *Int J Chron Obstruct Pulmon Dis* 2016;**11**:899-908.
24. GUS. Łódzkie Voivodship 2017 - Subregions, Powiats, Gminas. Statistical Office in Łódź. Statistical Yearbook of Łódzkie Voivodship 2017. GUS Publishing 2017 https://lodz.stat.gov.pl/en/publications/statistical-yearbook/lodzkie-voivodship-2017-subregions-powiats-gminas,2,19.html
25. Wamba SF, Akter S, Edwards A, Chopin G, Gnanzou D. How 'big data' can make big impact: Findings from a systematic review and a longitudinal case study. *Int J Prod Econ* 2015;165:234-46.
26. Lee J, Jung HM, Kim SK, Yoo KH, Jung KS, Lee SH, et al. Factors associated with chronic obstructive pulmonary disease exacerbation, based on big data analysis. *Sci. Rep* 2019;9:6679.
27. Rhee CK, Kim JW, Yoo KH, Jung KS. Prediction model of COPD acute exacerbation with big data by machine learning methods. Virtual Congress. 2020;4911 https://www.ers-education.org/lr/show-details/?idP=247196
28. Halbert RJ, Natoli JL, Gano A, Badamgarav E, Buist AS, Mannino DM. Global burden of COPD: systematic review and meta-analysis. *Eur Respir J* 2006;28:523-32.
29. Han MK, Postma D, Mannino DM, Giardino ND, Buist S, Curtis JL, et al. Gender and chronic obstructive pulmonary disease: why it matters. *Am J Respir Crit Care Med* 2007;176:1179-84.
30. Moretti AM, Gallone MS, Parisi D, Tafuri S, Germinario C. Gender differences and hospitalization for COPD: an analysis of data from Puglia (Italy). *Ital J Gender-Specific Med* 2015;1:66-72.
31. Lykkegaard J, dePont Christensen R, Davidsen JR, Stovring H, Andersen M, Sondergaard J. Trends in the lifetime risk of COPD exacerbation requiring hospitalisation. *Eur Respir J* 2013;42:964-71.
32. Eisner MD, Blanc PD, Omachi TA, Yelin EH, Sidney S, Katz PP, et al. Socioeconomic status, race and COPD health outcomes. *J Epidemiol Community Health* 2011;65:26-34.
33. van Doorslaer E, Masseria C, Koolman X. Inequalities in access to medical care by income in developed countries. *CMAJ* 200617;174:177-83.
34. Fong KC, Hart JE, James P. A Review of Epidemiologic Studies on Greenness and Health: Updated Literature Through 2017. *Curr Env Hlth Rep* 2018;5:77-87.
35. Crouse DL, Pinault L, Balram A, Hystad P, Peters PA, Chen H, et al. Urban greenness and mortality in Canada's largest cities: a national cohort study. *Lancet Planet Health* 2017;1:e289-e97.

For peer review only - http://bmjopen.bmj.com/site/about/guidelines.xhtml

36. Fan J, Guo Y, Cao Z, Cong S, Wang N, Lin H, et al. Neighborhood greenness associated with chronic obstructive pulmonary disease: A nationwide cross-sectional study in China. *Environ Int* 2020;144:106042.
37. Burkes RM, Gassett AJ, Ceppe AS, Anderson W, O'Neal WK, Woodruff PG, et al. Rural Residence and Chronic Obstructive Pulmonary Disease Exacerbations. Analysis of the SPIROMICS Cohort. *Ann Am Thorac Soc* 2018;15:808-16.
38. Hu G, Zhou Y, Tian J, Yao W, Li J, Li B, et al. Risk of COPD from exposure to biomass smoke: a metaanalysis. *Chest* 2010;138:20-31.
39. Perez-Padilla R, Regalado J, Vedal S, Pare P, Chapela R, Sansores R, et al. Exposure to biomass smoke and chronic airway disease in Mexican women. A case-control study. *Am J Respir Crit Care Med* 1996;154:701-6.

For peer review only

Supplementary: Table 1S Descriptive characteristics and univariate analysis of COPD exacerbations in cohort of COPD elderly patients and prevalence of COPD in PHC (Lodz province in 2016). (Raw data before PSM).

**Patient Characteristic (Before PSM)**	**COPD patients without exacerbations (n=14607)**	**COPD patients with exacerbations (n=1492)**	**Total (n=16099)**	**OR (95% CI), p-value #Univariate Logistic Regression.**
Patient level				
Demographic				
COPD Patients (Sum)(%) Total	14607 (90.73)	1492 (9.27)	16099 (100)	
Sex				
Male	7652(52.39)	886(59.38)	8538(53.03)	1.329(1.192-1.481)
Female	6955(47.61)	606(40.62)	7561(46.97)	1 Ref. Cat.
Age (years)				
<75-85)	5046(34.55)	600(40.21)	5646(35.07)	1.322(1.18-1.482)
<85+>	1587(10.86)	175(11.73)	1762(10.94)	1.226(1.031-1.459)
<65-75)	7974(54.59)	717(48.06)	8691(53.98)	1 Ref. Cat.
Patient’s place of residence in gmina (localization)				
1-urban	8971(61.42)	864(57.91)	9835(61.09)	0.840(0.728-0.971)
2-rural	3280(22.45)	358(23.99)	3638(22.6)	0.952(0.806-1.125)
3-urban-rural	2356(16.13)	270(18.1)	2626(16.31)	1 Ref. Cat.
Healthcare uses				
Specialization of physician working in PHC				
Family doctors	5483(37.54)	587(39.34)	6070(37.7)	1 Ref. Cat.
Other	9124(62.46)	905(60.66)	10029(62.3)	0.926(0.831-1.033)
Number of consultations in PHC^				
high (>12)	5309(36.35)	689(46.18)	5998(37.26)	1.277(1.063-1.535)
medium <5-12>	7783(53.28)	649(43.5)	8432(52.38)	0.820(0.682-0.986)

For peer review only

low (<5)	1515(10.37)	154(10.32)	1669(10.37)	1 Ref. Cat.
Number of COPD consultations in PHC^				
0	166 (1.14)	548 (36.73)	714 (4.44)	21.718 (17.658-26.712)
1	7701 (52.72)	286 (19.17)	7987 (49.61)	0.244 (0.208-0.288)
2	2930 (20.06)	191 (12.8)	3121 (19.39)	0.429 (0.357-0.516)
3	1514 (10.36)	118 (7.91)	1632 (10.14)	0.513 (0.412-0.638)
4+	2296 (15.72)	349 (23.39)	2645 (16.45)	1 Ref. Cat.
Gmina/Commune, post-codes level				
Socioeconomic Status				
Total personal income of residents per PHC post-codes (Tax Office)				
high (>21992,62)	3717 (25.45)	240 (16.09)	3957 (24.58)	0.573 (0.486-0.677)
medium <17612,68-21992,62>	7231 (49.5)	840 (56.3)	8071 (50.13)	1.032 (0.911-1.168)
low (<17612,68)	3659 (25.05)	412 (27.61)	4071 (25.29)	1 Ref. Cat.
Environmental factors				
Forest cover of the patient's place of residence in gmina (GUS)				
high (>17,10%)	3043 (20.83)	308 (20.64)	3351 (20.81)	0.791 (0.686-0.913)
medium <8,40%-17,10%>	6583 (45.07)	547 (36.66)	7130 (44.29)	0.65 (0.576-0.733)
low (<8,40%)	4981 (34.1)	637 (42.69)	5618 (34.9)	1 Ref. Cat.
Total	14607 (90.73)	1492 (9.27)	(100)	

^Pearson Correlation Coefficient=0,2468, p<0.0001 between "Number of consultations in PHC" and "Number of COPD consultations in PHC". CI=Confidence Interval, OR=Odds Ratio. #=Odds Ratio Estimates (Point Estimate). 95% Wald Confidence Limits. OR (95% CI). #Univariate analysis Univariate Logistic Regression. ##Univariate High-Performance Logistic Regression

For peer review only

Table 2S. Descriptive characteristics and local determinants of COPD exacerbations in cohort of COPD elderly patients (Lodz province, 2016; case-control). After PSM.

**Patient Characteristic (After PSM)**	**COPD patients without exacerbations Control (n=1492 pairs)**	**COPD patients with exacerbations Cases (n=1492 pairs)**	**Total (n=2984)**	**OR (95% CI) Univariate analysis****	**OR (95% CI)**
**Patient level**					
**Demographic**					
**COPD Patients (Sum)(%) Total**	1492 (50)	1492 (50)	2984 (100)		
**Sex (strata variable)**					
**Male**	886 (59.38)	886 (59.38)	1772 (59.38)	1 Ref. Cat^	Strata variable
**Female**	606 (40.62)	606 (40.62)	1212 (40.62)	1.000 (0.864-1.157)^	
**Age (years) (strata variable)**					
**<75-85)**	600(40.21)	600(40.21)	1200(40.21)	1.000 (0.788-1.269)^	Strata variable
**<85+>**	175(11.73)	175(11.73)	350(11.73)	1 Ref. cat.^	
**<65-75)**	717(48.06)	717(48.06)	1434(48.06)	1.000 (0.792-1.263)^	
**Patient's place of residence in gmina (localization)**					
**1-urban vs. 3**	862 (57.77)	864 (57.91)	1726 (57.84)	1.028 (0.848-1.246)	1.044 (0.673-1.620)
**2-rural vs. 3**	353 (23.66)	358 (23.99)	711 (23.83)	1.04 (0.833-1.3)	0.897 (0.630-1.277)
**3-urban-rural (Ref. Cat.)**	277 (18.57)	270 (18.1)	547 (18.33)	1 Ref. Cat.	1 Ref. Cat.
**Healthcare uses**					
**Specialization of physician working in PHC**					
**Family doctors vs. Other**	562(37.67)	587(39.34)	1149(38.51)	1.080(0.926-1.259)	1.076 (0.920-1.257)
**Other (Ref. Cat.)**	930(62.33)	905(60.66)	1835(61.49)	1 Ref. Cat.	1 Ref. Cat.
**Number of consultations in PHC^**					
**high (>12) vs. low (<5)**	558 (37.4)	689 (46.18)	1247 (41.79)	1.269 (0.982-1.639)	1.261 (0.974-1.633)

For peer review only

**medium <5-12> vs. low (<5)**	783 (52.48)	649 (43.5)	1432 (47.99)	0.831 (0.651-1.061)	0.823 (0.643-1.053)
**low (<5) (Ref. Cat.)**	151 (10.12)	154 (10.32)	305 (10.22)	1 Ref. Cat.	1 Ref. Cat.
**Number of COPD consultations in PHC^**					
**0 vs. 4+**	13 (0.87)	548 (36.73)	561 (18.8)	41.078 (18.064-93.413)	
**1 vs. 4+**	751 (50.34)	286 (19.17)	1037 (34.75)	0.288 (0.224-0.37)	
**2 vs. 4+**	299 (20.04)	191 (12.8)	490 (16.42)	0.488 (0.369-0.645)	
**3 vs. 4+**	185 (12.4)	118 (7.91)	303 (10.15)	0.528 (0.377-0.738)	
**4+ (Ref. Cat.)**	244 (16.35)	349 (23.39)	593 (19.87)	Ref. Cat.	
**Gmina/Commune, post-codes level**					
**Socioeconomic Status**					
**Total personal income of residents per PHC post-codes (Tax Office)**					
**high (>21056,52) vs. low (<17079,50)**	371 (24.87)	345 (23.12)	716 (23.99)	0.696 (0.489-0.990)	0.601 (0.385-0.939)
**medium <17079,50-21056,52> vs. low (<17079,50)**	759 (50.87)	768 (51.47)	1527 (51.17)	0.910 (0.722-1.147)	0.824 (0.581-1.169)
**low (<17079,50) (Ref. Cat.)**	362 (24.26)	379 (25.4)	741 (24.83)	1 Ref. Cat.	1 Ref. Cat.
**Environmental factors**					
**Forest cover of the patient's place of residence in gmina (GUS)**					
**high (>20,60%) vs. low (<7,00%)**	348 (23.32)	340 (22.79)	688 (23.06)	0.896 (0.648-1.24)	0.897 (0.605-1.331)
**medium <7,00%-20,60%> vs. low (<7,00%)**	776 (52.01)	776 (52.01)	1552 (52.01)	0.94 (0.688-1.285)	0.925 (0.648-1.322)
**low (<7,00%) (ref. Cat.)**	368 (24.66)	376 (25.2)	744 (24.93)	1 Ref. Cat.	1 Ref. Cat.
**Total**	1492 (50)	1492 (50)	2984 (100)		

CI=Confidence Interval, OR=Odds Ratio. #=Odds Ratio Estimates (Point Estimate). 95% Wald Confidence Limits. OR (95% CI).
Ref. cat.=reference category.
^- Sex and age were strata variables (the LOGISTIC Procedure, Logistic Regression).
**The LOGISTIC Procedure, Conditional Logistic Regression for Matched Pairs.

For peer review only

Descriptive characteristics of determinants

"Patient with ICD-10 code J44." - Determination whether J44 ICD-10 code is present in patients' medical records in PHC clinics

"Exacerbations" - "Hospitalization because of J44." - Determination of whether the patient was "hospitalized with the J44 code as a main reason for admission. (Main diagnosis checked at the hospitalization level and not at individual wards).

"Patient's place of residence in gmina" (localization) - based on patients' PHC clinic address with assigned post- code

Division into the type of communes according to the Central Statistical Office: 1-urban, 2-rural, 3-urban-rural (Ref. Cat.). GUS Kind of gminas name: https://bdl.stat.gov.pl/BDL/metadane/teryt/rodzaj

"Specialization of physician working in PHC": Family doctors, Other (Ref. Cat.) - Specialization of the physician working in PHC clinic within the Lodz province - family doctors, and other physicians (internal medicine specialist, pediatrician, physician without specialization). (NFZ database)

"Number of consultations in PHC": high (>12), medium <5-12>, low (<5) (Ref. Cat.) - The number of patient's consultations for any reason in PHC clinic in 2016

"Number of COPD consultations in PHC": 0, 1, 2, 3, 4+ (Ref. Cat.) – the number of patient's health care consultations with the main diagnosis J44in 2016

"Codes of diseases associated with COPD (PHC)" – codes of comorbidities in the medical record of a patient with COPD. in 2016

"Total personal income of residents per PHC post-codes" – (Tax Office): high (>21056,52 PLN), medium <17079,50 PLN -21056,52 PLN > , low (<17079,50 PLN) (Ref. Cat.)

"Forest cover of the patient's place of residence in gmina" (GUS): high (>20,60%), medium <7,00%-20,60%>, low (<7,00%) (ref. Cat.) in 2016

"The total cost of STAC" - The NFZ costs for all hospital services in 2016 for a given patient, regardless of the main diagnosis

For peer review only

"Total cost of drug reimbursement" - The NFZ costs for all prescribed medications in 2016 for a given patient

"Total NFZ cost" - The NFZ cost – (as sum of costs of PHC, ASC, hospitalization, drug reimbursement and other) in 2016 per patient, regardless of the main diagnosis

For peer review only

APPENDIX

Table 1A. Descriptive characteristics of cohort of COPD elderly patients within Lodz province (Raw data before PSM, n=16 099) and prevalence of COPD, including prevalence of COPD exacerbations (n=17 240, including deaths).

Characteristic	COPD without exacerbations (n=14 607)	COPD with exacerbations (n=1 492)	Total (n=16 099)	Odds Ratio Estimates Point Estimate 95% Wald Confidence Limits OR [95% CI] - Univariate analysis# HP Logistic Regression
**Total J44 Patients Sum (%)**	14 607 (90.73)	1 492 (9.27)	16 099	
**Exacerbation number**	0	1	Total	Data only for the group with exacerbations.
**1**	0 (0)	1 294 (86.73)	1 294 (8.04)	
**2**	0 (0)	148 (9.92)	148 (0.92)	
**3**	0 (0)	32 (2.14)	32 (0.2)	
**4**	0 (0)	11 (0.74)	11 (0.07)	
**5**	0 (0)	5 (0.34)	5 (0.03)	
**6**	0 (0)	2 (0.13)	2 (0.01)	
**Exacerbation number**				
**(min. - max.)**	0	(1 - 6)	(1 - 6)	
**Missing**	14 607	0	14 607	Data only for the group with exacerbations.
**Total (including Missing)**	14 607 (90.73)	1 492 (9.27)	16 099 (100)	
**Age of the patient 65+Median(q1 - q3)**	73 (68 - 80)	75 (69 - 81)	74 (69 - 80)	1.016 [1.009, 1.023]

For peer review only - http://bmjopen.bmj.com/site/about/guidelines.xhtml

				**Continuous var.
**Number of COPD consultations (PHC)**				
**(min. - max.)**	(0 - 24)	(0 - 20)	(0 - 24)	
**Number of consultations in PHC (min. - max.) **Continuous var.**	(0 - 78)	(0 - 59)	(0 - 78)	
**Drugs## Number of packages Avg±SD**				
**R03AC***	5±5.7	6±6.95	5±5.86	1.036 [1.028,1.044] **Continuous var.
**R03AK***	2±3.83	2±3.99	2±3.84	1.022 [1.008,1.035] **Continuous var.
**R03AL***	1±(5.39	3±8.99	1±5.89	1.041 [1.034, 1.048] **Continuous var.
**R03BA***	2±4.51	4±5.72	2±4.66	1.043 [1.034, 1.053] **Continuous var.
**R03BB***	5±6.11	6±7.3	5±6.27	1.037 [1.029, 1.044] **Continuous var.
**R03DA***	3±4.99	4±5.12	3±5.02	1.038 [1.028, 1.048] **Continuous var.
**R03DC***	0±1.68	0±1.52	0±1.67	0.985 [0.952, 1.020] **Continuous var.
**COST OF MEDICINES (Drugs) [%]**	Percent of median cost per COPD with exacerbations/median cost per COPD without exacerbations [%]			

For peer review only - http://bmjopen.bmj.com/site/about/guidelines.xhtml

**Total Fund Cost (Drugs) in [%]NMiss=1906 (11.84%)**	144.69%			HP LR estimate= 0.000316 **Continuous var.
**Income per post code NMiss=99 Difference between median COPD with exacerbations and median without exacerbations [PLN]**	584.02 PLN			**Continuous
**Did the patient die in 2016?**				
**No**	14 607 (94.51)	1 492 (83.63)	16 099 (93.38)	
**Yes**	849 (5.49)	292 (16.37)	1 141 (6.62)	
**Total**	15 456 (89.65)	1 784 (10.35)	17 240 (100)	
**Physicians'characteristics in PHC in Lodz Voivodeship**				
**Physicians'Age**				
**Median(q1-q3)**	52 (43.61)			
**Number**	2 465			
**Physician's sex**				
**Women=0**	1 674 (67.91)			
**Men=1**	791 (32.09)			
**Total**	2 465 (100)			
**Physician's spacialization**				
**Other=0**	1 838 (74.56)			
**Family doctor=1**	627 (25.44)			
**Total**	2 465 (100)			
**Total Physicians in Lodz voivodeship****	2 465 (100)			
**Prevalence**				
**Prevalence of total of COPD n=17240 among 65+ n=472 314 in Lodz Voivodeship, in PHC in 2016 (including deaths) = 3.65%, 95%CI (3.60-3.70) [%].**				
**Prevalence of total of exacerbations n=1784 among COPD 65+ n=17240 in Lodz Voivodeship, in PHC in 2016 (including death)= 10.35%, 95%CI (9.89-10.80) [%].**				

For peer review only - http://bmjopen.bmj.com/site/about/guidelines.xhtml

Patient Characteristic	COPD without exacerbations (n=15 456)	COPD with exacerbations (n=1 784)	Total (n=17 240)	OR (95% CI) - Univariate analysis Logistic Regression
**Total Patients COPD 65+ including deaths**	15 456 (89.65)	1 784 (10.35)	17 240 (100)	
**Sex**				
**Female**	7 256 (46.95)	708 (39.69)	7 964 (46.19)	F Ref. Cat.
**Male**	8 200 (53.05)	1 076 (60.31)	9 276 (53.81)	1.345 [1.217,1.486]
**Total**	15 456 (89.65)	1 784 (10.35)	17 240 (100)	

*Data are presented as n (%), mean ±SD or median (q1 - q3)..
#Crude, Univariate Model, OR (95% CI), Odds Ratio with 95% Wald, Confidence Limits using proc logistic, proc hplogistic.
**Lodz Voivodeship known as Lodz province (Wikipedia): https://en.wikipedia.org/wiki/%C5%81%C3%B3d%C5%BA_Voivodeship
ATC group - Anatomical Therapeutic Chemical (ATC) Classification
R03 - drugs for obstructive airway diseases
R03A - adrenergics, inhalants:
*R03AC - Selective beta-2-adrenoreceptor agonists
*R03AK - Adrenergics in combination with corticosteroids or other drugs, excl. anticholinergics
*R03AL - Adrenergics in combination with anticholinergics incl. triple combinations with corticosteroids
R03B - other drugs for obstructive airway diseases, inhalants:
*R03BA – Glucocorticoids
*R03BB - Anticholinergics
R03D - other systemic drugs for obstructive airway diseases:
*R03DA - Xanthines
*R03DC - Leukotriene receptor antagonists
https://indeks.mp.pl/leki/find.php?kind=atc&subatc=1&id=R03
- NMiss=1906 (11.84%) of missing data per each of the 7 variables with drugs:*R03AC, *R03AK, *R03AL, *R03BA, *R03BB, *R03DA, *R03DC.

For peer review only - http://bmjopen.bmj.com/site/about/guidelines.xhtml

Table 2A Characteristic of COPD patients with and without exacerbations (After PSM).

Characteristic	COPD patients without exacerbations (n=1 492 pairs) Control (J44) n(%)	COPD patients with exacerbations (n=1 492 pairs) Case (Hospitalized due to J44) n(%)	Total patients (n=2 984) n(%)	OR (95% Wald Confidence Limits). (Point Estimate.) OR (95% CI) - Univariate analysis# Paired LR
**Total**	1 492 (50)	1 492 (50)	2 984 (100)	
**Exacerbation number**				Data only for the group with exacerbations.
**1**	0 (0)	1 294 (86.73)	1 294 (8.04)	
**2**	0 (0)	148 (9.92)	148 (0.92)	
**3**	0 (0)	32 (2.14)	32 (0.2)	
**4**	0 (0)	11 (0.74)	11 (0.07)	
**5**	0 (0)	5 (0.34)	5 (0.03)	
**6**	0 (0)	2 (0.13)	2 (0.01)	
**Total**	1 492 (50)	1 492 (50)	2 984 (100)	
**Age of the patient 65+ (years) Median(q1-q3)**	75 (69.00- 81.00)	75 (69.00-81.00)	75 (69.00-81.00)	PSM NS LR No Effect after PSM
**Number of refunded drugs packages (ATC groups) (Missing 7.37%) Avg±SD)**				
**R03AC***	4.65±±5.63	6.08±6.95	5.4±6.39	1.037 [1.023,1.051]
**R03AK***	2.01±3.9	2.46±3.99	2.25±3.95	1.028 [1.007,1.049]
**R03AL***	0.95±4.18	3.41±8.99	2.24±7.21	1.087 [1.064,1.11]
**R03BA***	2.38±4.25	3.55±5.72	3±5.1	1.051 [1.033,1.069]
**R03BB***	4.47±5.83	6.38±7.3	5.46±6.71	1.049 [1.035,1.064]
**R03DA***	3.06±5.12	4.01±5.12	3.56±5.14	1.038 [1.022,1.055]
**R03DC***	0.25±1.45	0.28±1.52	0.26±1.49	0.997 [0.945,1.051]
**Total NFZ cost for, all current and settled services in 2016, regardless of the main diagnosis and reimbursement of drugs from prescriptions completed in 2016**				
**high (>21 056.52)**	186 (12.47)	560 (37.53)	746 (25)	42.804 [28.145,65.097]
**medium <2 497.62-8 264.65>**	618 (41.42)	875 (58.65)	1 493 (50.03)	18.503 [12.627,27.115]
**low (<2 497.62)**	688 (46.11)	57 (3.82)	745 (24.97)	1 Ref. Cat.
**Total**	1 492 (50)	1 492 (50)	2 984 (100)	

*Data are presented as n (%), mean ±SD or median (q1q3).
IQR – interquartile range

For peer review only - http://bmjopen.bmj.com/site/about/guidelines.xhtml

Table 3A Costs for the raw data (Raw data before PSM, n=16 099) of COPD patients with and without exacerbations.
Cost indicators (%) were calculated as contribution of patient costs with exacerbations (or without) among the cost for all COPD patients.

Characteristic Costs n(%) N=16099	COPD without exacerbations (n=14 607)	COPD with exacerbations (n=1 492)	Total (n=16 099)	p-value - Univariate analysis# HP Logistic Regression
**Total J44 Patients Sum (%)**	14 607 (90.73)	1 492 (9.27)	16 099	
**Total stationary costs (%)***				
**high**	3189 (21,83)	838 (56,17)	4027 (25,01)	<.0001
**medium**	1756 (12,02)	642 (43,03)	2398 (14,9)	<.0001
**low**	9662 (66,15)	12 (0,8)	9674 (60,09)	Ref. Cat.
**Total**	14607 (90,73)	1492 (9,27)	16099 (100)	
**Total cost of reimbursed drugs (%)***				
**high**	3434 (23,51)	590 (39,54)	4024 (25)	<.0001
**medium**	7363 (50,41)	687 (46,05)	8050 (50)	<.0001
**low**	3810 (26,08)	215 (14,41)	4025 (25)	Ref. Cat.
**Total**	14607 (90,73)	1492 (9,27)	16099 (100)	
**TOTAL NFZ cost (%)***				
**high**	3191 (21,85)	833 (55,83)	4024 (25)	<.0001
**medium**	7398 (50,65)	653 (43,77)	8051 (50,01)	<.0001
**low**	4018 (27,51)	6 (0,4)	4024 (25)	Ref. Cat.
**Total**	14607 (90,73)	1492 (9,27)	16099 (100)	

*Costs were calculated as the proportion of costs per patient in the mean of the COPD patient population [%], and categorized by lower (q1) and upper (q3) quartiles.
q1-Lower Quartile; q3-Upper Quartile, CL-Confidence Limit, HP LR-High Performance Logistic Regression.
N=16099
Total stationary costs (%) q1=0.00 q3=101.72
Total cost of reimbursed drugs (%) q1=34.75 q3=141.10
TOTAL NFZ cost (%) q1=28.71 q3=111.69

For peer review only

Table 3B Costs for the data after PSM transformation (N=1492 pairs) of COPD patients with and without exacerbations.

Characteristic Costs n(%) N=1492 pairs	COPD patients without exacerbations (n=1492 pairs) n(%)	COPD patients with exacerbations exacerbations (n=1492 pairs) n(%)	TotalAll patients (n=2984) n(%)	p-value - Univariate analysis Conditional Logistic Regression for Matched Pairs
**Total J44 Patients Sum (%)**	1492 (50)	1492 (50)	2984 (100)	
**Total stationary costs (%)***				
**high**	168 (11,26)	564 (37,8)	732 (24,53)	<.0001
**medium**	332 (22,25)	916 (61,39)	1248 (41,82)	<.0001
**low**	992 (66,49)	12 (0,8)	1004 (33,65)	Ref. Cat.
**Total**	1492 (50)	1492 (50)	2984 (100)	
**Total cost of reimbursed drugs (%)***				
**high**	270 (18,1)	476 (31,9)	746 (25)	<.0001
**medium**	748 (50,13)	744 (49,87)	1492 (50)	<.0001
**low**	474 (31,77)	272 (18,23)	746 (25)	Ref. Cat.
**Total**	1492 (50)	1492 (50)	2984 (100)	
**TOTAL NFZ cost (%)***				
**high**	186 (12,47)	560 (37,53)	746 (25)	<.0001
**medium**	618 (41,42)	875 (58,65)	1493 (50,03)	<.0001
**low**	688 (46,11)	57 (3,82)	745 (24,97)	Ref. Cat.
**Total**	1492 (50)	1492 (50)	2984 (100)	

*Costs were calculated as the proportion of costs per patient in the mean of the COPD patient population [%], and categorized by lower (q1) and upper (q3) quartiles.
q1-Lower Quartile; q3-Upper Quartile, CL-Confidence Limit.
N=1492 pairs
Total stationary costs (%) q1=0.00 q3=111.56
Total cost of reimbursed drugs (%) q1=37.19 q3=142.19
TOTAL NFZ cost (%) q1=34.23 q3=113.27

For peer review only - http://bmjopen.bmj.com/site/about/guidelines.xhtml

Big Data Quality (Big Data Cleansing) Results: for Medical (NFZ) and Business (Tax Office) Data.

Files were obtained from Big Data databases (NFZ, Tax Office and GUS). In total, data for n = 472,314 cohorts of 65+ patients registered in primary health care (PHC) clinics were obtained from electronic medical records (EHR) of NFZ in the Lodz province. In total, n = 439,577 inhabitants 65+ living in the Lodz province were obtained from the databases of 28 Tax Offices located in the Lodz province. Variables for n = 177 communes of the Lodz province, which, according to the literature review, were related to exacerbations were collected from the GUS databases. Patient data from NFZ showed that the identifier has no duplicates. "Codes of comorbidities with COPD" had 98.8% of missing data (NFZ) out of 472 314 in total 65+ patients in PHC in Lodz province in 2016, and the variable "ICD9 procedures for other stays" had 98.1% of missing data (NFZ). Only 0.1% was missing in the variable "Postal code for 65+ residents" in Tax Office databases. Data on air quality for the group "Air pollutant emission from particular nuisance plants (G216)" consisting of 6 subgroups were not available at the level of data sharing for gmina level for 2016 in the GUS database (accessed on November 3, 2021) .

Propensity score matching (PSM) - before and after - overlay histograms age, gender

Fig. 1 Histogram for the age before PSM.

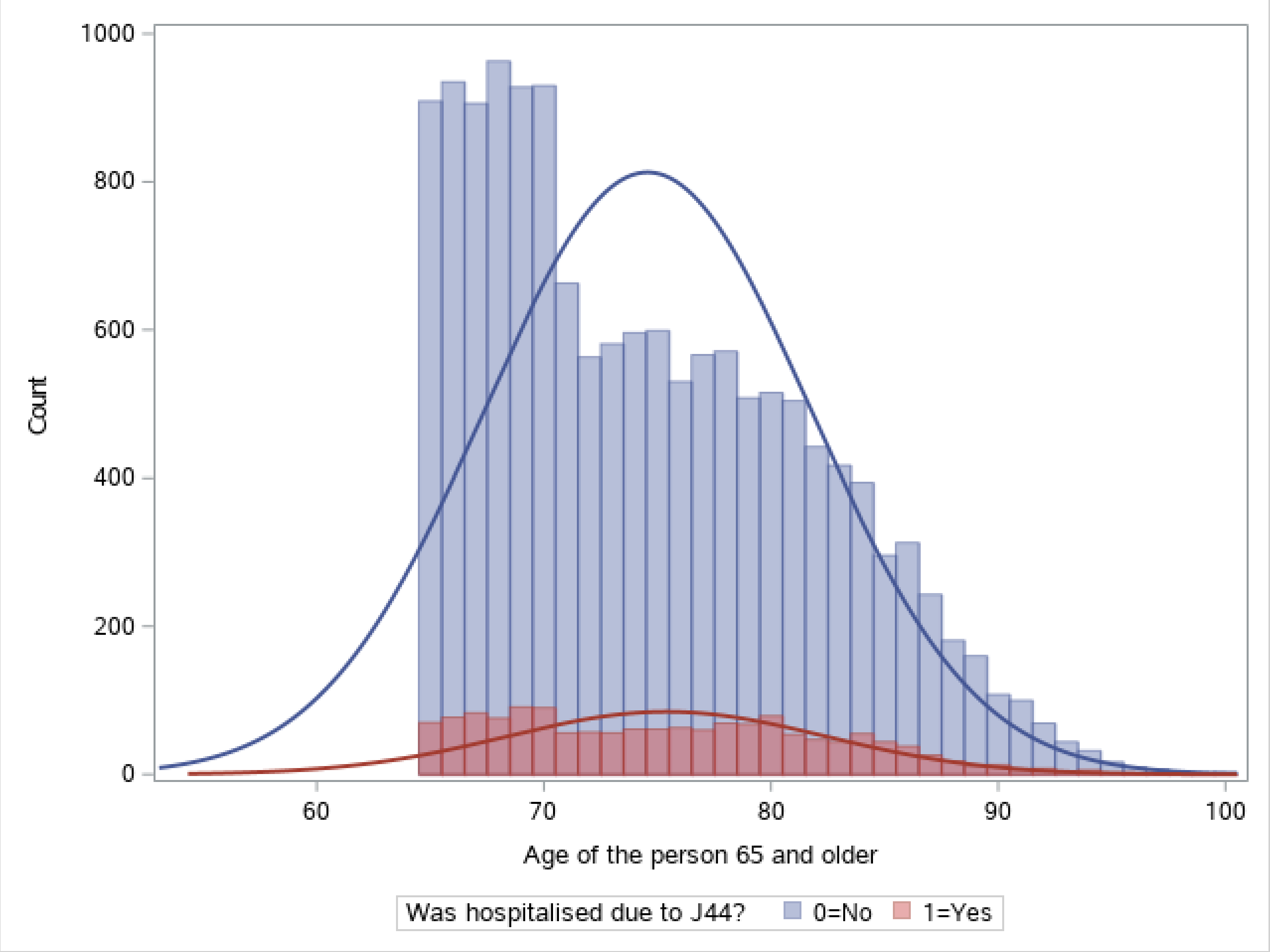

For peer review only - http://bmjopen.bmj.com/site/about/guidelines.xhtml

Fig. 2 Histogram for the age after PSM.

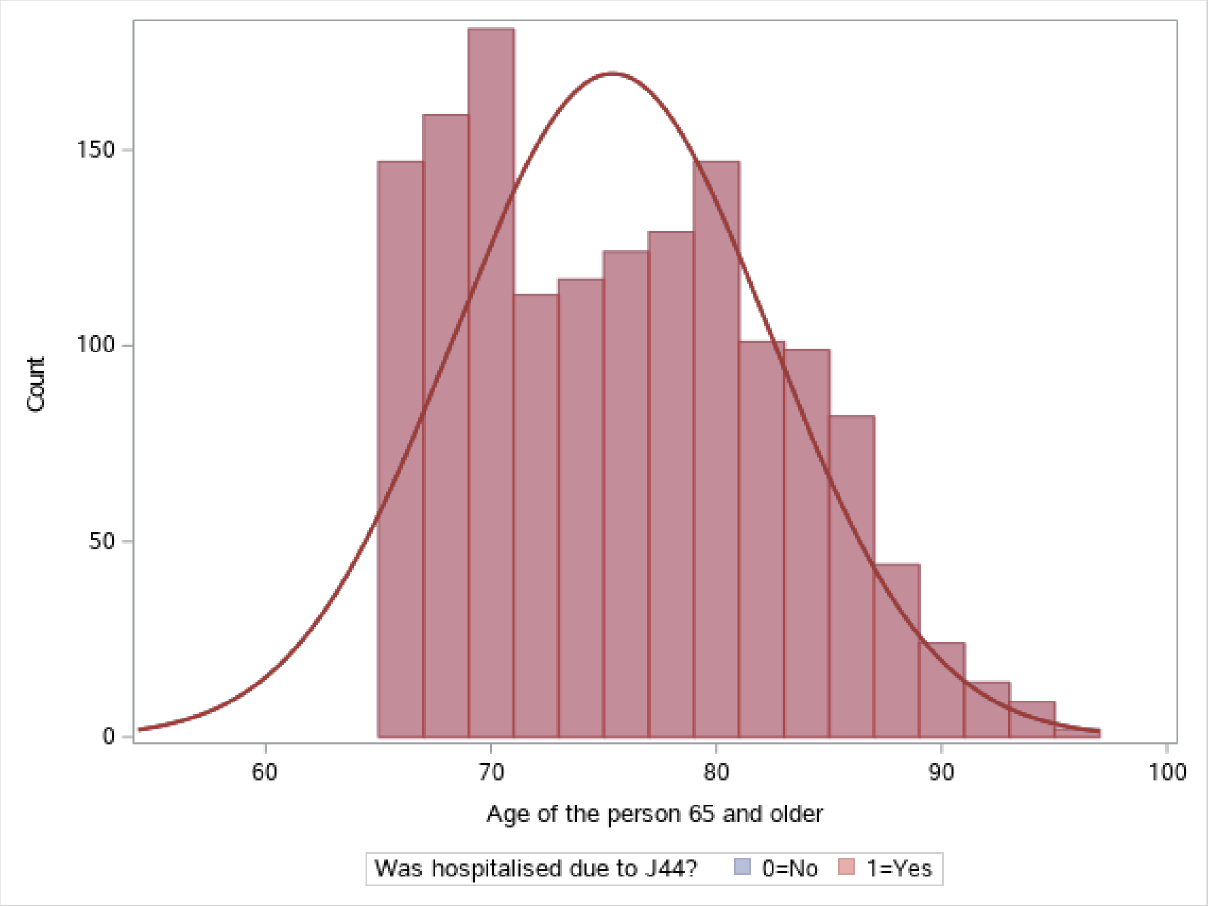

For peer review only - http://bmjopen.bmj.com/site/about/guidelines.xhtml

Fig. 3 Histogram for the gender before PSM.

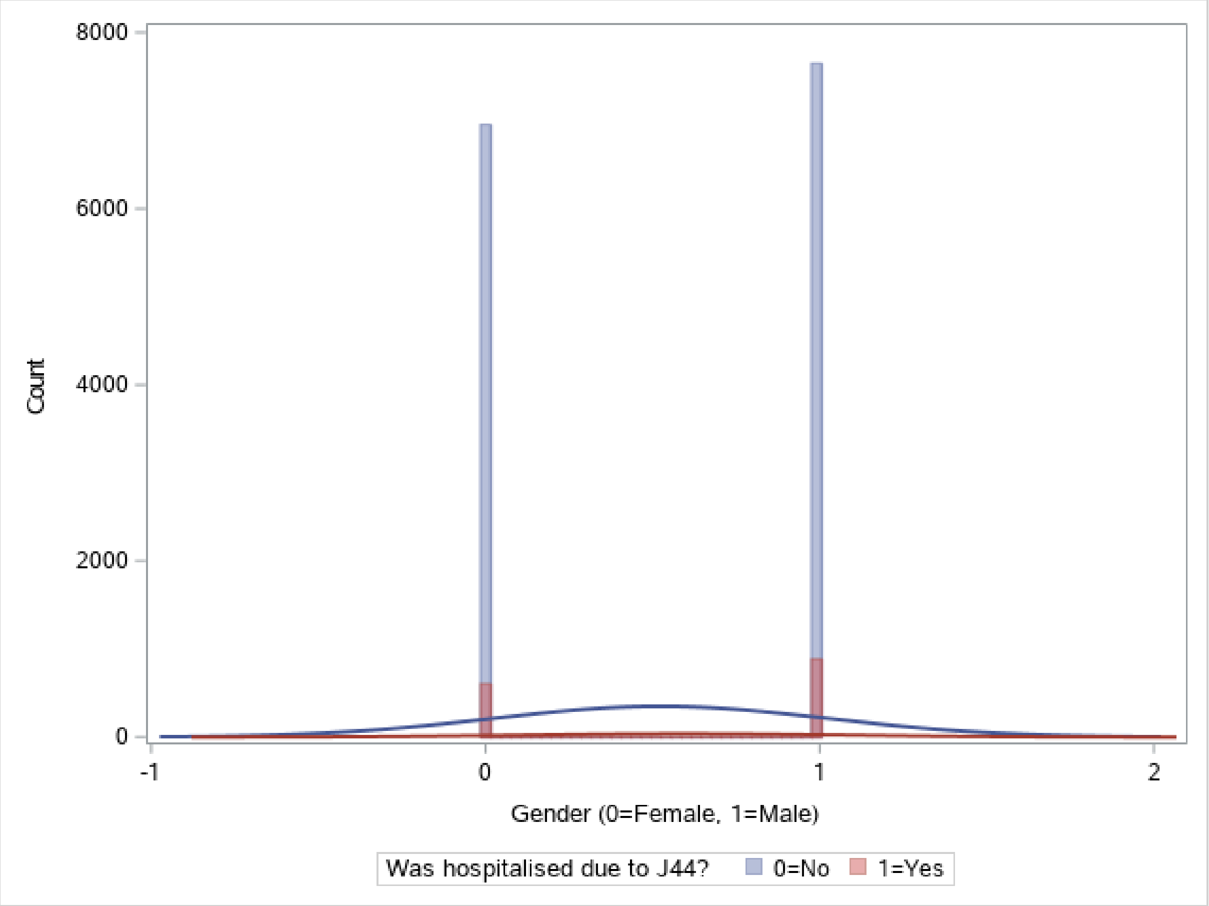

For peer review only - http://bmjopen.bmj.com/site/about/guidelines.xhtml

Fig. 4 Histogram for the gender after PSM.

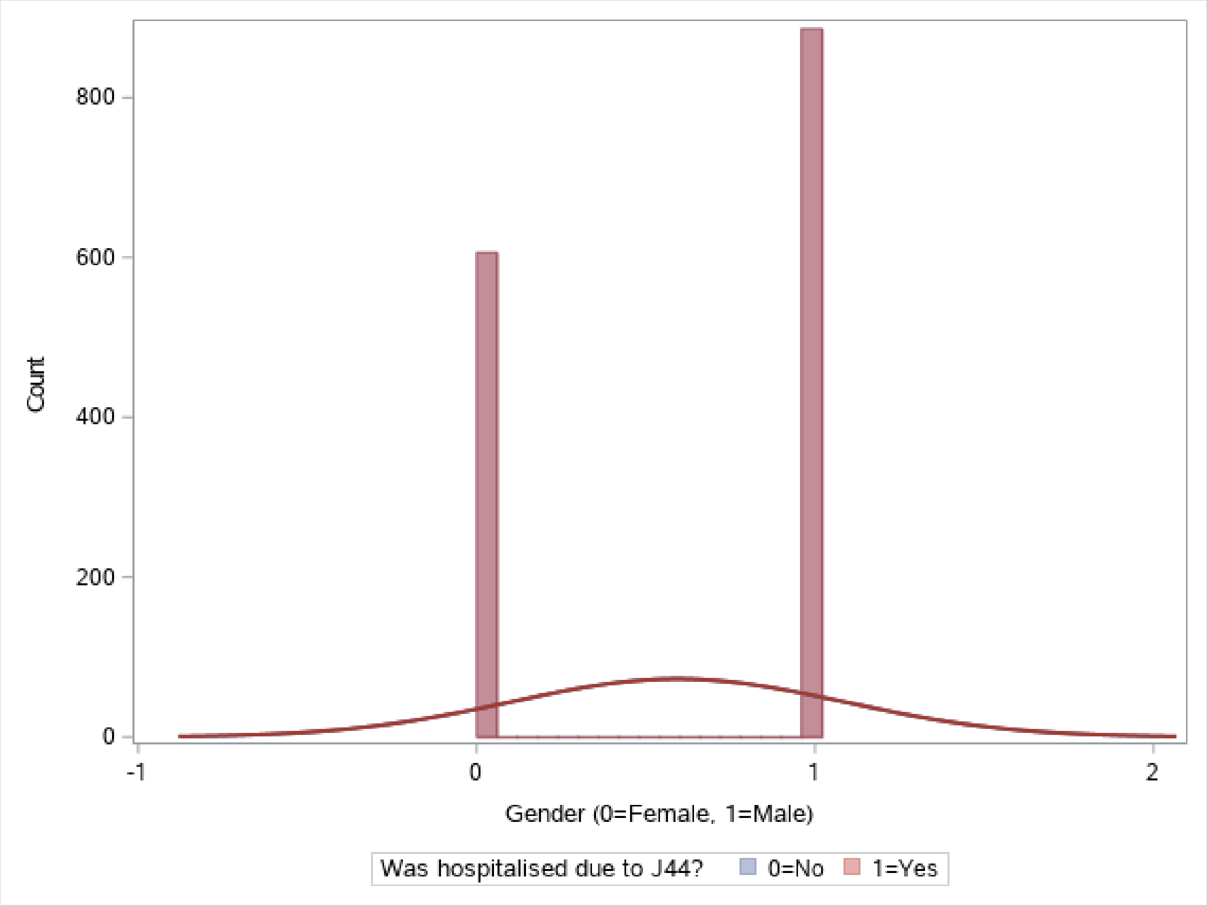

For peer review only - http://bmjopen.bmj.com/site/about/guidelines.xhtml

STROBE Statement—checklist of items that should be included in reports of observational studies

Title: Community determinants of COPD exacerbations in elderly patients in Lodz Province, Poland: a retrospective observational Big Data cohort study

	Item No	Recommendation	Page No
**Title and abstract**	1	(*a*) Indicate the study's design with a commonly used term in the title or the abstract	2-4
		(*b*) Provide in the abstract an informative and balanced summary of what was done and what was found	3-4
**Introduction**			
Background/rationale	2	Explain the scientific background and rationale for the investigation being reported	5-7
Objectives	3	State specific objectives, including any prespecified hypotheses	7
**Methods**			
Study design	4	Present key elements of study design early in the paper	7
Setting	5	Describe the setting, locations, and relevant dates, including periods of recruitment, exposure, follow-up, and data collection	7-8
Participants	6	(*a*) *Cohort study*—Give the eligibility criteria, and the sources and methods of selection of participants. Describe methods of follow-up<br>*Case-control study*—Give the eligibility criteria, and the sources and methods of case ascertainment and control selection. Give the rationale for the choice of cases and controls<br>*Cross-sectional study*—Give the eligibility criteria, and the sources and methods of selection of participants	8
		(*b*) *Cohort study*—For matched studies, give matching criteria and number of exposed and unexposed<br>*Case-control study*—For matched studies, give matching criteria and the number of controls per case	8-9
Variables	7	Clearly define all outcomes, exposures, predictors, potential confounders, and effect modifiers. Give diagnostic criteria, if applicable	8-9, Supplementary 32-33
Data sources/ measurement	8*	For each variable of interest, give sources of data and details of methods of assessment (measurement). Describe comparability of assessment methods if there is more than one group	7-8
Bias	9	Describe any efforts to address potential sources of bias	21
Study size	10	Explain how the study size was arrived at	9-10
Quantitative variables	11	Explain how quantitative variables were handled in the analyses. If applicable, describe which groupings were chosen and why	8-9

For peer review only - http://bmjopen.bmj.com/site/about/guidelines.xhtml

Statistical methods	12	(*a*) Describe all statistical methods, including those used to control for confounding	8-9
		(*b*) Describe any methods used to examine subgroups and interactions	-
		(*c*) Explain how missing data were addressed	9
		(*d*) *Cohort study*—If applicable, explain how loss to follow-up was addressed	8-9
		Case-control study—If applicable, explain how matching of cases and controls was addressed	8-9
		Cross-sectional study—If applicable, describe analytical methods taking account of sampling strategy	8-9
		(*e*) Describe any sensitivity analyses	-
**Results**			
Participants	13*	(a) Report numbers of individuals at each stage of study—eg numbers potentially eligible, examined for eligibility, confirmed eligible, included in the study, completing follow-up, and analysed	10
		(b) Give reasons for non-participation at each stage	-
		(c) Consider use of a flow diagram	-
Descriptive data	14*	(a) Give characteristics of study participants (eg demographic, clinical, social) and information on exposures and potential confounders	10-17 Supplementary 28-29, 30-31 APPENDIX 34-37, 38-40
		(b) Indicate number of participants with missing data for each variable of interest	APPENDIX 34-36, 38
		(c) *Cohort study*—Summarise follow-up time (eg, average and total amount)	10-13
Outcome data	15*	*Cohort study*—Report numbers of outcome events or summary measures over time	10-13
		Case-control study—Report numbers in each exposure category, or summary measures of exposure	13-16
		Cross-sectional study—Report numbers of outcome events or summary measures	10-16
Main results	16	(*a*) Give unadjusted estimates and, if applicable, confounder-adjusted estimates and their precision (eg, 95% confidence interval). Make clear which confounders were adjusted for and why they were included	10, 17-19
		(*b*) Report category boundaries when continuous variables were categorized	Supplementary 28-29, 30-33 APPENDIX 39-40
		(*c*) If relevant, consider translating estimates of relative risk into absolute risk for a meaningful time period	-
Other analyses	17	Report other analyses done—eg analyses of subgroups and interactions, and sensitivity analyses	-
**Discussion**			
Key results	18	Summarise key results with reference to study objectives	20-21

For peer review only - http://bmjopen.bmj.com/site/about/guidelines.xhtml

Limitations	19	Discuss limitations of the study, taking into account sources of potential bias or imprecision. Discuss both direction and magnitude of any potential bias	21-22
Interpretation	20	Give a cautious overall interpretation of results considering objectives, limitations, multiplicity of analyses, results from similar studies, and other relevant evidence	19-23
Generalisability	21	Discuss the generalisability (external validity) of the study results	23
**Other information**			
Funding	22	Give the source of funding and the role of the funders for the present study and, if applicable, for the original study on which the present article is based	23

For peer review only

For peer review only - http://bmjopen.bmj.com/site/about/guidelines.xhtml
